# Feedback, Lineages and Self-Organizing Morphogenesis

**DOI:** 10.1371/journal.pcbi.1004814

**Published:** 2016-03-18

**Authors:** Sameeran Kunche, Huaming Yan, Anne L. Calof, John S. Lowengrub, Arthur D. Lander

**Affiliations:** 1 Department of Biomedical Engineering, University of California, Irvine, Irvine, California, United States of America; 2 Center for Complex Biological Systems, University of California, Irvine, Irvine, California, United States of America; 3 Department of Mathematics, University of California, Irvine, Irvine, California, United States of America; 4 Department of Developmental and Cell Biology, University of California, Irvine, Irvine, California, United States of America; 5 Department of Anatomy and Neurobiology, University of California, Irvine, Irvine, California, United States of America; Northeastern University, UNITED STATES

## Abstract

Feedback regulation of cell lineage progression plays an important role in tissue size homeostasis, but whether such feedback also plays an important role in tissue morphogenesis has yet to be explored. Here we use mathematical modeling to show that a particular feedback architecture in which both positive and negative diffusible signals act on stem and/or progenitor cells leads to the appearance of bistable or bi-modal growth behaviors, ultrasensitivity to external growth cues, local growth-driven budding, self-sustaining elongation, and the triggering of self-organization in the form of lamellar fingers. Such behaviors arise not through regulation of cell cycle speeds, but through the control of stem or progenitor self-renewal. Even though the spatial patterns that arise in this setting are the result of interactions between diffusible factors with antagonistic effects, morphogenesis is not the consequence of Turing-type instabilities.

## Author Summary

A large variety of mechanisms have been proposed to explain how the cells of solid tissues work together to produce distinctive structures such as buds, branches, sacs and tubes. Here we identify a novel mechanism that emerges directly out of the processes that cells use to control proliferation and maintain overall tissue sizes. In particular, we focus on the diffusible molecules that regulate lineage progression—the decision by dividing cells either to renew themselves or produce cells of a more differentiated type. When such molecules are produced by cells at specific lineage stages, they create feedback circuits, and positive and negative lineage feedback circuits are often observed together in the same tissue. Here we show that the combination of positive and negative lineage feedback can lead to the emergence of patterned growth from which sharply-demarcated buds and fingers readily emerge, either spontaneously or in response to transient external signals. We show that the types of behaviors that emerge, and the conditions under which they do so, depend strongly on the relative strengths of positive and negative feedback.

## Introduction

Diffusible molecules, such as morphogens, growth factors, cytokines and chemokines, play many important roles in solid tissues, among which are conveying positional information, coordinating proliferation with differentiation, and enabling collective responses to disturbances [1–6]. Less clear, however, is the importance of diffusible signals in orchestrating the morphogenesis of solid tissues—i.e. the formation of three-dimensional structures such as sacs, tubes, sheets, buds and branches. Typically, the events of morphogenesis are described in terms of intrinsic cellular behaviors, such as cell migration [7], localized changes in cell shape [8–12], differential proliferation [13, 14], regulated cleavage plane orientation [15–18], differential cell adhesion [19], and mechanical force production [20]. How much of a role long-range diffusible cues play in organizing these processes is poorly understood.

Recent mathematical modeling has suggested that the ability of diffusible molecules to spontaneously create large-scale spatial patterns (“Turing patterns” [21, 22]) might drive branching morphogenesis in the lung [23]. Turing patterns have also been implicated in digit morphogenesis in vertebrate limbs [24]. As the necessary conditions for Turing patterning are relatively restrictive—for example, pairs of factors that differ greatly in their diffusivity are required—how commonly this mechanism is exploited in morphogenesis remains to be seen.

Here we identify a different mechanism by which diffusible signals can drive morphogenesis—not one based on creating patterns that act upstream of tissue growth or cell movement, but on regulating cell lineage progression, so that patterns arise directly out of tissue growth and cell displacement. As we describe below, such interactions can create large-scale spatial structure, and generate sharply-demarcated tissue forms, both spontaneously and in response to graded and/or transient extrinsic cues.

Underlying such behavior is a negative feedback loop that has primarily been studied for its role in size homeostasis of continually self-renewing tissues [2, 25–27]. The basis of this mechanism is the production of diffusible molecules by differentiated cells that act upon stem or progenitor cells to decrease the probability of self-renewal, and increase the probability of differentiation. In muscle, for example, such feedback comes from GDF8/myostatin [28, 29], whereas in the olfactory epithelium the related molecules GDF11 and activin play an analogous role [30, 31]. Such feedback causes stem cell populations to automatically coordinate their behaviors so that net renewal is maintained at exactly 50%—the value required for homeostasis [25, 32]. Feedback of this type also produces steady states that are “perfectly” robust, insofar as tissue size is maintained at a set-point independent of initial conditions, cell cycle speed, or rates of cell turnover [25].

During development, the regulation of tissue and organ growth is less a matter of achieving homeostasis, than of efficiently arriving at a correct final size and shape (i.e., morphogenesis). Indeed, for the many organs in which cell turnover is non-existent or negligible in the adult, a controlled growth trajectory during development is the only possible function of feedback regulation. Fortunately, mathematical modeling shows that negative feedback control of self-renewal, the same strategy that achieves robust steady states in tissues that turn over, can also achieve nearly perfectly robust final states for tissues that do not turn over [25]. Indeed, feedback molecules such as GDF8, GDF11, myostatin and other renewal-inhibiting factors are often highly expressed during development. Yet, interestingly, such molecules are often co-expressed with other diffusible molecules that have exactly the opposite effect on stem and progenitors cells—promoting renewal at the expense of differentiation. In the developing olfactory epithelium, for example, the same cells that produce GDF11, which inhibits the renewal of progenitor cells, also produce FGF ligands that stimulate renewal of the same cells [33]. In muscle, production of renewal-inhibiting GDF8 is similarly accompanied by production of renewal-stimulating FGFs [34]. In other tissues, pairs of co-expressed renewal-promoting and -inhibiting factors include Wnts and BMPs [35]; HGF and TGFβ [36]; and PTHrP and FGF [37].

Mixing positive and negative feedback might at first seem counterproductive, but it is well known that countervailing feedback loops—if they are non-linear and operate on sufficiently well-separated temporal or spatial scales—can give rise to interesting dynamic or spatially-dynamic behaviors. Indeed, Turing patterns may be seen as a special case of this general phenomenon.

Here we show that the simultaneous positive and negative feedback regulation of cell renewal by diffusible signals tends naturally to lead to the spontaneous emergence of spatially dynamic behaviors, which manifest as sharply localized differences in net growth. We mathematically define the conditions required for underlying phenomena such as bistability, ultrasensitivity and spatial self-organization to arise in such systems, and show how they can be used to drive the formation of induced or spontaneous bulges, buds and fingers (the basic building blocks of many types of morphogenesis). We further show that even though such phenomena arise out of the opposing interactions of diffusible factors, the patterns that arise are not Turing patterns. Rather, pattern formation is driven by the coupling between tissue growth and the inherent ultrasensitivity of the underlying system, and not by intrinsic differences in the spatial ranges of action of diffusible positive and negative signals.

## Results

### Modeling lineage feedback

Cell lineages may be described as pathways of differentiation that begin with a stem cell (SC), progress through some number of self-renewing progenitor stages, and end with one or more terminally differentiated (TD) cells. Here we consider only the simplest of lineages—ones that are un-branched uni-directional, and produce TD cells that are non-dividing (postmitotic)—because, as we will see, the spontaneous emergence of morphogenetic behaviors requires no more complexity than that.

Following [25], the dynamics of such lineages may be written as a system of ordinary differential equations (ODEs), shown in section 1 of S1 Text, in which cell number (or concentration) at any lineage stage *i* is represented by the variable *χ*_*i*_, cell cycle speed at stage *i* by rate parameter *v*_*i*_, and the probability of self-renewal at stage *i* by parameter *p*_*i*_ (0 ≤ *p*_*i*_ ≤ 1). In tissues undergoing cell turnover, a rate constant, *d*, for cell loss or death must also be introduced (minimally for the TD cell). Within this framework, feedback or feedforward effects may be represented as dependencies of parameters upon the *χ*_*i*_.

When seeking to capture the effects of diffusible molecules, modeling with ODEs is equivalent to assuming that cells are homogenously distributed (“well-stirred”) in space, so that diffusion gradients may be ignored. Although this is not generally a valid assumption for solid tissues, we may still understand the ODE representation of lineage dynamics as applying to sufficiently small spatial domains within tissues. In addition, because solutions to deterministic ODEs do not reveal the effects of stochastic variation that one might expect to encounter at low cell numbers, their analysis must often be supplemented with independent stochastic simulations,. In spite of these limitations, ODE formulations can serve as a good starting point for developing general insights, which can then be extended to regimes in which stochasticity, space and spatial organization matter explicitly. This is the approach we take here.

### Mixed feedback in continually-renewing, well-stirred lineages

**Fig 1:**
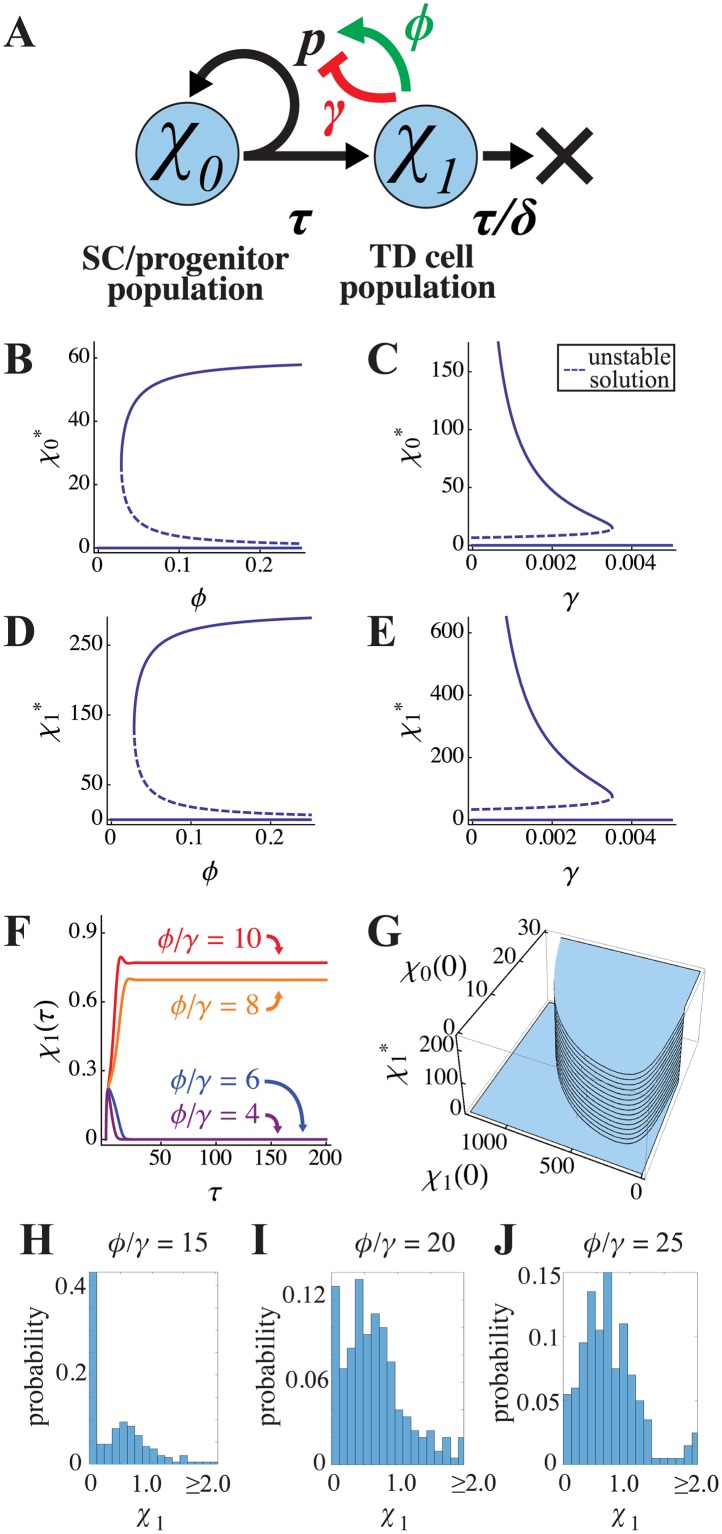
Bistability of a two-stage lineage. **(A)** Illustration of a lineage consisting of stem/progenitor cells (*χ*_0_) and terminally differentiated (TD) cells (*χ*_1_), in which feedback promotes self-renewal (*p*) with strength *ϕ* and inhibits it with strength *γ*. The parameter *τ* = 1/*v* denotes the cell division time (*v* is the rate of cell division) and *τ/δ* represents the lifetime of TD cells, in units of *τ* (*δ* = *d*/*v* where *d* is the TD death rate). **(B-E)** This feedback configuration yields bifurcations and bistability in both the SC/CP and TD cell equilibrium populations. Parameter values for these curves were *δ* = 0.2, *p* = 0.8. **(F)** Steady states are determined by the *ϕ*/*γ* ratio, for which the threshold for non-zero growth is approximately 5.8 when *p* = 1. Here, parameter values were *δ* = 1, *p* = 1, and *γ* = 1; *ϕ* was varied as shown. The initial conditions were *χ*_0_(0) = 0.3 and *χ*_1_(0) = 0. **(G)** Small changes in initial conditions can determine which steady state is reached. A larger number of initial progenitors *χ*_0_(0), or an initial *χ*_1_(0) that is neither too large nor too small, favors attainment of the non-zero steady state. **(H-J)** The probability distributions of *χ*_1_ after 200 CP cell cycles, obtained from simulations in which both the self-renewal parameter (*p*), and the initial conditions for *χ*_0_ were taken to be normally-distributed random variables with means *p* = 0.9 and *χ*_0_(0) = 0.3. The variances in both cases were 0.1; *p* was truncated to ensure it was between 0 and 1 and *χ*_0_ was truncated to ensure it was positive. These variables were sampled every CP cycle and 200 realizations were performed. The results are shown for three different values of *ϕ*/*γ*, as labeled. Other parameters were as in panel F.

Fig 1A depicts the simplest possible, continually-renewing unidirectional lineage: a single type of SC that gives rise directly to a single type of TD cell that then turns over with a constant half-life. The ODEs for this situation may be written as

$$\begin{array}{l} \frac{d\chi_{0}(\tau )}{d\tau }=(2P(\tau )-1)\chi_{0}(\tau )\\ \frac{d\chi_{1}(\tau )}{d\tau }=2\left(1-P(\tau )\right)\chi_{0}(\tau )-\delta \chi_{1}(\tau ) \end{array}$$ (1)

in which *χ*_0_ and *χ*_1_ represent SC (cell type “0”) and TD (cell type “1”) cells, respectively; *P* stands for the SC renewal probability; *τ* refers to time in units of cell cycles; and *δ* is the death rate of TD cells relative to the cell cycle rate, i.e. *δ* = *d*/*v*. To represent the possibility that TD cells may be a source of both negative and positive feedback on *P*, we replace *P* with the expression $p\left(\frac{1}{1+\gamma \;\chi_{1}(\tau )}\right)\left(\frac{\varphi \;\chi_{1}(\tau )}{1+\varphi \;\chi_{1}(\tau )}\right)$, where *γ* and *ϕ* quantify the strength of the negative and positive feedback, respectively, while *p*, a constant between 0 and 1, represents the maximum possible renewal probability. Expressing feedback as a product of Hill functions is convenient insofar as it guarantees a non-negative value of *P*, and exhibits saturation at large values of *χ*_1_, as would be expected for any biological process. However, the precise form of the feedback function has only minor effects on the qualitative results described below. Likewise, the interaction between negative and positive feedback terms can be represented in a variety of other ways without altering the qualitative behaviors that are observed (see Tables A and B in section 5 of S1 Text, which summarize outcomes of different forms of the feedback functions).

For non-zero values of *ϕ*, system 1, with *P* is defined as above, has three equilibrium states. In these states *χ*_1_ is equal to either

$$0,\frac{-\gamma -\varphi +2p\varphi -\sqrt{-4\gamma \varphi +{\left(\gamma +\varphi -2p\varphi \right)}^{2}}}{2\gamma \varphi },\text{or}\frac{-\gamma -\varphi +2p\varphi +\sqrt{-4\gamma \varphi +{\left(\gamma +\varphi -2p\varphi \right)}^{2}}}{2\gamma \varphi },$$

and *χ*_0_ is simply *δ* times the value of *χ*_1_ in each state, which is a consequence of the non-zero *χ*_0_ equilibrium solutions necessitating that *P* = 0.5. For trivial reasons the solution in which both *χ*_0_ and *χ*_1_ are zero (i.e. both cell types become extinct) is necessarily stable. For the non-zero solutions, a saddle-node bifurcation occurs so that the second solution is unstable, while the third solution locally stabilizes as shown in Fig 1B–1E. The criterion for bistability—in which either of the two stable states is available for a single set of parameters—is the condition that *p*>0.5 (stem cells must be capable of renewing more than 50% of the time) and:

$$\frac{\varphi }{\gamma }>\frac{1+2\sqrt{2p}+2p}{1-4p+4p^{2}}.$$

Using the maximum feasible value of 1 for *p*, we find the minimum of this ratio to be ≈5.8. In other words, bistability requires that positive feedback on progenitor self-renewal be at least 5.8 times stronger than negative feedback.

Fig 1F plots the trajectories of solutions with different *ϕ*/*γ* ratios, starting from an initial condition of zero TD cells. In every case the system starts to generate TD cells, but when *ϕ*/*γ* is not large enough, TD production collapses and both cell types vanish. The switch between extinction and maintaining a non-zero steady state occurs, as predicted, at *ϕ*/*γ* ≈5.8, and this holds regardless of the individual values of *ϕ* and *γ* (see S1 Fig).

The fact that extinction occurs when positive feedback is too weak may seem surprising, given that spontaneous extinction was not observed in prior modeling studies that considered only negative feedback (e.g. [25]), but this is simply because such studies had to proceed from the assumption that SC are intrinsically specified to renew with high probability when there is no feedback. Here, we abandon that assumption, allowing the renewal and differentiation behaviors of SC to be largely determined by extrinsic factors, as is likely to be the case in vivo.

Although the existence of a non-zero steady state depends strictly on *ϕ*/*γ*, whether that state is reached is a function of initial conditions, as shown in Fig 1G. This is to be expected for a bistable system. If SC numbers are initially too low, only extinction is possible. If initial SC numbers exceed a threshold, the non-zero steady state can be reached only if the initial number of TD cells is neither too high nor too low. This behavior can be rationalized: if TD numbers are too low, there is insufficient positive feedback to keep SC numbers from contracting to the point where SCs are incapable of sustaining themselves; if TD numbers are too high, the effect of positive feedback approaches saturation and negative feedback dominates, driving SC self-renewal below the threshold for sustainability.

To explore the effects of stochastic fluctuations, we allow *p*, the maximum possible self-renewal fraction to be a random variable, while maintaining the same form of *P* given above. The reason for focusing on the self-renewal parameter is that the greatest source of stochasticity is likely to come from randomness in cell-division outcomes, whereby cells choose probabilistically between whether to generate zero, one or two differentiated progeny. In Fig 1H–1J, we take *p = 0*.*9+*ζ, where ζ a random variable sampled every CP cell cycle from a normal distribution with mean zero and variance 0.2 (*p* is truncated to ensure it lies between 0 and 1). The results, in terms of the distributions of the TD cell numbers (*χ*_1_) after 100 CP cell cycles, measured over a large number of independent trials, are presented in Fig 1H–1J for different values of *ϕ*/*γ*. In these cases, the initial conditions were also similarly distributed random variables centered around the values from Fig 1F. The results show that, when *ϕ*/*γ* is small, the most likely state is the zero steady-state (Fig 1H). When *ϕ*/*γ* is sufficiently large, the non-zero steady state is the most likely, although the distribution of populations is fairly broad (Fig 1J). For intermediate values, the distribution is bimodal with the zero and non-zero steady states being nearly equally likely (Fig 1I) due to noise-driven switching between the steady states. Because of the possibility of stochastic extinction of small populations, the values of *ϕ*/*γ* needed to achieve non-zero steady states in the stochastic case are larger than those required for the deterministic model. Nevertheless, the behaviors of the stochastic and deterministic models are qualitatively similar.

### Final-state systems

**Fig 2:**
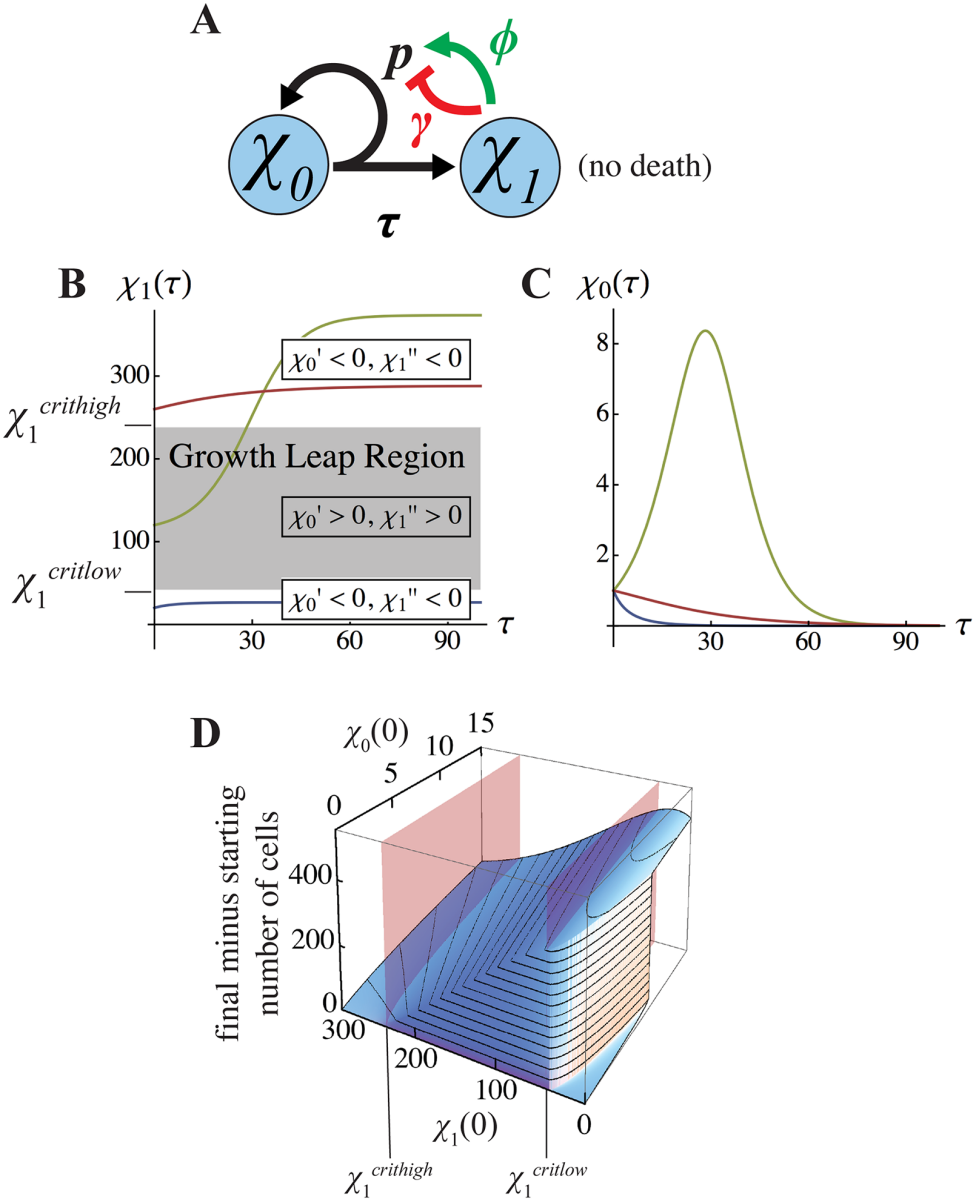
Final-state analysis of the two-stage lineage. When cell death is negligible, lineages with negative feedback on progenitor self-renewal **(A)** eventually reach a final state for *χ*_1_ **(B)** that must lie either below or above a “growth leap” region. The bounds on this region (denoted *χ*_1_^*critlow*^ and *χ*_1_^*crithigh*^), are the values of *χ*_1_ at which *χ*_0_' changes sign. As shown in **(C),** for initial values *χ*_1_(0) below *χ*_1_^*critlow*^ (blue) or above *χ*_1_^*crithigh*^ (red), SC numbers (*χ*_0_) necessarily shrink monotonically; otherwise *χ*_0_ grows transiently (green line) and then shrinks to zero. **(D)** Whether a growth leap occurs is determined by the initial values of both *χ*_0_ and *χ*_1_. Here the presence of a growth leap is manifested as a large net increase in total cell number of cells. For small enough initial values of *χ*_0_, such a leap occurs as in panel B, when initial *χ*_1_ values lie between *χ*_1_^*critlow*^ and *χ*_1_^*crithigh*^ (denoted by red planes). For larger initial values of *χ*_0_ however, the growth leap regime can be accessed at initial values of *χ*_1_ below *χ*_1_^*critlow*^. Parameter values for panels B—D are *p* = 0.8, *ϕ* = 0.05, and *γ* = 0.002. The initial conditions in B were *χ*_1_(0) = 20, 120, and 260, with *χ*_0_(0) = 1, as shown in C.

The system of ODE in (1) models lineages in which TD cells constantly turn over, as occurs in hematopoietic, epidermal and many epithelial lineages. In such a system, steady state solutions can be calculated simply by setting the time derivatives in (1) to zero. Not all tissues reach steady states, however. In some, TD cells last for the lifetime of the organism (e.g. in the nervous system), with SC either disappearing, or becoming quiescent. Such tissues may be modeled by setting the turnover rate *δ* in (1) to zero, as illustrated in Fig 2A (see also [25]). In this case, the governing equations become

$$\begin{array}{l} \frac{d\chi_{0}(\tau )}{d\tau }=(2P(\tau )-1)\chi_{0}(\tau )\\ \frac{d\chi_{1}(\tau )}{d\tau }=2\left(1-P(\tau )\right)\chi_{0}(\tau ) \end{array}$$ (2)

again allowing feedback to be captured by a dependence of *P* on the levels of cell types (e.g., we again take *P* = $p\left(\frac{1}{1+\gamma \;\chi_{1}(\tau )}\right)\left(\frac{\varphi \;\chi_{1}(\tau )}{1+\varphi \;\chi_{1}(\tau )}\right)$). The lack of TD cell turnover in (2) means that TD cells are now free to accumulate to a level that, through negative feedback, drives SC to extinction. A stationary state is thereby reached, but it is not “steady” in the usual sense of the word, i.e. there is no dynamic equilibrium; we therefore refer to it as a “final state”. Note that TD values at the final state cannot be calculated simply by setting the time derivatives in (2) to zero, because the time derivatives and SC value (*χ*_0_) both go to zero together, leaving *P* undetermined.

Strictly speaking, final-state systems require TD cells that never turn over. However, if TD cells turn over slowly, the final state formulation will provide a better description of lineage dynamics on time scales that are short relative to the TD cell half-life. As tissue morphogenesis often occurs rapidly, relative to TD cell lifespans, final state models may thus be inherently better representations of lineage dynamics during morphogenesis, even in self-renewing tissues. We therefore explored the effects of mixed feedback in final-state systems.

As with steady-state systems, we observed behaviors of final-state systems that depended strongly on the value of the ratio *ϕ*/*γ*, and exhibited abrupt switching between distinct states in response to small changes in the initial conditions for either *χ*_0_ or *χ*_1_ (see plots in Fig 2). As described in Box 1, the dynamics of TD production can be understood in relation to two critical TD population sizes: *χ*_1_^*critlow*^ and *χ*_1_^*crithigh*^. When *χ*_1_ lies between these two values, *χ*_0_ grows; otherwise, *χ*_0_ shrinks to zero. Understanding the dynamics that drive TD cell numbers across these critical values allows us to understand why initial conditions, seen in Fig 2D, cause leaps in growth to coincide with neither critical values nor equilibrium solutions. There are essentially three regimes of behavior:

***Regime 1*** (too many starting TD cells): When *χ*_1_ begins above *χ*_1_^*crithigh*^, *χ*_0_ shrinks monotonically to zero, increasing the value of *χ*_1_ as it does so, until *χ*_0_ = 0 (i.e. SC are exhausted). See the red curves in Fig 2B and 2C.
***Regime 2*** (not enough starting TD cells): If *χ*_1_ begins below *χ*_1_^*critlow*^, *χ*_0_ necessarily falls and *χ*_1_ necessarily rises. If *χ*_0_ runs out before *χ*_1_ reaches *χ*_1_^*critlow*^, *χ*_1_’s final state remains below *χ*_1_^*critlow*^. See the blue curves in Fig 2B and 2C. If not, the system enters regime 3.
***Regime 3*** (the “growth-leap” case): If initial *χ*_0_ is large enough to carry *χ*_1_ past *χ*_1_^*critlow*^, positive feedback then drives a rapid increase in net growth rate that pushes *χ*_1_ above *χ*_1_^*crithigh*^ (see the green curves in Fig 2B and 2C, and S2 Fig). The threshold for this “growth-leap” actually requires fewer initial *χ*_1_ cells when there are more *χ*_0_, (Fig 2D). It is only at the limit as initial *χ*_0_ approaches zero that the initial value *χ*_1_ required for a growth leap approaches *χ*_1_^*critlow*^ (indicated by the red vertical plane in Fig 2D).

**Box 1. Final State Analysis of a Two-Stage Lineage with Feedback**

Taking the ODE representation of a two-stage lineage with negligible cell death Eq (2) and representing feedback by defining *P* as presented in the main text, we obtain the following dynamical system.

$$\begin{array}{l} \frac{d\chi_{0}(\tau )}{d\tau }=\left(2p\left(\frac{1}{1+\gamma \;\chi_{1}(\tau )}\right)\left(\frac{\phi \;\chi_{1}(\tau )}{1+\phi \;\chi_{1}(\tau )}\right)-1\right)\chi_{0}(\tau ),\\ \frac{d\chi_{1}(\tau )}{d\tau }=2\left(1-p\left(\frac{1}{1+\gamma \;\chi_{1}(\tau )}\right)\left(\frac{\phi \;\chi_{1}(\tau )}{1+\phi \;\chi_{1}(\tau )}\right)\right)\chi_{0}(\tau ). \end{array}$$

It is easy to see that the accumulation of TD cells (*χ*_1_) is just the time integral of the expansion and contraction of the SC pool, i.e.

$$\chi_{1}\left(t\right)=\int_{0}^{t}2\left(1-p\left(\frac{1}{1+\gamma \;\chi_{1}\left(\tau \right)}\right)\left(\frac{\phi \;\chi_{1}\left(\tau \right)}{1+\phi \;\chi_{1}\left(\tau \right)}\right)\right)\chi_{0}\left(\tau \right)d\tau$$

As *t* →∞, this integral converges only if *χ*_0_ →0, which is guaranteed by the fact that, for sufficiently large *χ*_1_, *χ*_0_’(*τ*) necessarily falls below 0. There are then two interesting regimes to consider: when *χ*_0_’(*τ*) < 0 at all times—this is what occurs if there is only negative feedback on *P*—and when *χ*_0_’(*τ*) spends some time in positive territory, which can occur if positive feedback is sufficiently strong relative to negative.

Although the solution for *χ*_1_(∞) in the above integral equation can only be expressed implicitly (see section 3 of S1 Text), we can still find the values of *χ*_1_ at which *χ*_0_’(*τ*) changes sign. There are two such values, which we refer to as *χ*_1_^*critlow*^ and *χ*_1_^*crithigh*^:

$$\begin{array}{l} \chi_{1}^{critlow}=\frac{-\gamma -\phi +2p\phi -\sqrt{-4\gamma \phi +{\left(\gamma +\phi -2p\phi \right)}^{2}}}{2\gamma \phi },\\ \chi_{1}^{crithigh}=\frac{-\gamma -\phi +2p\phi +\sqrt{-4\gamma \phi +{\left(\gamma +\phi -2p\phi \right)}^{2}}}{2\gamma \phi }. \end{array}$$

Note that these are identical to the non-zero steady states of the two-stage lineage system with TD cell turnover Eq (1); accordingly, for *χ*_1_^*critlow*^ and *χ*_1_^*crithigh*^ to be positive and real, the same lower bound on *ϕ*/*γ* exists as is required for bistability of the steady state system.

Essentially, the qualitative dynamics of the final system can be understood by following the trajectories of *χ*_1_ relative to *χ*_1_^*critlow*^ and *χ*_1_^*crithigh*^ (see section 4 of S1 Text). When either *χ*_1_ > *χ*_1_^*crithigh*^, or 0 < *χ*_1_ < *χ*_1_^*critlow*^, *χ*_0_ proceeds monotonically to extinction (Fig 2C). When *χ*_1_ lies between *χ*_1_^*critlow*^ and *χ*_1_^*crithigh*^, both *χ*_0_ and *χ*_1_ grow (Fig 2B and 2C); this persists until *χ*_1_ breaches *χ*_1_^*crithigh*^, at which point *χ*_0_’(*τ*) switches signs and *χ*_0_ again monotonically shrinks. We refer to the region between *χ*_1_^*critlow*^ and *χ*_1_^*crithigh*^ as the “growth leap” regime, because within it both SC and TD cells undergo transient expansion, whereas outside of it SC pools always contract.

Mechanistically, the abrupt transitions between regimes, as a function of initial conditions, are analogous to the abrupt transitions between steady states in Fig 1, although the precise relationships between initial conditions and final state are not identical. Final-state systems may not properly be called bistable, since they are not attracted to fixed, dynamic stable states. We therefore refer to them as bimodal, in the sense that they may be switched between slow and fast growth modes as function of the sizes of cell populations. Like bistability, bimodality creates an opportunity for ultrasensitive switching. Not only can small differences in initial conditions cause abrupt changes in the growth trajectories of feedback-controlled tissues, as shown in Fig 2D, so can small changes in the strength of either positive or negative feedback (see S3 Fig).

Similarities and differences between the possible behaviors of steady state and final state systems, analyzed over all possible parameter values, and in the face of different kinds of TD-derived feedback, are summarized in Table 1, and Tables A and B in section 5 of S1 Text (see also S4 Fig in which positive feedback arises from SCs). These results show that the presence of positive feedback is required for bimodality, the presence of negative feedback is required for stability, and to achieve either bistability, or bimodality with a stable final state, both positive and negative feedback are required.

**Table 1:** Summary of possible behaviors with feedback when *p* > 0.5. This table summarizes possible steady state and final state behaviors when maximal progenitor self-renewal is greater than 0.5. Without feedback, the open loop system’s output is largely dependent on *p*, the probability that a progenitor cell will divide into an identical cell type instead of a differentiated cell type. Negative feedback eliminates this dependence on *p*, but such systems can only reach a single fixed point in both the steady and final state scenarios. Positive feedback, on the other hand, permits unbounded growth, which would be detrimental to achieving precision in tissue and organ growth. Together, however, lineages can growth to one of two distinct fixed points, which are either bistable (in homeostatic equilibrium) or bi-modal (i.e. stationary and non-dynamic).

*Steady state systems*				
	*Zero steady state*	*Non-zero steady state*	*Unbounded growth*	*Bistability*
No feedback	Never	Never	Always	Never
Negative feedback	Never	Always	Never	Never
Positive feedback	Sometimes	Never	Sometimes	Never
Neg. & Pos. feedback	Sometimes	Sometimes*	Never*	Sometimes*
*Final state systems*				
	*Zero final state*	*Non-zero final state*	*Unbounded growth*	*Bi-modality*
No feedback	Never	Never	Always	Never
Negative feedback	Never	Always	Never	Never
Positive feedback	Never	Sometimes	Sometimes	Always
Neg. & Pos. feedback	Never	Always*	Never*	Sometimes*

*These behaviors are general for some, but not all, feedback configurations, for example a 1/(1 + *ϕ χ*_1_(*τ*) + *γ χ*_1_(*τ*)) configuration changes these outcomes to: “Never,” “Sometimes,” and “Never” for the steady state and “Sometimes,” “Sometimes,” and “Never” for the final state. Alternative ways of combining feedback and their outcomes are summarized in Tables A and B in section 5 of S1 Text.

### More complex lineages

**Fig 3:**
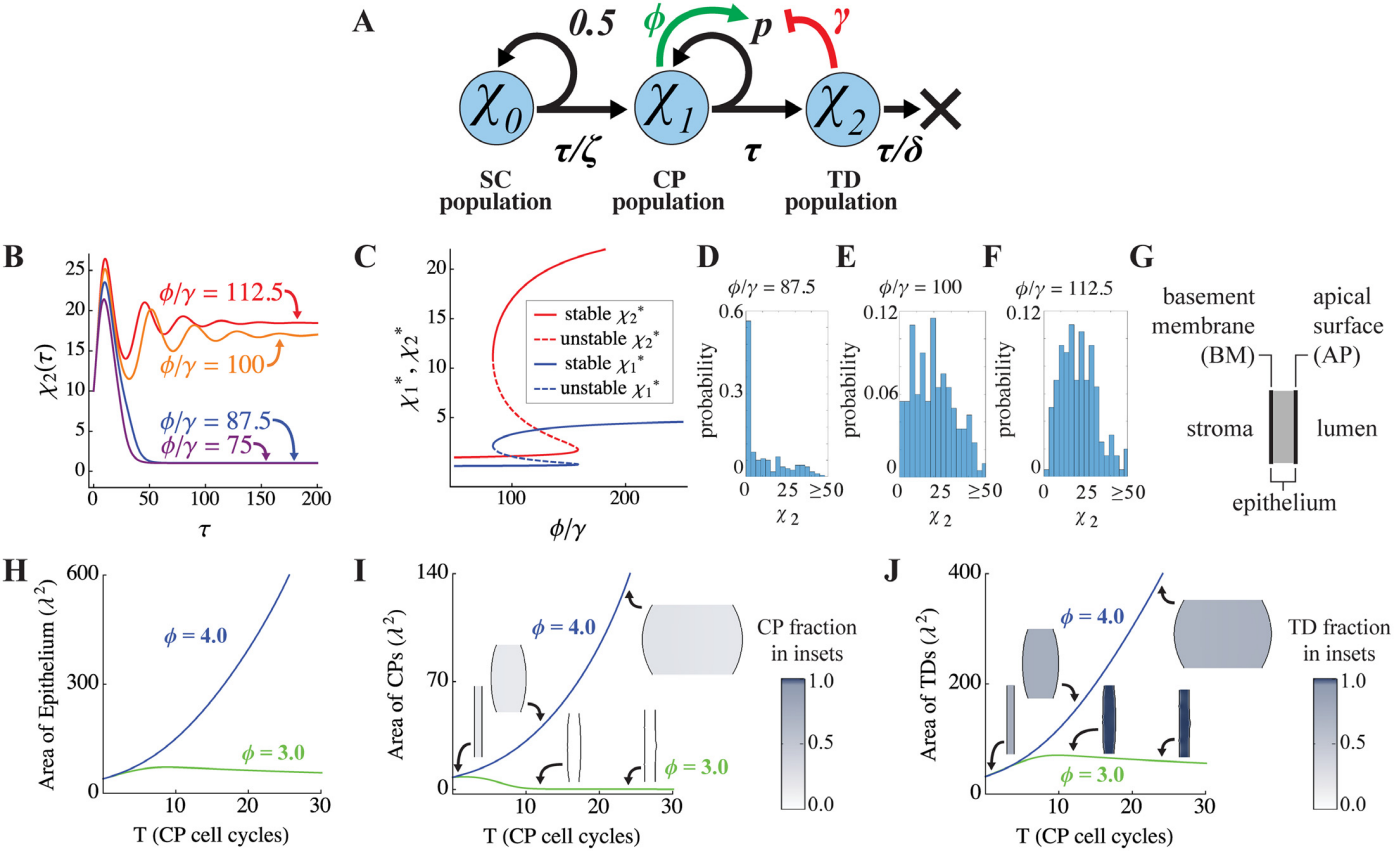
Bistability and spatial bi-modality in a three-stage lineage. **(A)** Here, stem cells (SC) are assumed to maintain a constant output of committed progenitor (CP) cells (the mechanism for which is not modeled explicitly). The self-renewal probability of CP cells is taken to be regulated by positive feedback from CP cells themselves, and negative feedback from terminally differentiated (TD) cells. The parameter *ζ* denotes the division rate of SCs relative to that of the CPs. **(B)** As with two-stage lineages (Fig 1), the equilibrium state is determined by the positive-to-negative feedback ratio, *ϕ*/*γ* (here *ϕ* is varied while *γ* is held fixed; initial conditions are *χ*_0_(0) = 1, *χ*_1_(0) = 4, and *χ*_2_(0) = 10, and *τ* is in units of CP cell cycles). **(C)** CP and TD steady states display bistability as a function of *ϕ*/*γ*. Parameters for B-C are *ζ* = 0.09, *δ* = 0.2, *p* = 0.75, and *γ* = 0.02. **(D-E)** The probability distributions of *χ*_2_ after 200 CP cell cycles, obtained from simulations in which both the self-renewal parameter (*p*), and the initial conditions for *χ*_*0*_, *χ*_*1*_, *χ*_2_ were taken to be normally-distributed random variables with means given by their values in panel C and variances 0.001. These variables were sampled every CP cycle and 200 realizations were performed. The results are shown for three different values of *ϕ*/*γ*, as labeled. Other parameters were as in panel C. **(G-J)** The behavior of the same lineage as in A was simulated in a spatial domain (G), in which the initial geometry simulates that of an epithelium, with SCs confined to a basement membrane (BM). The total areas of the epithelium (H), CPs (I) and TDs (J) are plotted as functions of time (measured in CP cell cycles), for different positive feedback strengths, *ϕ*. *λ* represents the intrinsic decay length of feedback factor G, which is here taken to be about 50 μm. The spatial distributions of CPs and TDs at the start of the simulation, and at the 12^th^ and 24^th^ CP cell cycles, are shown as insets in panels I and J, respectively. As the results show, with positive feedback gain *ϕ* = 3.0, the initial tissue grows exponentially at early times, but growth is not sustained. In contrast, with *ϕ* = 4.0, growth occurs at a similar rate at early times, but continues to expand exponentially at later times. A complete list of parameter values can be found in S1 Table.

Next, we consider a three-stage, steady-state lineage, in which we also alter the source and target of positive feedback. In this particular scenario (Fig 3A), an intermediate, committed progenitor (CP) is the target of all feedback, and a constant influx of progenitor cells from a stem cell source guarantees non-extinction of the CP pool (we assume that the SCs are in equilibrium). Negative feedback from TD cells is now mixed with positive feedback from CP cells themselves. The ODEs for this system are

$$\begin{array}{l} \frac{d\chi_{0}(\tau )}{d\tau }=0\\ \frac{d\chi_{1}(\tau )}{d\tau }=\zeta \;\chi_{0}(\tau )+\left(2P(\tau )-1\right)\chi_{1}(\tau )\\ \frac{d\chi_{2}(\tau )}{d\tau }=2\left(1-P(\tau )\right)\chi_{1}(\tau )-\delta \chi_{2}(\tau ) \end{array}$$ (3)

in which *ζ* is the rate of proliferation of SCs, relative to that of CPs, and *P* = $p\left(\frac{1}{1+\gamma \;\chi_{2}(\tau )}\right)\left(\frac{\phi \;\chi_{1}(\tau )}{1+\phi \;\chi_{1}(\tau )}\right)$. Such an arrangement models, for example, the apparent roles of GDF11 and FGFs in the mouse olfactory epithelium [25].

As observed in the analysis of the two-stage lineage, we see here that the ratio *ϕ*/*γ*, which captures the relative strength of positive versus negative feedback, plays a determining role in the emergence of bistability and ultrasensitivity (Fig 3B), although now both stable states are non-zero (Fig 3B and 3C). Further, when *p* and the initial data are taken to be random variables of the type used in Fig 1H–1J, the distributions of the TD cell population at a given time are similarly dependent on *ϕ*/*γ* (Fig 3D–3F). When *ϕ*/*γ* is small, the system is subject to stochastic extinction events even though the stable steady states are non-zero (Fig 3D). When *ϕ*/*γ* is sufficiently large, the most likely state of the system is the largest stable population, although the distribution of the populations is again fairly broad (Fig 3F). When *ϕ*/*γ* is an intermediate value, the distribution tends to be bimodal with peaks near the two steady states; the probability of stochastic extinctions decreases with increasing *ϕ*/*γ*.

Although the three-stage lineage model is not as analytically tractable as the two-stage model, numerical solutions clearly demonstrate that the system exhibits bistability with respect to feedback parameters and initial conditions (see S5A–S5F Fig). Similarly, growth bimodality is also observed if cell turnover is set to zero, or made very slow (see S5G and S5H Fig).

### Taking account of space

Real tissues are not “well-stirred”, but grow outward into physical space. This potentially influences the feedback regulation of cell lineages in three ways. First, diffusible molecules that mediate feedback will naturally form spatial gradients, so that the concentrations of feedback factors—and thus the effects of feedback—will differ at different locations. Second, growth may displace (advect) both cells and diffusible molecules to potentially different degrees at different locations. Third, boundary conditions (i.e. the disposition of molecules and cells when they reach natural tissue boundaries) may influence both cell behaviors and molecular gradients.

To account for spatial variation, we re-interpret the variables in Eq (2) as representing a local measure of cell concentration, i.e. the volume fraction occupied by each cell type, and add additional equations for the spatial distributions of negative and positive feedback factors, which we refer to as *G* and *F*, respectively. Because the time-evolution of the distributions of these molecules is expected to be much faster than time scales of growth, we assume a quasi-steady state distribution for them. We allow *G* to be produced by TD cells; *F* to be produced by CP cells, TD cells or both; and in some cases also consider the potential effects of external sources of *G* or *F* (e.g., stromal cells). The distribution of *F* is governed by Eq (7) in **Methods;** the equation for *G* is analogous. The forms of the external sources and sinks are given in Eqs (13)–(17) in **Methods**. The effects of *F* and *G* on CP self-renewal are modeled exactly as in Figs 1–3, with parameters *γ* and *ϕ* representing the strengths of negative and positive feedback, respectively (see Eq (6) in **Methods**).

For simplicity, growth and diffusion are simulated here in two-dimensional domains, (extension to three dimensions is straightforward). As an initial condition we typically begin with tissues represented as thin, rectangular domains, as might be encountered in growing epithelia; see the schematic in Fig 3G. We denote one long side as apical [AP], and impose there a reflective (no-flux) boundary condition on molecular diffusion (representative of the tight junctions that exist at the apical surface of most epithelia). On the other side, where one would normally find a basement membrane [BM] that is not itself a barrier to diffusion, we impose no boundary condition, allowing molecules to move unimpeded into the adjacent space, which may be taken to represent the stroma upon which most epithelia sit. In some cases, we also model the presence of molecules in the stroma that act as absorbers, or sinks, for diffusible factors (e.g. follistatin in the stroma beneath GDF11- and activin-expressing epithelia [25, 38]), see Eqs (15) and (16) in **Methods**.

Diffusion, proliferation, differentiation and cell turnover are then modeled together with soft-tissue mechanics (as described in [39, 40]). The dynamics of the epithelial cells are governed by Eqs (4), (5) and (8)–(12) in **Methods**. In addition, the SC population is restricted to the basement membrane (BM) and set to self-renew with probability 1/2. Temporal and spatial trajectories of tissue growth in such models were explored over a wide range of parameter values to identify the sorts of qualitative behaviors that emerge. A complete list of dimensionless parameters can be found in S1 Table in Supplementary Materials. The nondimensionalization is given in section 6 of S1 Text. See Methods for a discussion of the numerical implementation of the governing Eqs (4)–(12) and section 6 of S1 Text for additional model and numerical details.

### Feedback-driven bimodality of growth

As in non-spatial models, spatial systems may be initiated from small size and grow to include large numbers of CP or TD cells. Interestingly, in spatial models the presence of negative feedback control no longer guarantees that a stable steady state, or final-state, will be reached for all parameter values. If the strength of negative feedback is not sufficient, tissue size will increase indefinitely (see Fig 3H, blue curve). This reflects the fact that diffusible signals have limited ranges of action, so that the incremental increase in feedback, averaged over a tissue, that accompanies any incremental increase in tissue size, gets progressively smaller the larger a tissue gets.

This effect notwithstanding, we clearly observe that the mixture of positive and negative feedback can produce growth that is bimodal, switching ultrasensitively between fast and slow (or zero) growth rates as a function of the *ϕ*/*γ* ratio. For example, Fig 3H–3J compare the epithelia dynamics using positive feedback strengths *ϕ* = 3.0 and *ϕ* = 4.0 (holding other parameters fixed; see S1 Table). Note that in this example, initial growth is similar for both feedback strengths, but in the *ϕ* = 3.0 case (green curves and insets), the lack of sufficient positive feedback causes increased differentiation of CP cells into TD cells, elevating the concentration of *G*, which causes CP numbers to decline even further (Fig 3I). The decline in CPs eventually causes TD production to fall to a low level that is insufficient even to balance TD turnover (Fig 3J). In contrast, when *ϕ* = 4.0, the exact opposite sequence of events occurs, and CP levels rise to a value that sustains continual growth (see blue curves and insets in Fig 3H–3J). The distributions of the feedback regulators, the self-renewal fractions and the cells can be found in S6 Fig for *ϕ* = 3.0 and S7 Fig for *ϕ* = 4.0 in Supplementary Materials. Similar results are found when CP cells also produce G (S8 Fig), TD cells also produce F (S9 Fig), the ratio *D*_*F*_/*D*_*G*_ is decreased (S10 Fig), and the sink of G in the stroma is removed (S11 Fig).

Although such systems exhibit a sharp threshold in behavior at a critical value of *ϕ*/*γ*, the value itself is not easily calculated, as it depends not only on initial conditions, but also on the spatial gradients on *G* and *F*, and thereby on the tissue geometry, as well as on the relative locations of cell types (see S12A Fig for a plot of the feedback ratios for the simulations in Fig 3H–3J).

We also explored the effects of stochasticity on the results in this spatial model. In particular, we considered two sources of random fluctuations—the self-renewal parameter *p* and the relative proliferation rate *ζ* of SCs. In S12B Fig, both are taken to be normally-distributed random variables in space and time with means given by their deterministic values. The results indicate that, when the variance is relatively small, simulations evolve similarly to what is seen in the deterministic case. When the variance is large, self-sustaining growth may occur spontaneously even when it would not, under deterministic conditions. When *ϕ* is large enough (e.g. *ϕ* = 4.0), we found that growth may self-sustain even for small variances.

### Exogenous triggering of growth and growth-arrest

**Fig 4:**
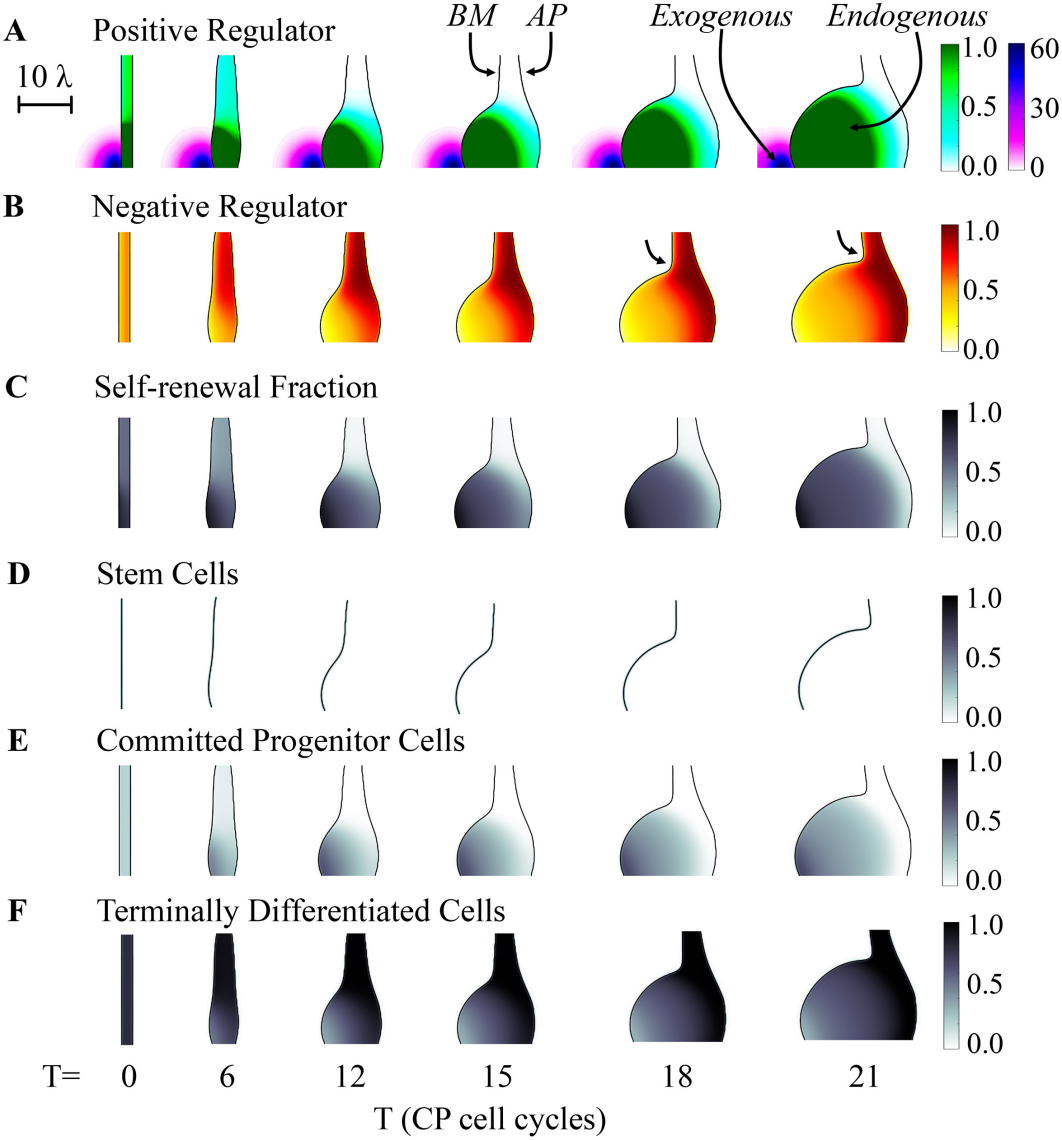
Effect of exogenous application of a graded positive regulator. The spatial development of a three-stage lineage was simulated as in Fig 3, under conditions (*ϕ* = 3.0) that would normally not lead to sustained growth. An exogenous source of “*F*”, the same factor that mediates positive feedback on CP self-renewal is placed near the lower left edge of the initial tissue, leading to sustained growth near the source, and a sharp boundary between rapidly growing and non-growing domains. Colors and shading track the concentrations of *F* and *G* (the negative feedback factor) over time and space (**A-B**), as well as the value of *P*, the CP self renewal fraction (**C**), and the volume fractions occupied by SC, CP and TD cells (**D-F**, respectively). The arrows in B mark the neck of the growing bud. The effect of the exogenous factor is to induce a local increase in the self-renewal fraction of CPs (e.g., see panel C, after the 12^th^ CP cell cycle), which drives local growth of the epithelium, formation of a bud due to the presence of a sharp border between high and low growth states, and a spatial stratification of the CPs and TDs populations and the feedback regulators they produce. The scale bar is in units of the diffusional decay length of the negative feedback factor *G* (*λ*), i.e. approximately 50 μm. See S1 Table for a complete list of parameter values.

One consequence of the dependence of growth trajectories on the spatial distributions of feedback molecules is that localized, exogenous sources of the same molecules can be used to create localized, persistent differences in growth rate. In Fig 4 we consider an epithelial tissue in which the *ϕ*/*γ* ratio would not normally be sufficient to sustain continued growth, but an exogenous point source of *F* is placed near the basement membrane (such a source might represent the production of a growth factor by a group of stromal cells, for example). In response, a local outgrowth appears and continues to grow, producing a stratified arrangement of cells, with CP cell densities highest near the *F*-source and TD cells highest farther away. Notice that the outgrowth becomes sharply demarcated, i.e. there is a distinct notch, or “neck” that separates it from the adjacent epithelium (see arrows in Fig 4B), despite the fact that the exogenous gradient of F declines smoothly and gradually across this zone. Because of this demarcation, we refer to the structure as a “bud”.

**Fig 5:**
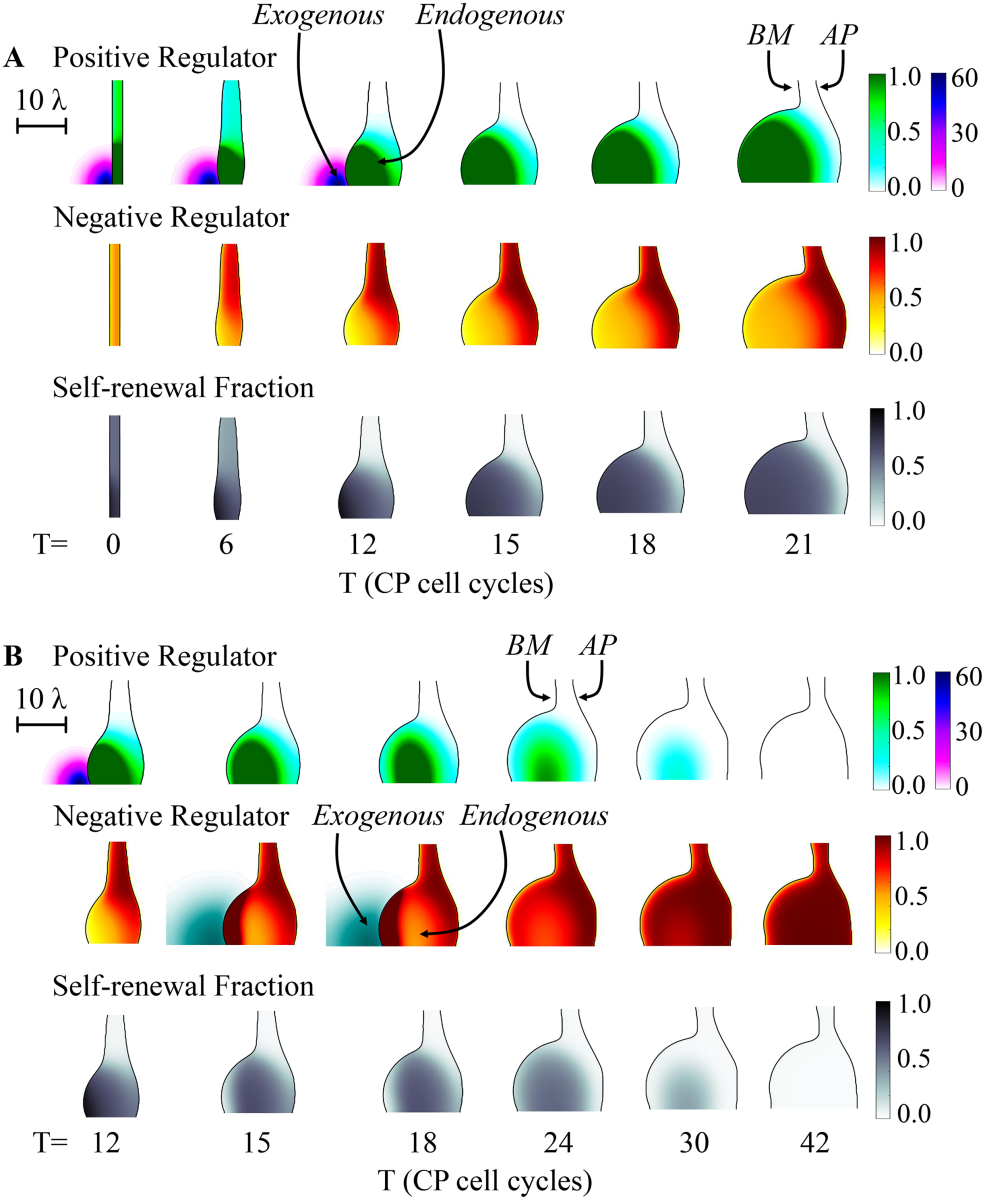
Effect of transient application of positive and negative regulators. **(A)** The spatial development of a three-stage lineage is modeled as in Fig 4, except that the exogenous source of *F* is removed after the 12^th^ CP cell cycle. The spatiotemporal distributions of the positive regulator *F* (exogenous: purple; endogenous: green), the negative regulator *G*, and the CP self-renewal fraction *P* are shown in the three panels. The results show that the transient stimulus is sufficient to drive the CP self-renewal fraction above a critical threshold, beyond which bud growth becomes self-sustaining. **(B)** In this example, the simulation proceeds identically to panel A for the first 15 CP cell cycles, after a localized exogenous source of *G* is added for three cell cycles, and then removed. The result is an abrupt and sustained arrest of growth. Spatiotemporal distributions of *F*, *G* and *P* are plotted as in panel A, with an additional dark green scale added for exogenous *G*. Scale bars in A and B are in units of the diffusional length of feedback factor G (*λ*), i.e. approximately 50 μm. See S1 Table for a complete list of parameter values.

Interestingly, once such a bud is induced, it may sustain its growth independently after the *F*-source is removed. This is shown in Fig 5A, in which the exogenous source is shut off after the 12^th^ cell cycle (see S13 Fig for the corresponding evolution of the cell distributions). In this case, bud growth continues unabated, with an outcome almost identical to that in Fig 4A (see S21 Fig for a comparison). The reason is that the growing bud itself becomes an effective source of *F*, and it is this endogenous feedback that drives most of the growth (see S14 Fig, which shows the distributions of *P*). If exogenous feedback is removed too soon, however, before the bud is large enough, endogenous feedback is insufficient to sustain growth (see S15 and S16 Figs). In particular, CPs differentiate into TD cells, and growth stops.

Just as a transient exogenous positive signal can initiate a self-sustaining process of local epithelial growth, an exogenous source of *G* can also shut off growth. The simulation in Fig 5B begins in the same way as Fig 5A, with a transient source of *F* applied for 12 cell cycles, but following its removal a transient source of *G* is applied from the 15^th^ to 18^th^ cell cycle. Growth of the bud gradually arrests, as CPs differentiate into TDs, and the level of endogenous feedback needed to keep *P* above ½ is no longer sustained. See S17–S20 Figs for plots of the cell distributions, the distributions of *P*, and an analysis of strength of the exogenous source of *G* on the growth arrest. We also tested the effects of stochasticity on *p* and *ζ* as above, and found it more difficult to trigger growth arrest using a transient exogenous source of negative feedback in the stochastic case, i.e. larger amounts of *G* were required to stop growth.

**Fig 6:**
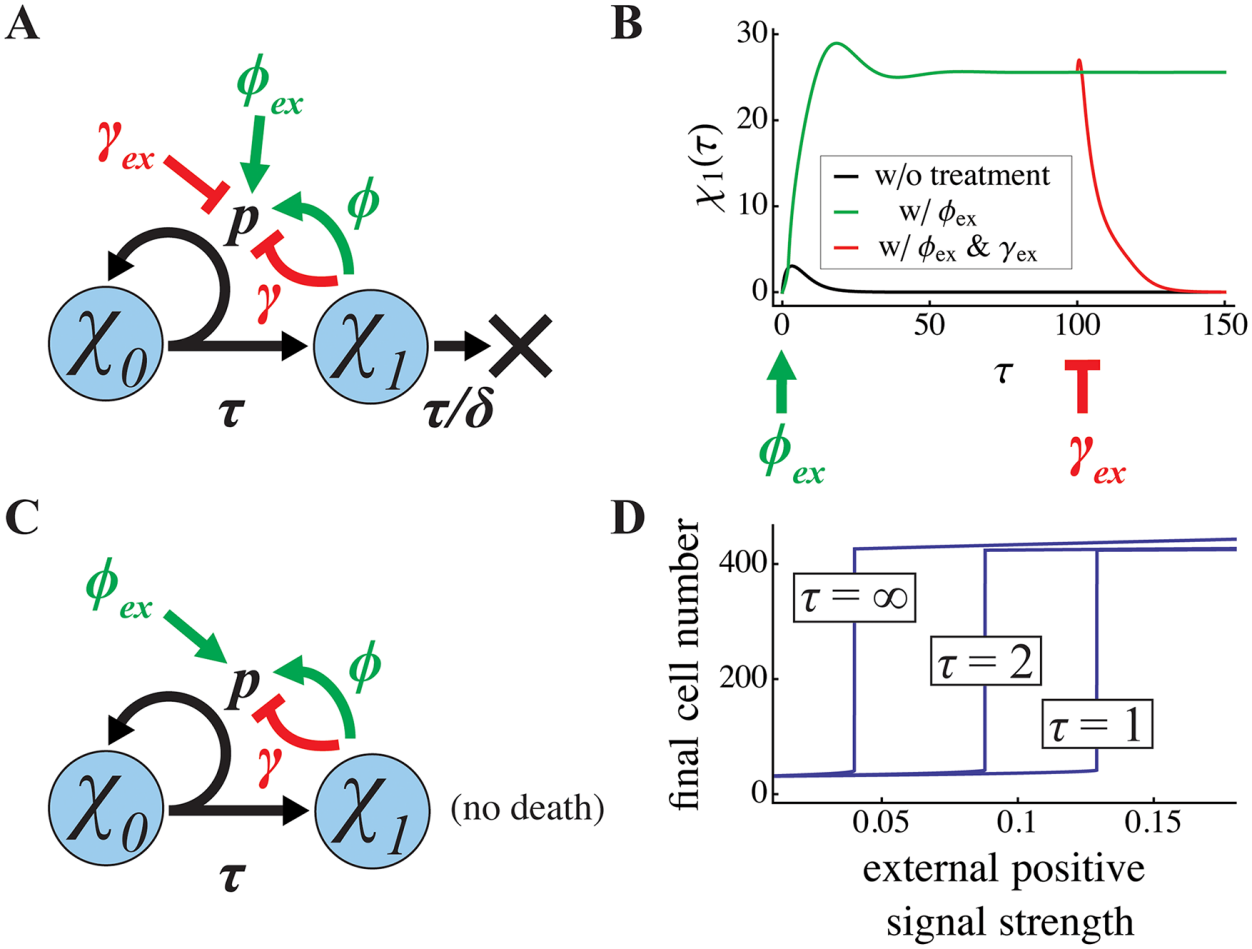
Ultrasensitive switching using exogenous regulators of renewal. To explore the mechanism by which transient exogenous signals trigger or suppress high growth states (Fig 5), the effect of exogenous inputs was studied in the simpler, non-spatial, two-stage lineages of Figs 1 and 2. For a steady state system (**A**), with parameter values that would ordinarily lead to lineage extinction, panel **B** shows the effects of transient exogenous application, for two cell cycles, of a high level (*ϕ*_*ex*_ = 200) of the same renewal-promoting factor that is normally produced by TD cells, followed after the 100^th^ cell cycle by the application, for two cell cycles of a high level (*γ*_*ex*_ = 200) of the same renewal-suppressing feedback factor that is normally produced by TD cells. The first treatment shifts the system to a sustained high growth state, while the second treatment shifts it to a zero-growth state. Parameter values are *δ* = 0.2, *p* = 0.8, *ϕ* = 0.25 and *γ* = 0.015. For a final state system **(C)**, in which cell death is negligible, panel **D** displays the effects on the final state of providing a pulse of various durations (numbers of cell cycles, *τ*) of an exogenous, renewal-promoting factor. Although the minimum level of exogenous factor required to trigger the high growth state is a function of pulse duration, the value of the final state that results once a high growth state is triggered is remarkably similar for pulses of all magnitudes and durations. Parameter values are *p* = 0.8, *ϕ* = 0.05, and *γ* = 0.002.

These sorts of results (which are summarized in S21 Fig) are reminiscent of the observation in non-spatial systems that a single system can be moved in or out of the “growth-leap” region simply through an appropriate change in initial conditions (Fig 2D). In Fig 5A, the exogenous source of *F* drives growth of a localized region of the tissue to a point where it has sufficient endogenous *F* to enter a growth-leap regime. Indeed, as shown in Fig 6, short pulses of exogenous sources of *F* or *G* can also be used in non-spatial simulations to toggle, in an analogous manner, between fast- and slow-growing states. In both cases, it is the inherent bistability/bimodality of the systems that ensures an ultrasensitive dependence of such toggling on the levels of *F* and *G* applied. In spatial systems (Figs 4 and 5), that ultrasensitivity plays out as a sharp demarcation in the region of the tissue that responds to an exogenous cue, even if that cue forms a shallow spatial gradient over a larger region. Spatially demarcated behavior persists even after removal of an exogenous source because endogenous factors also form gradients, and the responses to them are similarly ultrasensitive.

### From buds to self-organizing fingers

**Fig 7:**
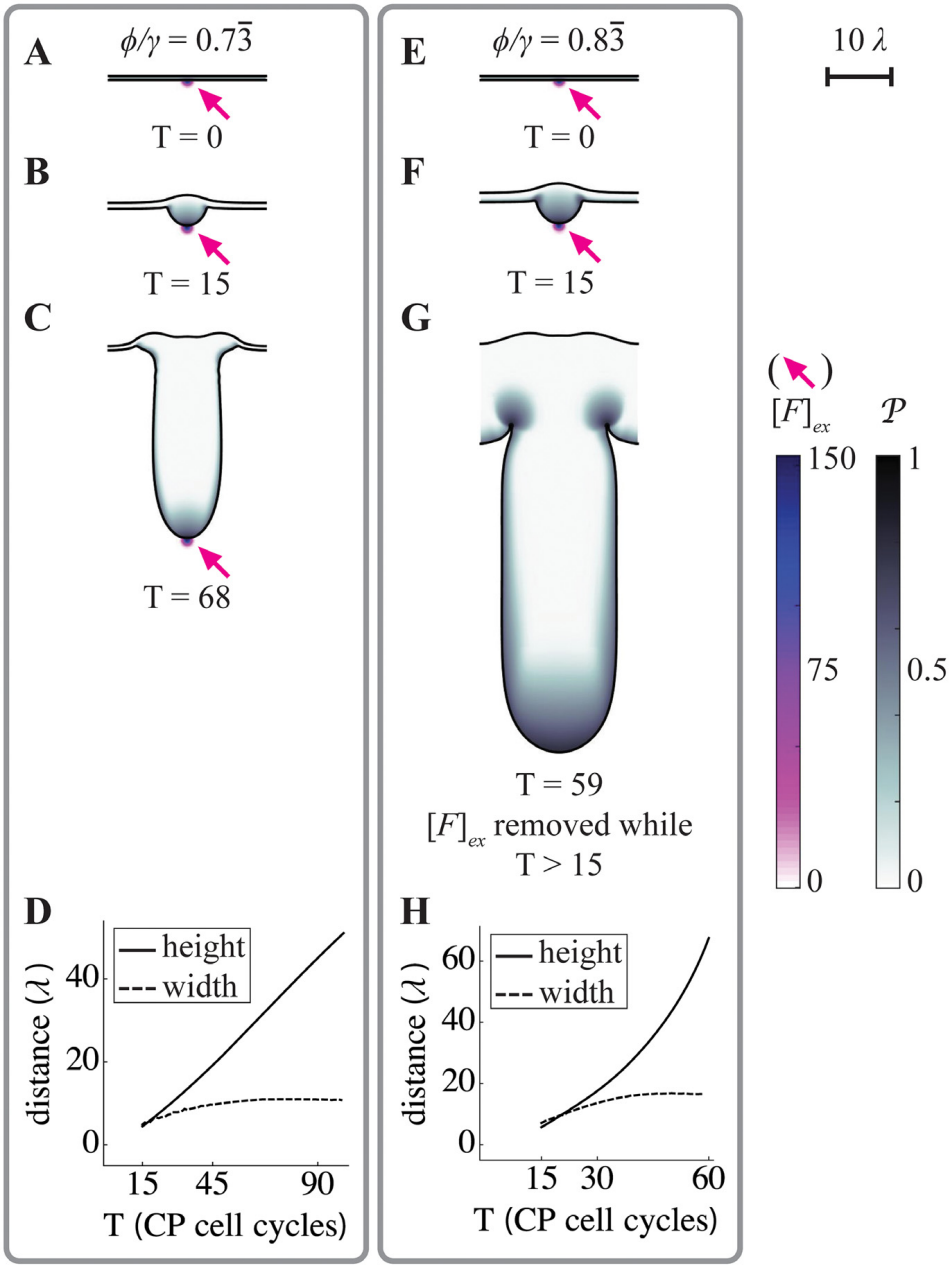
Formation of growing, constant-width fingers. Spatial simulations similar to those in Figs 3–5 were carried out, using a larger domain size, slower diffusivity of feedback factors *F* and *G*, and incorporating strong uptake of G on the stromal side of the epithelium (for parameters see S1 Table; the characteristic decay lengths of *F* and *G*, denoted *λ*, were identical, and can be appreciated from the 10*λ* scale bar in the upper right corner). Boundary conditions were also taken to be periodic. Simulations all began from the same initial flat geometry, with an exogenous source of *F* in the center of the domain (*F*_ex_, arrows), but using different values of the endogenous feedback ratio (*ϕ*/*γ*), as shown. **A-C.** At the lower *ϕ*/*γ* ratio, a fingerlike structure elongates as long as the exogenous signal is present. Values of *P* are high primarily near the source of *F*. **D.** The length of the finger in A-C increases linearly in time, but after initial growth, its width remains constant. **E-G.** At the higher *ϕ*/*γ* ratio, a similar-looking structure is generated, but it continues to grow even after the exogenous source of *F* is removed after the 15^th^ CP cell cycle. Values of *P* remain high primarily near the tip of the growing finger. **H.** Although the finger elongates at an approximately exponential rate, its width also stabilizes at a constant value. Distances in D and H are plotted in units of the intrinsic decay length of both *F* and *G* within the tissue (which is the square root of the ratio of diffusivity to the rate constant of uptake).

**Fig 8:**
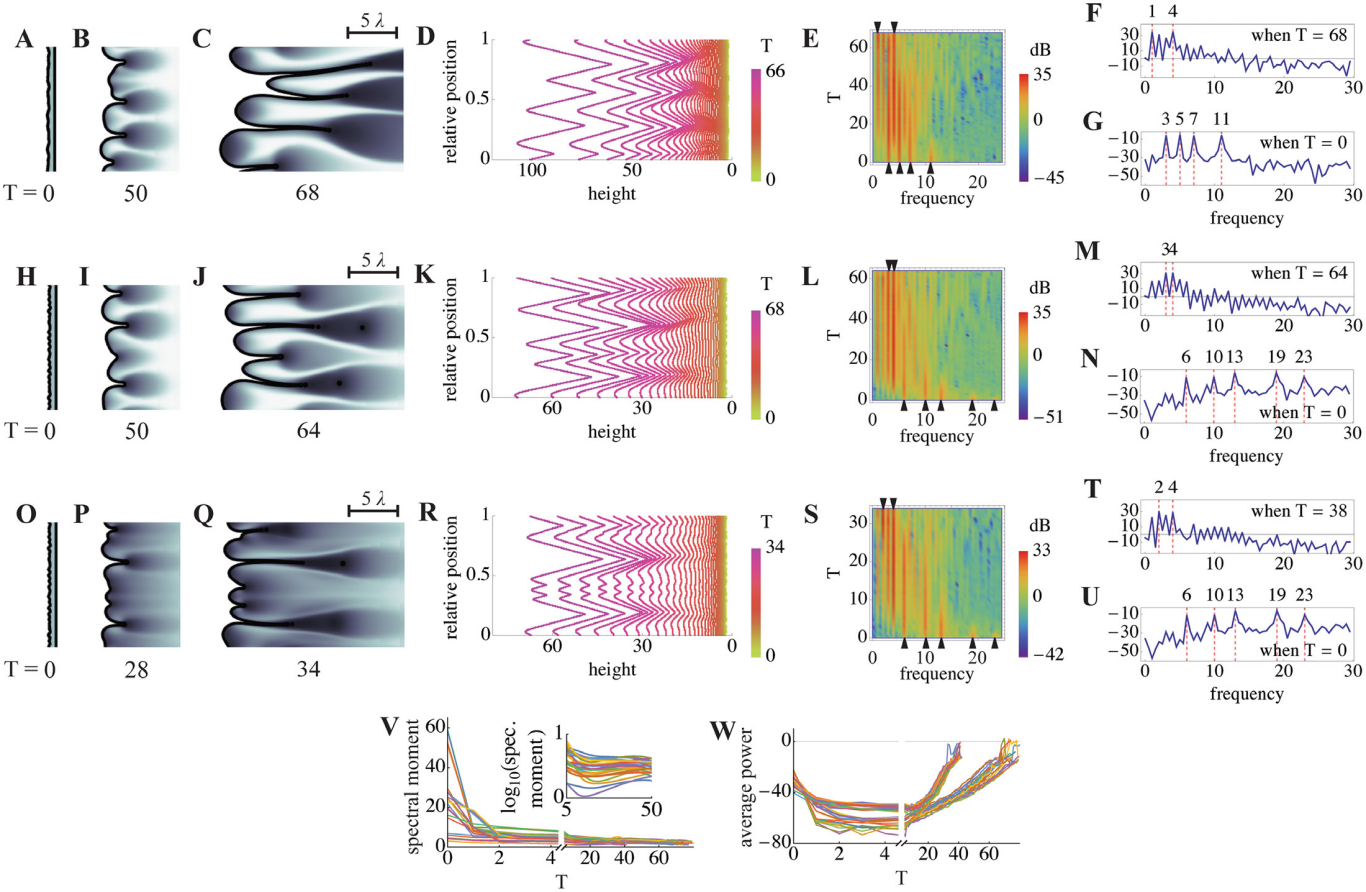
Self-organizing pattern. **A-U.** Simulations were carried out as in Fig 7, except that no exogenous source of *F* was provided, higher values of the *ϕ*/*γ* ratio were used, and the initial shape of the BM was perturbed by the addition of a small amount of “noise”, creating a slightly rough contour (see section 7 of S1 Text for details). Under such conditions, small inhomogeneities in levels of endogenous *F* and *G* near the BM lead to spontaneous and sustained finger growth. In the first two rows (panels A-G and H-N), *ϕ* = 3.0 and *γ* = 5.0 (thus *ϕ*/*γ* = 0.6), but different noisy initial conditions were used. The third row (O-U) starts from the same initial conditions as H-N, but with *ϕ* = 3.5 and *γ* = 5.0 (thus *ϕ*/*γ* = 0.7). Panels A-C, H-K and O-Q show the progress of finger growth, shaded as in Fig 7, at the indicated times (numbers of CP cell cycles). The scale bar shows five times the characteristic decay lengths of *F* and *G*. To facilitate quantitative analysis of these simulations, BM heights were measured as a function of relative position along the BM contour arc length. In panels D, K and R, these measurements are plotted for every other cell cycle from the start to the end of the simulation. Fourier transforms were used to identify the power spectra (power as a function of spatial frequency) of these graphs, and the evolution of such spectra over time was summarized in the form of kymographs, shown in E, L and S, in which intensity at different frequencies is displayed via a heat map, and time is on the ordinate axis. Panels F,G,M,N,T, and U are periodograms—essentially excerpts from these kymographs at single time points—at the end (F, M, T) or beginning (G, N, U) of the simulations, with power displayed on the ordinate axis (units of dB). In all cases, the results show that dominant frequencies present in the noisy initial conditions (arrowheads at the bottoms of kymographs E, L and S) quickly become replaced by lower frequency components (arrowheads at the top of kymographs E, L and S; dominant frequencies are also shown as dashed red lines in F-G, M-N and T-U). **V-W**. Results from the three cases presented in A-U and 21 additional independent simulations, initiated from a variety of noisy initial conditions with a range of initial frequency components and *ϕ*/*γ* values (see section 7 of S1 Text), were analyzed by plotting spectral moment (V) and average power across all frequencies (W), as a function of time (spectral moment is an aggregate measurement that captures the frequency at which power is “centered”). All simulations show a similar pattern: during the first two CP cell cycles, average power declines as most of the noise in the initial conditions is averaged away (in the inset in V, a logarithmic axis is used to show the pruning of both low and high frequencies; *ϕ*/*γ* = 0.6 for these cases). Afterwards, power centered on a low frequency band increases continually, reflecting the elongation of fingers of relatively constant width. The subset of cases in panel W in which power grows more rapidly are those for which *ϕ*/*γ* = 0.7, as opposed to 0.6. See S1 Table for a complete list of parameter values.

The sorts of buds that appear in the simulations in Figs 4 and 5 are simple, expanding balloon-like structures, with relatively little spatial organization. One reason for this is that the domains being simulated are relatively small compared with the ranges over which diffusible factors spread. To explore whether more intricate structures can be formed, it is necessary to “zoom out”, either by increasing the size of the simulated domain or—equivalently—reducing the spread of the diffusing molecules. In Figs 7 and 8 we do the latter, decreasing the intrinsic decay lengths of *F* and *G*, and adding uptake of the negative feedback factor *G* in the stroma (equivalent to including stromal expression of an irreversible inhibitor, such as follistatin [25, 38]). In Fig 7, we start with initial conditions from which growth does not spontaneously occur, and initiate growth through a point source of *F* (Fig 7A and 7E). In panels A-C the *ϕ*/*γ* ratio is set at a level that does not allow for self-sustaining growth, and therefore the exogenous source is left on throughout the simulation (as in Fig 4). In panels E-G, the *ϕ*/*γ* ratio is slightly higher, so that growth, once initiated, will self-sustain, and thus the exogenous source is removed after 15 cell cycles (for further details, see S22 and S23 Figs, which show the distributions of the cells and *F* and *G*). In both cases, buds initiate, but they continue to grow outward as fingers of relatively constant width (Fig 7D and 7H). Interestingly, this width is at least 10 times the characteristic decay lengths of either *F* or *G*, implying that it is determined by characteristics of the system other than the spatial ranges of action of diffusible signals.

The ability of a self-sustaining finger to grow in one direction, while maintaining a constant width in the other, suggests some sort of spontaneous self-organizing ability. One way to test for spatial self-organization is to see whether noise (random small inputs) gets transformed into quasi-regular patterns that are independent of the details of the noise. For example, reaction-diffusion systems that generate Turing patterns, when initiated from any of a variety of noisy initial conditions, evolve to produce patterns of stripes or spots with characteristics that directly reflect the underlying parameters of reaction and diffusion.

In our simulations, a simple way to mimic noisy initial conditions is to make many small perturbations to the initial shape of the modeled tissue. In Fig 8, small, sinusoidal perturbations of a variety of arbitrary spatial frequencies were introduced along the basal surface. Values of the *ϕ*/*γ* ratio were chosen so that self-sustaining growth would not normally occur. However, the shape irregularities introduced by the initial conditions create tiny locales in which endogenous *F* can rise above or fall below the average. Some of these reach the threshold for locally self-sustaining growth, with the result that a series of buds begin to emerge. Eventually, buds fuse to produce wide fingers with a relatively regular spacing. Fingers may continue to fuse, or branch to create new fingers, depending upon parameter values. Fig 8A–8C, 8H–8J and 8O–8Q show the epithelia morphologies together with the distributions of *P* for different ratios of *ϕ*/*γ* and initial conditions. The corresponding distributions of *F* (from Fig 8A–8C and 8H–8J) are shown in S24A and S24B Fig.

As in Fig 7, fingers are many times wider than the characteristic decay lengths of factors *F* and *G* (see scale bar, upper right). We find that finger widths are most readily influenced by changes to the diffusion coefficients *D*_*F*_ and *D*_*G*_, with the widths increasing as a function of *D*_*F*_ and decreasing as a function of D_G_ (see S24C Fig). If *D*_*F*_ gets too large, however, growth fails to self-sustain, because peak levels of *F* become too small.

To show that these patterns are truly self-organized, and do not merely represent the expansion of pattern already present in the initial conditions, we used Fourier transforms of contours extracted from the images in Fig 8D, 8K and 8R to follow spatial frequency components over time. As shown in the kymographs in Fig 8E, 8L and 8S, as well as in individual periodograms excerpted from them (Fig 8F, 8G, 8M, 8N, 8T and 8U), simulations initially display power at frequencies corresponding to those used in the (sinusoidal) initial conditions, but quickly redistribute power to frequencies in the range of 2–4 (i.e. periods of 1/2 to 1/4 of the width of the modeled domain, which in these cases is ~12.6 times the characteristic decay length of diffusible factors *F* and *G*). This occurs regardless of whether the dominant frequencies in the initial conditions are high or low (Fig 8V and 8W; see also S24D Fig), and are independent of both the exact value of *ϕ*/*γ* and the details of the boundary conditions. What these data indicate is that the role of initial conditions in these simulations is merely to get growth started; afterward, spatial interactions organize the behavior of the system so that fingers of similar size are produced. Such self-organization, through spatial interaction, is reminiscent of the evolution of Turing patterns. [21, 22, 41, 42]. As with Turing patterns, this behavior depends upon diffusible, antagonistic factors (“activators” and “inhibitors”). Here, however, the patterns emerge as a spatial manifestation of bistability and bimodality generated by the diffusible factors and cell displacements. Thus, unlike Turing patterns, there is no need for the diffusivities of the factors to be different—indeed the diffusivities of the positive and negative factors are equal in Figs 7 and 8, and the characteristic scales of the patterns are much larger than the characteristic decay lengths of these factors.

## Discussion

Here we show that feedback regulation of stem or progenitor cell self-renewal can lead to growth bistability or bimodality, with ultrasensitive switching between high and low (or zero) growth states. A necessary condition for such behavior is the simultaneous presence of saturable positive and negative feedback. Exactly which lineage stage serves as the source of feedback signals appears less important than the fact that the strength of positive feedback must significantly exceed that of negative. When this type of arrangement is implemented in space, the resulting ultrasensitivity can lead to sharply demarcated growth differences that may be self-sustaining, producing bulges, buds or fingers that could serve as building blocks for morphogenesis.

Such behavior typifies self-organization, in that the forms and sizes of buds and fingers that emerge reflect the parameters and structure of the feedback system, not the details of the stimuli that initiated them. Because we only explored here a small number of very simple lineage architectures, boundary conditions, and sources of noise; and we considered only a limited number of mechanical constraints on cell behavior, it is possible that the diversity of forms that could be generated through lineage feedback is much greater than shown in this study. Even for the relatively simple buds and fingers that we observe here, it will be difficult to determine precisely what system characteristics are the primary determinants of shape and size. This is because the decision by cells whether or not to enter a “growth leap” regime at any point in time and space will be influenced not only by the parameters of the system, but also by the gradients of diffusible factors that are set up by dividing and differentiated cells nearby.

So far, the idea that lineage negative feedback (i.e. negative feedback regulation of self-renewal) plays a major physiological role has come mainly from studies of size control in self-renewing tissues, although the molecular nature of the relevant feedback factors is known in only a subset of such cases [25, 27, 28, 30, 43]. In many of these tissues, positive feedback factors—secreted molecules that promote cell renewal—have been clearly identified, yet there has been little speculation about the purpose of such feedback. Here we raise the possibility that positive feedback is useful because it creates the potential for growth-driven, self-organizing morphogenesis.

In support of this view, we note several *in vivo* reports of phenomena analogous to those discussed here, including growth bistability/bimodality, feedback ultrasensitivity, and feedback-regulated morphogenesis. For example, in the mouse olfactory epithelium (OE), a reduction in the strength of FGF signaling (due to loss of *Fgf8* [44]), or an increase in the strength of GDF11/activin signaling (due to loss of *Foxg1*, which regulates responsiveness to GDF11 and activin [38]), can lead not just to a smaller OE, but to a complete absence of tissue (agenesis). Agenesis due to loss of *Foxg1* can be rescued by inactivation of *Gdf11*, consistent with idea that a critical threshold ratio of positive-to-negative feedback determines whether a zero or non-zero growth state is achieved [38]. Interestingly, inactivation of even a single allele of *Gdf11* in *Foxg1* mutant animals restores the OE to full thickness, supporting the idea that growth state is an ultrasensitive function of feedback strength, as in Fig 1F.

In mouse muscle, evidence for growth bistability can be found in transplantation experiments in which myofiber-associated satellite cells are allowed to re-populate injured muscle [45]. In such experiments the transplanted cells, which are FGF2-treated prior to transplantation, trigger an abnormally high rate of myoblast proliferation and differentiation, which becomes sustained, without further intervention, for years. As in the OE, muscle cell lineages are known to be controlled by mixed negative and positive feedback through secreted factors myostatin [28] and FGFs [46].

Triggered high rates of proliferation and differentiation that self-sustain for long periods are also observed in pathological skin conditions, such as psoriasis, acanthosis nigricans, and keloid formation [47–49]. Psoriasis is a particularly interesting example, because the excessive keratinocyte stem cell proliferation that characterizes psoriatic lesions typically occurs in domains that are sharply juxtaposed with normal skin, for reasons that are not yet understood. Ultrasensitivity that arises from a mix of intrinsic positive and negative lineage feedback could easily account for such sharply demarcated growth differences, as illustrated in Figs 4 and 5. Interestingly, therapeutic resolution of psoriatic lesions typically occurs only when stem cells are ablated below some threshold level [50], exactly what the lineage models presented here predict for a population that becomes “stuck” in a high growth state due to endogenous positive feedback.

In acanthosis nigricans, which often occurs as a consequence of type II diabetes [48, 51], the epidermis thickens due to the presence of deeply folded stem cell layers, producing structures much like those observed in Fig 8. It is thought that high insulin levels interact with insulin-like growth factor-1 receptors to inhibit epidermal differentiation, i.e. insulin acts as an exogenous signal that boosts *P* [52]. In Figs 4–8, just such a signal can clearly act as a trigger of budding and fingering. Of course, fingered growth also occurs in normal tissues, as exemplified by the palisades of Vogt of the corneal limbus; lingual papillae, hair follicles, and even intestinal crypts, which initially develop as downgrowths from a smooth epithelial lining [53–56]. The roles played by diffusible positive or negative factors in the morphogenesis of these structures are incompletely understood, although in several cases the existence of both positive and negative feedback has been documented [55, 57].

Although these examples support the view that interesting, spatially dynamic phenomena do arise *in vivo* as a result of mixed positive and negative lineage feedback, the extent to which the morphogenesis that occurs during normal development relies upon such phenomena remains to be investigated. Some developmental phenomena do bear an intriguing superficial resemblance to observations made here. For example, vertebrate tailbud elongation proceeds through outgrowth, at a relatively constant rate, of a constant-width, finger-like projection, with self-renewing cells concentrated at the tip [58, 59], much like what is observed in Fig 7. Moreover, tailbud outgrowth is known to be driven by high concentrations, at the growing tip, of renewal-promoting FGF [60, 61], and terminated by differentiation-promoting retinoic acid, a diffusible factor that is both exogenously and endogenously provided, and acts in an ultrasensitive fashion to extinguish growth [61].

Obviously, feedback regulation of lineage progression is only one of many morphogenetic mechanisms at the disposal of developing tissues. Processes such as regulated cell cycle progression, directed cell migration, cell sorting, and active mechanical deformation clearly play important roles as well [e.g. 7, 17–20, 62–64]. What the present study elucidates are some of the advantages of lineage feedback-driven morphogenesis. These include the ability to specify spatial structure using the same molecules that regulate tissue homeostasis; the ability to coordinate the behaviors of cells over reasonably large spatial ranges; and the ability to create structure through self-organization, i.e. to form borders and boundaries spontaneously without the need for templates or pre-patterns.

## Methods

### Steady state, final state, and dynamics

Analyses of the spatially homogeneous, ordinary differential equation models were performed using Wolfram Mathematica. Simulations of these models were performed using NDSolve. Stochastic simulations were carried out using MATLAB.

### Multispecies interactions

The volume fractions of CP (*χ*_1_) and TD (*χ*_2_) satisfy the mass conservation equation

$$\frac{\partial \chi_{i}}{\partial t}=-\nabla \cdot J_{\chi_{i}}+Src_{\chi_{i}}-\nabla \cdot (u\chi_{i}),$$ (4)

where *i* = 1,2. The flux **J**_*χi*_ accounts for interactions among cell species, and **u** is the mass-averaged cell velocity. Here we have assumed that the cell densities are matched, that the cells are tightly packed and that the cells move with the same velocity following [39].

The source term *Src*_*χi*_ quantifies the net effects of the proliferation, differentiation and death of the lineage components:

$$Src_{\chi_{1}}=v_{0}\chi_{0}+v_{1}(2p_{1}-1)\chi_{1},Src_{\chi_{2}}=2v_{1}(1-p_{1})\chi_{1}-d\chi_{2}$$ (5)

The self-renewal fraction of the CPs, *p*_1_, is regulated by a soluble negative feedback factor *G* (e.g., GDF) and a positive feedback factor *F* (e.g., FGF) instead of directly by the CP and TD populations in the spatially homogeneous model. Defining [*G*] and [*F*] as the concentrations of *G* and *F* respectively, we take

$$p_{1}=p_{1,\text{min}}+\left(p_{1,\text{max}}-p_{1,\text{min}}\right)\frac{\varphi [F]}{1+\varphi [F]}\frac{1}{1+\gamma [G]},$$ (6)

which is analogous to the feedback in the ODE model. Here *P*_1,min_,*P*_1,max_ are the minimum and maximum levels of self-renewal, and *ϕ*, *γ* are the positive and negative feedback gains respectively.

The concentration [*F*] satisfies the quasi-steady reaction-diffusion equation

$$0=\nabla \cdot \left(D_{F}\nabla [F]\right)+s_{F}^{1}\chi_{1}+s_{F}^{2}\chi_{2}-\left(d_{F}+u_{F}\chi_{1}\right)[F]+ExtSrc_{F}$$ (7)

The equation for [*G*] is identical, but with different coefficients. We use a quasi-steady equation here because the diffusion time scales of *G* and *F* (e.g. minutes) are much faster than cell proliferation time scale (e.g. hours). Since it is not known which cell types produce*F*, the production of *F* may come from CPs or TDs or both. In particular, $s_{F}^{1}$ and $s_{F}^{2}$ are the production rates from CPs (*χ*_1_) and TDs (*χ*_2_) respectively, *d*_*F*_ is the natural decay rate, *u*_*F*_ is the uptake rate by CPs and *D*_*F*_ is the diffusion coefficient. The last term represents the effects of an exogenous source. In the equation for [*G*] *EXTSrc*_G_ also includes a sink that models the binding of *G* by Follistatin (FST) [65]. A complete list of dimensionless parameters and their values may be found in S1 Table.

### Soft tissue mechanics

Following [39, 66, 67], we use a simplified model of soft tissue mechanics to obtain the cell velocity and flux. In particular, we use a generalized Darcy law to describe the passive motion of cells due to proliferation-induced pressure. This assumes that the tissue can be treated as a viscous, inertia-less fluid and also models flow through a porous media. Other constitutive laws and model formulations may be found in [39, 66–72] and are expected to yield similar results. We take the cell velocity to be

$$u=-\kappa \left(\nabla p-\frac{\lambda \;}{\varepsilon }\mu \nabla \chi_{T}\right),$$ (8)

where *κ* is a non-negative cell motility, *p* is the pressure generated by cell proliferation, *μ* is the chemical potential defined as

$$\mu =\left(\frac{df}{d\chi }\left(\chi_{T}\right)-\varepsilon^{2}\nabla^{2}\chi_{T}\right)$$ (9)

where *ε* measures the thickness of the basement membrane and apical surfaces (both assumed to have the same thickness for simplicity). The parameter *λ* models the surface tension (stiffness) of the epithelia interfaces. The surface tension may be different for the BM and AP surfaces. The function *χ*_*T*_ denotes the epithelium volume fraction:*χ*_*T*_ = *χ*_1_ + *χ*_2_, and $f\left(\chi \right)=\frac{1}{4}\chi^{2}{\left(1-\chi \right)}^{2}$ is a double-well potential. Note that the term $\frac{\lambda \mu }{\varepsilon }\nabla \chi_{T}\approx \lambda_{BM}H_{BM}\delta_{\Sigma_{BM}}+\lambda_{AP}H_{AP}\delta_{\Sigma_{AP}}$ where *λ*_*BM*_,*λ*_*AP*_ are the surface tensions of the BM and AP surfaces, *H*_*BM*_,*H*_*AP*_ are the corresponding mean curvatures of the surfaces and $\delta_{\Sigma_{BM}},\;\delta_{\Sigma_{AP}}$ are surface delta functions localized on the basement membrane and apical surfaces, e.g., see [39]. Both terms in Eq (8) model passive cell motion with the first due to differential proliferation and the second due to cell-cell adhesion. Finally, the flux **J**_*χ*_ is modeled as a generalized Fick’s law [39]

$$J_{\chi }=-M\chi \nabla \mu ,$$ (10)

where *M* represents the magnitude of the flux. Together, these constitutive relations for the cell velocity and fluxes guarantee that the model is thermodynamically consistent. That is, the adhesion energy $E_{adhesion}=\frac{1}{\varepsilon }\int \lambda \;\left(f\left(\chi_{T}\right)+\frac{\varepsilon^{2}}{2}{\left|\nabla \chi_{T}\right|}^{2}\right)dx\approx \lambda_{BM}L_{BM}+\lambda_{AP}L_{AP}$, where *L*_*BM*_,*L*_*AP*_ are the lengths of the BM and AP surfaces, is non-increasing in the absence of net sources, where the integration is taken over a domain containing the tissue, see [39].

To solve for the pressure, we assume that the mass conservation equation also holds for the volume fractions of the stroma (*χ*_*s*_) and the fluid domain (*χ*_*L*_) exterior to the AP surface. Defining *χ*_*NE*_ = *χ*_*s*_+*χ*_*L*_, we assume 1 = *χ*_*T*_+*χ*_*NE*_ and correspondingly that $J_{\chi_{NT}}=J_{\chi_{S}}+J_{\chi_{L}}=-\left(J_{\chi_{0}}+J_{\chi_{1}}+J_{\chi_{2}}\right)$. We also assume that there are no net sources in the stroma or fluid domains, i.e. *Src*_*χs*_ = *Src*_*χL*_ = 0. Then, summing the volume fraction equations for the lineage components, the stroma and the fluid, we obtain

$$\nabla \cdot u=Src_{\chi_{0}}+Src_{\chi_{1}}+Src_{\chi_{2}}=Src_{\chi_{T}},$$ (11)

from which it follows that

$$-\nabla \cdot \left(\kappa \nabla p\right)=Src_{\chi_{T}}-\nabla \cdot \left(\kappa \frac{\lambda }{\varepsilon }\mu \nabla \chi_{T}\right)$$ (12)

### Numerical implementation

We solve the system of equations in a rectangular computational domain Ω = {(*x*,*y*)|−20 ≤ *x* 20,−10 ≤ *y* ≤ 10} that contains the epithelial tissue. We use homogeneous Neumann boundary condition for cell types $\chi_{1},\;\chi_{2}$ and feedback factors [*F*],[*G*]. In order to allow the tissue to leave the computational domain smoothly, we apply homogeneous Dirichlet boundary conditions for the pressure *p* and chemical potential *μ*, following [39]. The no-flux boundary condition at the AP is implemented by the diffuse domain approach (see section 6 of S1 Text). The stem cell population $\chi_{0}\approx A(t)\delta_{\Sigma_{BM}}$, where *A*(*t*) is chosen to ensure that numbers of SCs are maintained, even if the BM changes shape. The details about this approach are also found in section 6 of S1 Text.

To solve the governing equations efficiently, we follow previous work in [66] and use an adaptive finite-difference method with an implicit time discretization. The equations at the implicit time step are solved using the nonlinear multigrid method, where the equations are reformulated as a system of second order equations. Additional details may be found in section 6 of S1 Text. The spatial distributions of cells and feedback factors are visualized using Matlab.

### Exogenous source for feedback factors *G* and *F*

Let *T* represent the current time in the simulation. Suppose that the exogenous source for *F* is introduced from time *T*_*F1*_ and *T*_*F2*_. Let

$$ExtSrc_{F}=\alpha_{F}\left(A_{F}-[F]\right)\chi_{S}$$ (13)

for *T*_*F1*_≤*T*≤T_F2_. Here $\chi_{S}$ is the volume fraction of the stroma,$A_{F}$ is a prescribed Gaussian source centered at the lower left corner of the computational region. *F* is produced in the stroma at a rate proportional to its difference from a target concentration *A*_*F*_ with a proportionality constant *α*_*F*_. Given the computational domain {(*x*,*y*)|−20 ≤ *x* ≤ 20,−10 ≤ *y* ≤ 10}, we choose

$$A_{F}=M_{F}\text{exp}\{-\frac{{\left(x+20\right)}^{2}}{10}-\frac{{\left(y-y_{C}-1\right)}^{2}}{10}\},$$ (14)

where *M*_*F*_ measures the magnitude of the exogenous concentration, $y_{C}$ is the leftmost position of the BM. The feedback factor *G* has an uptake term in the stroma. Denoting *u*_*G*,*s*_ as the uptake rate, we define

$$ExtSrc_{G}=-u_{G,S}[G]\chi_{S}$$ (15)

The definition of exogenous source of G is analogous. Suppose that the exogenous source for G is introduced from $T=T_{G_{1}}$ to $T=T_{G_{2}}$, we define

$$ExtSrc_{G}=-u_{G,S}[G]\chi_{S}+\alpha_{G}\left(A_{G}-[G]\right)\chi_{S}$$ (16)

for *T*_*G1*_≤*T*≤T_G2_. Similar to *F*,*G* is produced in the stroma at a rate proportional to its difference from a target concentration

$$A_{G}=M_{G}\text{exp}\{-\frac{{\left(x+20\right)}^{2}}{50}-\frac{{\left(y-y_{C}-1\right)}^{2}}{50}\}$$ (17)

with proportionality constant *α*_*G*_ Here *M*_*G*_ measures the magnitude of the exogenous concentration of *G*, *y*_*c*_ is the leftmost position of the BM. The values for these dimensionless parameters are listed in S1 Table.

## Supporting Information

S1 Table List of dimensionless parameters for spatial simulations in Figs 3–8. (DOCX)

S1 Text Supporting information. Derivations of analytical results, and description of spatial modeling and analysis.
(DOCX)

S1 Fig The critical feedback ratio is a constant. Within each of the bistable states, parameter choices for *ϕ* and *γ* can control the steady state **(A)** or the dynamics to the steady state **(A, B)**, but the critical ratio between the two states remains constant as indicated by the linear threshold in **(C)**. Parameters and initial conditions were set to *p* = 1, *d* = 1, *χ*_0_(0) = 1, and *χ*_1_(0) = 0.
(PDF)

S2 Fig The basis for initial condition dependence. If enough *χ*_0_(0) differentiates into *χ*_1_, *χ*_1_ can undergo a growth leap even if *χ*_1_(0) was initially insufficient. This is illustrated in **(A)** where a growth leap occurs even though *χ*_1_(0) = 0. Initial *χ*_0_(0) is large enough to carry *χ*_1_ to *χ*_1_^*critlow*^, and consequently *χ*_1_ then leaps to *χ*_1_^*crithigh*^. The derivative of the stem cell population plotted in **(B)** switches signs, as seen in **(C)**, as *χ*_1_’s curve reaches its inflection points. Here, its second derivative switches signs **(E)**. Lastly, we can confirm that *χ*_1_ is integrating *χ*_0_’s growth because its derivative **(D)** matches *χ*_0_’s trajectory in (B). Parameters values in (A-E) were set to *p* = 0.8, *ϕ* = 0.05, and *γ* = 0.002.
(PDF)

S3 Fig Final state ultrasensitivity with respect to feedback. **(A, B)** Positive and negative feedback strengths on self-renewal can be adjusted to toggle a switch in the final state. The solid black line indicates a stable final state solution while the dashed red line indicates an unstable final state solution. Parameter values are *p* = 0.8, *ϕ* = 0.05, and *γ* = 0.002.
(PDF)

S4 Fig Bistability and bimodality of a two-stage lineage with positive feedback originating from stem cells. **(A-D)** When the death rate is non-zero, the system is bistable. Parameter values in panels (A-D) are *δ* = 0.01, *p* = 0.78, *ϕ* = 2, and *γ* = 0.0000562. **(E, F)** When the death rate is zero, the system is bimodal. Parameter values in panels (E, F) are *δ* = 0, *p* = 0.8, *ϕ* = 1.75, and *γ* = 0.005. The initial conditions in panels (E, F) are *χ*_0_(0) = 1 and *χ*_1_(0) = 0.
(PDF)

S5 Fig Bistability and bimodality in three stage lineages with positive and negative feedback. The CP and TD stages exhibit bistability with respect to positive feedback gain *ϕ* (**A**-**B**), negative feedback gain *γ* (**C**-**D**), and the stem cell population initial condition (**E**-**F**). Parameters are *ζ* = 0.09, *δ* = 0.2, *p*_*1*_ = 0.75, and *ϕ* = 2.5 or *γ* = 0.02, when not fixed. Growth bimodality is also observed if stem cell turnover is set to zero (*p*_*0*_ = 0) or made very slow. Panels **(G)** and **(H)** plot final state systems in which the stem cell mitosis rate and TD death are zero and *p*_*0*_ = 0, *p*_*1*_ = 0.8, and *ϕ* = 0.45 or *γ* = 0.0012, when not varied. Initial conditions are *χ*_0_(0) = 1, *χ*_1_(0) = 4, and *χ*_2_(0) = 8.
(PDF)

S6 Fig Evolution of an epithelium with a positive feedback gain of *ϕ* = 3.0. Evolution in time is shown for positive and negative feedback factors, the self-renewal fraction, and the spatial distribution of SCs, CPs and TDs. Panel **(A)** shows the distribution of positive feedback factors. Panel **(B)** shows the distribution of negative feedback factors. Panel **(C)** shows the distribution of the self-renewal fraction of CPs. The distributions of SCs are shown in **(D)**, CPs are shown in **(E)** and TDs cells are shown in **(F)**. λ is the diffusional length of feedback factor G. Although at early times the epithelium grows and cells stratify spatially within the epithelium, growth is not sustained, however, due the effects of negative feedback in the system. Endogenous sources of positive feedback factors are insufficient to sustain the self-renewal of CPs. CPs consequently differentiate into TDs and growth does not self-sustain itself.
(PDF)

S7 Fig Evolution of an epithelium with a positive feedback gain of *ϕ* = 4.0. Evolution in time is shown for positive and negative feedback factors, the self-renewal fraction, and the spatial distribution of SCs, CPs and TDs. Panel **(A)** shows the distribution of positive feedback factors. Panel **(B)** shows the distribution of negative feedback factors. Panel **(C)** shows the distribution of the self-renewal fraction of CPs. The distributions of SCs are shown in **(D)**, CPs are shown in **(E)** and TDs cells are shown in **(F)**. λ is the diffusional length of feedback factor G. With this larger positive feedback gain of *ϕ* = 4.0, epithelial growth is self-sustained as CPs and TDs are distributed uniformly throughout the tissue.
(PDF)

S8 Fig Evolution of an epithelium with local and transient application of an exogenous positive regulator up to T = 12, where in addition to TD cells, CPs also produce endogenous negative feedback factors. CPs produce negative feedback factors at a rate of 0.3, while the production rate of the negative feedback factors by TD cells is decreased from 0.5 (when TD cells were the only source of a negative regulator) to 0.35. TD cell production of negative feedback is set to a lower rate in order to keep the overall level of negative feedback at roughly the same level as before. The distributions of the positive and negative factors are shown in **(A)** and **(B)**, respectively. The self-renewal fraction of CPs is shown in **(C)**. In **(D—F)**, the distributions of CPs and TDs are shown. λ is the diffusional length of feedback factor G. CPs are less concentrated at the BM, because CPs produce negative feedback factors that promote differentiation. As a result, the epithelium is less stratified than the epithelium simulated in Fig 4 in the main text.
(PDF)

S9 Fig Evolution of an epithelium with local and transient application of an exogenous positive regulator up to T = 12, where in addition to CPs, TD cells also produce endogenous positive feedback factors. TD cells produce positive feedback factors at a rate of 0.5, while the production rate of positive feedback factor by the CPs is decreased from 5.0 (when CPs were the only source of a positive feedback) to 4.0, in order to compensate for an excess of positive feedback. CPs production of negative feedback is set to a lower rate in order to keep the overall level of negative feedback at roughly the same level as before. The distributions of the positive and negative factors are shown in **(A)** and **(B)**, respectively. The self-renewal fraction of CPs is shown in **(C)**. In **(D—F)**, the distributions of CPs and TDs are shown. λ is the diffusional length of feedback factor G. CPs are concentrated at the BM but also appear near the AP because the positive feedback factors produced by TDs sustain high CP self-renewal. However, the amount of endogenous positive feedback is insufficient to elevate CP self-renewal above 0.5, so most CPs near the AP still differentiate into TDs. As a result, the epithelium remains spatially stratified.
(PDF)

S10 Fig Evolution of an epithelium with local and transient application of an exogenous positive regulator up to T = 12 in which positive feedback factors have a lower diffusivity (*D_F_* = 0.01). In the main text, all of the presented spatial simulations have diffusivities of positive feedback factors set as equal (*D_F_* = *D_G_* = 1.0). Here a similar epithelial simulation is achieved with *D_F_* = 0.01 and a slightly smaller production rate of the positive feedback factor *F*. A transient exogenous source of feedback factors is applied up to T = 12 as in Fig 5A in the main text. The distributions of the positive and negative factors are shown in **(A)** and **(B)**, respectively. The self-renewal fraction of CPs is shown in **(C)**. In **(D—E)**, the distributions of CPs and TDs are shown. λ is the diffusional length of feedback factor G. The application of positive regulator by an exogenous source is sufficient to ignite self-sustained, spatially stratified growth, although positive feedback factor F is more sharply stratified across the epithelium.
(PDF)

S11 Fig Evolution of an epithelium in which the stroma no longer behaves as a negative regulator sink with local and transient application of an exogenous positive regulator up to T = 12. The area of total epithelium is plotted as a function of time. In the main text, negative feedback factors are assumed to be bound to the stroma by Follistatin (FST); this binding is modeled by a sink term in the equations. Here we show that similar results can be obtained if this sink is turned off. Specifically, if this sink is simply turned off, the growth of the epithelium is no longer sustained (as indicated by the black curve), but if the production rate of negative feedback factor from TDs is decreased from 0.5 to 0.45, growth does self-sustain (as indicated by the green curve).
(PDF)

S12 Fig Estimation of the critical feedback ratio. A comparison of the feedback ratios using the diffusing positive and negative feedback signals. The feedback ratios are plotted as functions of time (measured in CP cell cycles), as they develop spatially for different positive feedback strengths, *ϕ/γ*. The ratio is calculated as max *ϕ* [*F*] / (*γ* [*G*] + ε), where ε = 10^−3^ is a small number, [*F*] and [*G*] are the concentrations of the positive and negative feedback regulators, and the maximum is taken within the epithelium. **(A)** Deterministic growth with positive feedback gains of *ϕ* = 3.0 and *ϕ* = 4.0. The feedback ratio is shown as functions of time and the distributions of CP self-renewal at the 0^th^, 12^th^ and 24^th^ CP cell cycles are shown as insets. Growth is not self-sustaining when *ϕ* = 3.0 because the positive feedback produced by the CPs is insufficient to sustain CP self-renewal (green curve). The epithelium with *ϕ* = 4.0 grows similarly at early times and continues to grow exponentially at later times driven by positive feedback on CP self-renewal. CPs produce enough positive feedback factor to drive the ratio of positive-to-negative feedback over a critical threshold for self-sustaining growth (blue curve). **(B)** Growth with stochastic *p*, as in Fig 3 of the main text. The positive feedback gain is *ϕ* = 3.0. With small variance (0.1), growth is not self-sustaining as in panel A. With larger variance, the growth is self-sustaining as the randomness increases the feedback ratio above the critical threshold. Larger variance results in faster growth.
(PDF)

S13 Fig Evolution of the cell distributions in an epithelium with local and transient application of an exogenous positive regulator. λ is the diffusional length of feedback factor G. The positive regulator is produced exogenously from a localized source until time T = 12. Growth self-sustains within a region of the epithelium near the positive regulator’s source, and cell distributions spatially stratify such that more CPs are located in the growing region while more TDs are located in the non-growing region. Both self-sustained growth and stratification occur both during application of the exogenous signal and after removal of the exogenous source.
(PDF)

S14 Fig Evolution of the CP self-renewal fraction in an epithelium with local and transient application of an exogenous positive regulator. λ is the diffusional length of feedback factor G. The positive regulator is produced exogenously from a localized source until time T = 12. A green contour indicates the epithelial interface, and a cyan contour represents where P = 0.5. An exogenous regulator causes an area near the regulator’s source to take on high P values. The CP population therefore is primarily undergoing self-renewal in the region of self-sustaining growth.
(PDF)

S15 Fig Evolution of an epithelium with local and transient application of an exogenous positive regulator for durations of 8, 10, and 12 CP cell cycles. Total areas of the epithelium are shown as functions of time. λ is the diffusional length of feedback factor G. The exogenous source of positive regulator is removed at TF = 8, 10 and 12, as labeled. Epithelial growth does not self-sustain if the exogenous source of positive regulator is removed at T = 8 or T = 10. However, when the source is removed at T = 12, growth self-sustains and the area of the total epithelium continues to increase. Insets show the morphologies of the epithelia and the spatial distribution of positive feedback factors at T = 30.
(PDF)

S16 Fig A comparison of the positive-to-negative feedback ratios in evolving epithelia in which local and transient applications of an exogenous positive regulator occurred for durations of 8, 10, and 12 CP cell cycles. The ratio is calculated as max_BM_*ϕ*[*F*]/(*γ*[*G*]+*ε*), where *ε* = 10^−3^ and the maximum is taken along the BM. The critical threshold estimated in S12 Fig is marked by the dashed line. The exogenous source of positive feedback is removed at TF = 8, 10 and 12 respectively. When the exogenous source is removed, the ratio drops due to the removal of positive feedback. If the source is removed at TF = 8 (black curve) or TF = 10 (red curve), the ratio steadily decreases below the critical threshold, and growth is not sustained. However, if the source is removed at TF = 12 (green) or is applied continuously (magenta), the ratio stays above the critical threshold for the duration of the simulation. The epithelium corresponding to the green curve grows out of the computational domain before T = 30, and therefore its presented trajectory in the figure is not as long as the others.
(PDF)

S17 Fig Evolution of the cell distributions in an epithelium with transient application of an exogenous negative regulator. The development in time of the spatial distributions of SCs, CPs and TDs are presented here. λ is the diffusional length of feedback factor G. Until time T = 15, the simulation is the same as in S13 Fig, but from time T = 15 to T = 18, a negative regulator is applied exogenously. Growth is subsequently terminated even after the negative regulator is removed, and the epithelium is comprised primarily of TDs at late times. The evolution of feedback factors and the self-renewal fraction of CPs maybe found in Fig 5B in the main text. The spatial stratification seen up until T = 15 is lost afterwards as CPs differentiate into TDs.
(PDF)

S18 Fig Evolution of the CP self-renewal fraction in an epithelium with transient application of an exogenous negative regulator. λ is the diffusional length of feedback factor G. Until time T = 15, the simulation is the same as in S13 Fig, but from time T = 15 to T = 18, a negative regulator is applied exogenously. A cyan contour represents where P = 0.5. The exogenous application of negative regulator drives P below 0.5, which causes CPs to differentiate. A reduction in CPs reduces endogenous positive feedback, and growth ceases after self-renewal in CPs sufficiently declines.
(PDF)

S19 Fig Evolution of an epithelium with transient application of an exogenous negative regulator with different signal strengths. The signal strengths are indicated by peak concentrations of the negative regulator, which are M_G_ = 0.5, 6, and 16. The area of the total epithelium is plotted as a function of time. λ is the diffusional length of the feedback factor G. From T = 15 to T = 18, a exogenous negative regulator is applied. When the peak concentration of the exogenous negative feedback factors is 16.0 (black) or 6.0 (blue), the area of the total epithelium increases transiently and then steadily drops, indicating that the growth is not self-sustaining. When the peak is 0.5 (magenta), the area steadily increases, indicating that growth is still self-sustained. Insets show the spatial distribution of positive feedback factors at T = 24.
(PDF)

S20 Fig A comparison of the positive-to-negative feedback ratios in evolving epithelia in which transient applications of an exogenous negative regulator occurred for different durations and signal strengths. The ratio is calculated as max_BM_*ϕ*[*F*]/(*γ*[*G*]+*ε*), where *ε* = 10^−3^ and the maximum is taken along the BM. The critical threshold predicted by S12 Fig is marked by the dashed line. When the exogenous source of positive feedback factors is removed at T = 12, the ratio immediately decreases. At T = 15, the ratio drops again because exogenous negative feedback is applied. When the peak concentration of the exogenous negative feedback factors is 16.0 (black) or 6.0 (blue), the ratio drops because most CPs differentiate, and growth is subsequently extinguished. When the peak concentration of the exogenous negative feedback factors is 0.5 (magenta), the ratio stays above the critical threshold after the source is removed, indicating that the growth is sustained (see also S19 Fig).
(PDF)

S21 Fig The area of the epithelium and its cellular components are shown as a function of time (in CP cell cycles) for the simulations shown in Figs 3–5 with positive feedback gain *ϕ* = 3.0. In **(A)**, the total areas of the epithelium from Fig 3E (black; no exogenous signaling factors), from Fig 4 (blue; exogenous source of positive feedback factors), from Fig 5A (green; transient exogenous source of positive feedback factors), and Fig 5B (red; transient exogenous source of negative feedback factors) are shown. In **(B)** and **(C)** the corresponding areas of the CPs and TDs, respectively, are shown. Note that the area of stem cells is conserved in all cases.
(PDF)

S22 Fig A finger of constant width elongates with a continuous and local application of an exogenous source of positive regulator, see Fig 7A–7C in the main text. λ is the diffusional length of negative feedback factor *G*. The black contour indicates the epithelial interface. Panel **(A)** shows the time evolution of the CP self-renewal ratio, P, (indicated by the gray scale gradient) upon application of an exogenous positive regulator (indicated by a blue-purple gradient). Panel **(B)** shows the spatial distributions of cell components, positive regulation factors (where the green-cyan gradient indicates the concentration of endogenous feedback and the blue-purple gradient indicates the exogenous regulator’s concentration) and negative regulators (indicated by the red-yellow gradient) at T = 75.
(PDF)

S23 Fig A finger of constant width elongates with local and transient application of an exogenous positive regulator that ceases at T = 15, see Fig 7E–7G in the main text. λ is the diffusional length of negative feedback factor *G*. The black contour indicates the epithelial interface. Panel **(A)** shows the time evolution of the CP self-renewal ratio, P, (indicated by the gray scale gradient) upon application of an exogenous positive regulator (indicated by a blue-purple gradient). Panel **(B)** shows the spatial distributions of cell components, positive regulation factors (where the green-cyan gradient indicates the concentration of endogenous feedback and the blue-purple gradient indicates the exogenous regulator’s concentration) and negative regulators (indicated by the red-yellow gradient) at T = 59.
(PDF)

S24 Fig Self-organizing pattern formation can be triggered by initial geometric noise. The evolution of epithelia and the distributions of endogenous positive feedback factors *G* (green). The black contour indicates the epithelial interface. The scale bar is proportional to the diffusion length (λ) of *G*, as labeled. In (A)-(B) panels, the equation defining the initial location of the BM membrane is given by 2.0 + 0.1[*Sin*^2^(3*ξ*) + *Cos*^2^(5 *ξ*) + *Sin*(13 *ξ*) + *Cos*(19 *ξ*) + *Sin*(23 *ξ*)] (see section 7 of S1 Text). In **(A)**, the feedback gains are *ϕ* = 3.0 and *γ* = 5.0, i.e. *ϕ*/*γ* = 0.6. Snapshots of the simulation are shown that provide a more detailed view of the evolution in time for the simulation presented in the main text in (Fig 8H–8N). In **(B)** the feedback ratio is increased using *ϕ* = 3.5 and *γ* = 5.0, i.e. *ϕ*/*γ* = 0.7. This panel also shows a more detailed view of the evolution in time for the simulation presented in the main text in (Fig 8O–8U). In **(C),** the diffusivities of *F* and *G* are varied as labeled. Decreasing *D*_*F*_ or increasing *D*_*G*_ results in thinner fingers. Increasing *D*_*F*_ makes *F* more diffusive, and the epithelium does not grow because the magnitude of *F* is too low to sustain growth. **(D)** The total areas of a sinusoidally perturbed epithelium as a function of time with different diffusivities of positive feedback factors, *D*_F_, as labeled. The perturbation amplitude is 1.5. When *D*_F_ = 0.01 or 0.02, the area steadily increases and growth is sustained. In contrast, when *D*_F_ = 0.1 or 1.0, the epithelium grows transiently, but growth is not sustained. Insets show the spatial distribution of positive feedback factors at T = 0, 20 and 30. When *D*_F_ = 0.01 or 0.02, the epithelium forms a bud where positive feedback factors are concentrated
(PDF)

S25 Fig A plot of both the exogenous and endogenous *F*, together with the adaptive mesh. Exogenous positive regulator is shown with a blue-purple gradient, and endogenous positive regulator is shown with a green-cyan gradient. The black line indicates the *χ*_*T*_ = 0.5 contour. The coarsest level has 32×16 grids. There are three levels of refinement around the *χ*_*T*_ = 0.5 contour; each level has twice as many grid points as the parent level.
(PDF)
