# Supplementary material for: Feedback, Lineages and Self-Organizing Morphogenesis
Source: PLoS Comput Biol. 2016 Mar 18;12(3):e1004814. doi: 10.1371/journal.pcbi.1004814 (PMC4798729; doi:10.1371/journal.pcbi.1004814)
Supplement: S1 Table — (DOCX) [file pcbi.1004814.s001.docx]

**S1 Table. List of dimensionless parameters.**

| **Mechanical properties** | |
| --- | --- |
| Cell Mobility | $M=5.0$ |
| Adhesion force at BM | $\lambda_{1}=-0.03$ |
| Adhesion force at AP | $\lambda_{2}=0.2$ |
| Diffuse interface thickness | $\varepsilon=0.05$ |

| **Source terms** | |
| --- | --- |
| Stem cell division rate | $v_{0}=0.01$ |
| TDs death rate | $d=0.02$ |

**For Figures 3, 4, and 5**

| **Feedback control** | |
| --- | --- |
| Minimum CP self-renewal rate | $p_{1,\min}=0.02$ |
| Maximum CP self-renewal rate | $p_{1,\max}=1.0$ |
| Positive feedback gain | $\varphi=3.0$ |
| Negative feedback gain | $\gamma=1.0$ |

| **Feedback factors** | |
| --- | --- |
| Production rate of F by CPs | $s_{F}^{1}=5.25$ |
| Production rate of F by TDs | $s_{F}^{2}=0$ |
| Natural decay rate of F | $d_{F}=1.0$ |
| Diffusivity of F | $D_{F}=1.0$ |
| Production rate of G by CPs | $s_{G}^{1}=0.0$ |
| Production rate of G by TDs | $s_{G}^{2}=0.5$ |
| Natural decay rate of G | $d_{G}=1.0$ |
| Uptake rate of G by the stroma | $u_{G,S}=1.0$ |
| Diffusivity of G | $D_{G}=1.0$ |

| **Exogenous sources** | |
| --- | --- |
| Exogenous F source starts from | $T_{F1}=0$ |
| Exogenous F source ends at | $T_{F2}=12$ |
| Exogenous F production rate | $\alpha_{F}=5.0$ |
| Magnitude of exogenous F | $M_{F}=60.0$ |
| Exogenous G source starts from | $T_{G_{1}}=15$ |
| Exogenous G source ends at | $T_{G_{2}}=18$ |
| Exogenous G production rate | $\alpha_{G}=5.0$ |
| Magnitude of exogenous G | $M_{G}=16.0$ |

**For Figure 7 & 8**

| **Source terms** | |
| --- | --- |
| Stem cell division rate | $v_{0}=0.1$ |
| TDs death rate | $d=0.02$ |

| **Feedback control** |
| --- |

|  |  |
| --- | --- |
| Maximum CP self-renewal rate | $p_{1,\max}=1.0$ |
| Positive feedback gain  (Figure 7A-D) |  |
| Negative feedback gain  (Figure 7A-D) | $\gamma=3.0$ |
| Positive feedback gain  (Figure 7E-H) | $\gamma=2.5$ |
| Negative feedback gain  (Figure 7E-H) | $\gamma=3.0$ |
| Positive feedback gain  (Figure 8A – N, 8V, 8W) |  |
| Negative feedback gain  ( Figure 8A – N, 8V, 8W ) | $\gamma=5.0$ |
| Positive feedback gain  (Figure 8O – U, 8V, 8W ) |  |
| Negative feedback gain  (Figure 8O – U, 8V, 8W ) | $\gamma=5.0$ |

| **Feedback factors** | |
| --- | --- |
| Production rate of F by CPs | $s_{F}^{1}=5.25$ |
| Production rate of F by TDs | $s_{F}^{2}=0$ |
| Natural decay rate of F | $d_{F}=1.0$ |
| Diffusivity of F | $D_{F}=0.1$ |
| Production rate of G by CPs | $s_{G}^{1}=0.0$ |
| Production rate of G by TDs | $s_{G}^{2}=0.5$ |
| Natural decay rate of G | $d_{G}=1.0$ |
| Uptake rate of G by the stroma | $u_{G,S}=600.0$ |
| Diffusivity of G | $D_{G}=0.1$ |

| **Exogenous sources** | |
| --- | --- |
| Exogenous F source starts from | $T_{F1}=0$ |
| Exogenous F source ends at  (Figure 7F) | $T_{F2}=15$ |
| Exogenous F production rate | $\alpha_{F}=5.0$ |
| Magnitude of exogenous F | $M_{F}=160.0$ |
