# Supplementary material for: Feedback, Lineages and Self-Organizing Morphogenesis
Source: PLoS Comput Biol. 2016 Mar 18;12(3):e1004814. doi: 10.1371/journal.pcbi.1004814 (PMC4798729; doi:10.1371/journal.pcbi.1004814)
Supplement: S1 Text — Derivations of analytical results, and description of spatial modeling and analysis. (DOCX) [file pcbi.1004814.s002.docx]

**Supplementary Materials**

**Table of Contents**

**§1 An *n*-stage lineage.** ............................................................................................... 2

**§2 Negative feedback alone does not result in bimodal growth.** ........................... 3

**§3 Analytical solutions for the final state.** ................................................................ 4

**§4 Critical values delineate distinct regions of behavior.** ....................................... 5

**§5 Alternative ways of combining positive and negative feedback.** ...................... 6

**§6 Description of the spatial model.** ……………........................................................ 11

**§7 Description of the BM initial contours in Figure 8.** ............................................. 14

**§8 Description of the Fourier transform and the spectral moment.** ....................... 15

**References** ................................................................................................................... 16

**§1 An *n*-stage lineage.**

The dynamics of an arbitrary stage in an stage lineage can be described generically as a first-order differential equation where is the proliferation rate, and is the rate at which cells leave via differentiation, and is the rate at which differentiating progenitor cells enter a stage where . The lineage starts with , a stem cell (SC) stage, and ends with , a terminally differentiated (TD) stage.

(Eq. S1)

Although lineages can theoretically contain any number of stages, a lineage can fragment due to various barriers introduced through the course of development. Selective barriers such as growth inhibition and a requirement for blood supply are mentioned in [[1](#_ENREF_1)] are impediments to clonal expansion where lineages may reach a dead end. Thus, it is not only more amenable for analysis to use a simple two-stage lineage for Eq. 1, but shorter lineages are likely more common than longer ones.

**§2 Negative feedback alone does not result in bimodal growth.**

Feedback regulation of self-renewal is modeled using Hill kinetics such that progenitor self-renewal is a function of the TD population size, in lieu of diffusible factors which nonspatial ODEs cannot simulate. In the case of negative feedback, replaces P in Eq. 1 and sets the feedback gain from . Solving for the equilibria yields a trivial zero-growth solution and a non-zero solution.

(Eq. S2)

This system is monostable because only one non-zero stable solution exists. There is no hope for growth ultrasensitivity (and hence bimodality) if a system’s steady state is monostable and responds hyperbolically to the gain of a growth factor, which is precisely the case for the nonzero equilibria (we indicate equilibria with an asterisk). Furthermore, an ultrasensitivity cannot occur at , which delineates the first trivial steady state solution when from the second nonzero steady state solution when , because their limits are equal.

(Eq. S3)

Since both equilibriums and their transition point precludes ultrasensitive or discontinuous behavior, negative feedback by itself on self-renewal is a poor strategy for achieving differential growth through the lineage.

**§3 Analytical solutions for the final state.**

The final state solution for the two-stage lineage model in which self-renewal is only regulated by negative feedback (where replaces P in Eq. 1 and sets the feedback gain from ) agrees with the steady state; they are both continuously differentiable. It solves to

(Eq. S4)

where *W* is the product log function.

The final state for the model illustrated in Figure 2A, on the other hand, cannot be solved explicitly. The implicit equation that describes the final state, however, reveals that it is undefined for certain parameter values, and numerical simulations show that this is where discontinuous behavior arises (See Figure 2).

(Eq. S5)

**§4 Critical values delineate distinct regions of behavior.**

The critical values, and from Eq. 8 in the final state analysis, define the direction of ’s trajectory and set ’s inflection points wherever switches signs.

Inspection of these derivatives reveals a discrete separation of growth states. While is below or above , will decelerate from the onset and as shrinks (Figure 2C). When reaches zero, plateaus to its final state. The region between these critical values however is where growth leaps occur. Whenever enters the growth leap region in between, it is pushed upwards and out. Here, grows and populates as it differentiates until eventually breaches , at which point ’s derivative switches signs and shrinks to zero (Figure 2C). Upon exiting through , inflects from concave up to down and is permitted to decelerate to a plateau again.

**§5 Alternative ways of combining positive and negative feedback.**

Only one specific way of combining negative and positive feedback in the two stage lineage was presented in the main text. Bimodality and bistability, however, are not unique to this form. Tables S1 and S2 below summarize the outcomes (in terms of bistability and bimodality) of combining the opposing feedback loops in various ways. One can see that the results of the model presented in the paper are general for all feedback configurations. Following these tables, two prototypical cases are shown as examples:

(*Example 1*) a case that is also bistable and bimodal and

(*Example 2*) a case that is also bimodal, even though the larger population mode does not occur at a stable equilibrium.

For cases in which the presence or lack of bistability or bimodality could not be determined analytically, this was determined by iteratively checking random parameter values.

**Table A. Bistability or bimodality is an outcome for all cases in which positive and negative feedback originate from the TD cell (*χ_1_*).** The last two rows, in which positive feedback inhibits negative feedback, results in either a stable non-zero state or, when *φ*/*γ* is high, a destabilization that causes cell populations to grow without bound.

**Table B. Bistability or bimodality is an outcome for all cases in which positive feedback originates from the progenitor cell (*χ_0_*) and negative feedback originates from the TD cell (*χ_1_*).** The last two rows, in which positive feedback inhibits negative feedback, results in either a non-zero final state or, when *φ*/*γ* is high, a destabilization that causes cell populations to grow without bound.

*Example 1*:

When χ_0_ is the source of positive feedback, which combines with negative feedback in the same way as the model in the main text, i.e. , the system is also bistable and bimodal, as seen in Fig. S4.

*Example 2*:

The analysis that follows explores the feedback form given by .

A two-stage lineage regulated by this feedback only has two equilibrium states.

(1)

(2)

A system with only one non-zero equilibrium cannot be bistable. However, eigenvalues of the ODE’s Jacobian linearized about the non-zero equilibrium reveal that it is always unstable when its value is positive. These eigenvalues are given by

.

It can be seen that the real parts of both of these eigenvalues cannot be negative (a requirement for stability) while , which is the requirement for positive equilibrium values. In other words, the non-zero equilibrium is unstable and the system therefore grows without bound. Although such a system is not bistable, it can be considered bi-modal since there is a clear distinction between a high and low cell population size in the output.

Similarly, the final state solution when *δ* is set to zero can be shown to be bimodal using the same definition (i.e. distinctly high and low cell populations in the output). Here, the final state (*χ*_1_(∞)) is given implicitly for *χ*_1_(0) by

.

Solving gives us only one stationary point, which is .

This stationary point determines the existence of the final state. When the expression within the log is negative (), the stationary point becomes imaginary and the final state exists for all starting stem cell numbers. However when the expression within the log is positive (), then the final state vanishes when .

When the final state vanishes, both cell populations undergo unbounded growth. Consequently, there are two distinct types of populations: one is a finite final state (i.e. small) and the other is large without limit (as a result of unbounded growth).

**§6 Description of the spatial model.**

**Numerical methods.** The time discretization is implemented by an implicit Crank-Nicholson scheme with second-order accuracy. This scheme removes the high-order time step constraint, and is found to be stable with in our context. Spatial derivatives are discretized using central difference approximations. The advection terms are treated using an upwind weighed ENO scheme [[2](#_ENREF_2)]. Block structured Cartesian refinement is used to efficiently resolve the multiple spatial scales, especially in regions with large gradients. The equations at implicit time level are solved by the nonlinear multigrid method developed in [[3](#_ENREF_3)].

The 2D spatial system is solved in a rectangular computational domain. The coarsest mesh has grid points, which yields a square mesh. We use three levels of mesh refinement; each level uses twice as many grid points as parent level. The mesh is refined by the undivided gradient test. In particular, we refine the mesh by adding a child mesh to the parent, where has a steep gradient at the BM and AP. These three levels of mesh refinement can be visualized in S25 Fig. The time step is.

**Volume fractions of the solid domain () and the stroma ().** We assume that the BM lies on the left edge of epithelium, and the AP is on the right edge. Letdenote the largest such that. We define if ; otherwise, and

Thus is the volume fraction of the epithelium plus the stroma on the left. The volume fraction of stroma is thus

**Modeling SCs**. We model the SCs by, where is chosen to maintain a constant number of SCs, and is an approximation of the surface characteristic function of the BM, . We first define the characteristic function of the region, i.e. for and otherwise. Assuming that the BM lies on the left edge of epithelium, and the BM is characterized by, we choose

Let where is the initial number of SCs calculated by integrating over the whole computational domain, is the current number at time. We take as the SCs population. In this way, the integral is constant throughout the simulation.

**Diffuse domain approach for no-flux conditions.** Using the diffuse domain approach [[4](#_ENREF_4), [5](#_ENREF_5)], we reformulate the equations for and by extending them to the computational domain in a way that enables the no-flux boundary condition to be accurately modeled. The diffuse domain approximation for is

where . The approximation for is identical except for different coefficients and exogenous sources. Note that the concentrations inshould be taken as and . Following [[4](#_ENREF_4), [5](#_ENREF_5)] it can be shown that these equations model the no-flux boundary condition at the apical surface.

**Nondimensionalization and simulation parameters.** Let and be the length and time scale respectively. We denote as the dimensionless gradient and as the dimensionless time. Following [[3](#_ENREF_3)], we rewrite the equation for the dimensionless concentration :

Let the diffusion length. By choosing the length scale and defining dimensionless production rate and natural decay rate, the equation for can be rewritten as

. (Eq. S6)

Analogously, the equation for can be rewritten as

, (Eq. S7)

where the dimensionless diffusion coefficient is , the production rate is and the natural decay rate is .

Next, we nondimensionalize the equation forand. Let, and be the nondimensionalized cell velocity, mobility and chemical potential respectively. We rewrite the equation for as

Here we choose the time scale and. Denoting the nondimensionalized stem cell division rate, the equation of is rewritten as

. (Eq. S8)

Analogously, we define the nondimensionalized death rate of as, and rewrite the equation for:

. (Eq. S9)

The dimensionless velocity satisfies

where is the dimensionless pressure. We choose, then

, (Eq. S10)

. (Eq. S11)

By dropping the prime notation in Eq. S6-S11, we obtain dimensionless equations of the main text.

The dimensionless parameters are listed below for the simulations in the main text. See Figures S8-11 in the supporting information for parameter variations.

**§7 Description of the BM initial contours in Figure 8.**

In Figure 8A, the contour of an epithelium is made irregular at the basement membrane by superimposing sine and cosine waves. The exact equation defining this contour is given by 2.0 + 0.1[*Sin*(3*ξ*) + *Cos*(5*ξ*) + *Sin*(7*ξ*) + *Cos*(11*ξ*)] where *ξ* = 2*π* *x*/40. The overall thickness of the initial geometry in (A) is greater than that in Figures 7A and 7E, and growth in the simulations in Figure 8 is consequently self-sustaining even if the endogenous feedback ratio is lower (*φ*/*γ* = 0.6) than the ratios in Figure 7.

In Figure 8H, the exact equation defining the BM contour is given by 2.0 + 0.1[*Sin*^2^(3*ξ*) + *Cos* ^2^(5*ξ*) + *Sin*(13*ξ*) + *Cos*(19*ξ*) + *Sin*(23*ξ*)] where *ξ* = 2*π* *x*/40. The endogenous feedback ratio (*φ*/*γ* = 0.6) however is identical to that of the simulation presented in Figure 8 panels (A - G).

In Figure 8O, the BM contour is initially perturbed by the same combination of frequencies as in (H), however, the endogenous feedback ratio is greater than the simulations previously presented in panels (A – N) due to an increase in the positive feedback gain (which makes *φ*/*γ* = 0.7).

Figures 8V and 8W include initial contours from Figures 8A, 8H, and 8O in addition to BM contours that are given by 2.0 + 0.1[*Sin*(A*ξ*) + *Cos*(B*ξ*) + *Sin*(C*ξ*) + *Cos*(D*ξ*) + *Sin*(E*ξ*)] where *ξ* = 2*π* *x*/40, and A,B,C,D, and E are arbitrarily chosen parameters. A total of twenty-one additional combinations of initial contour frequencies were simulated. Parameters A, B, C, D, and E are shown below for each curve that is plotted in Figures 8V, W.

[A, B, C, D, E] = [45.4826, 9.24909, 5.12617, 17.5532, 35.7058]

[A, B, C, D, E] = [10.1366, 36.1876, 11.0538, 37.4945, 44.1401]

[A, B, C, D, E] = [3.05171, 17.3083, 12.7299, 21.9140, 24.1992]

[A, B, C, D, E] = [36.1159, 4.05406, 37.0759, 7.26768, 46.7325]

[A, B, C, D, E] = [5.83339, 30.3245, 43.8062, 40.1290, 19.6916]

[A, B, C, D, E] = [5.40442, 31.4058, 26.3925, 20.9118, 27.9974]

[A, B, C, D, E] = [94.0283, 76.9192, 56.0816, 69.7754, 79.1801]

[A, B, C, D, E] = [99.2103, 95.8779, 86.5527, 69.0397, 44.2086]

[A, B, C, D, E] = [69.3841, 53.4180, 45.6607, 62.0921, 98.9013]

[A, B, C, D, E] = [84.9591, 90.6284, 64.0147, 43.9183, 50.4724]

[A, B, C, D, E] = [69.8421, 50.8449, 87.1151, 84.2931, 44.3655]

[A, B, C, D, E] = [27.5102, 25.3530, 30.4402, 23.1450, 30.2253]

[A, B, C, D, E] = [37.9784, 39.7585, 33.1082, 27.6206, 31.4260]

[A, B, C, D, E] = [35.3446, 23.0240, 27.7755, 37.2465, 32.0162]

[A, B, C, D, E] = [21.0016, 32.6084, 23.3621, 25.6377, 28.8066]

[A, B, C, D, E] = [28.7384, 36.4007, 27.6503, 29.1271, 38.7435]

[A, B, C, D, E] = [4.613, 2.43062, 0.575182, 9.74374, 1.77486]

[A, B, C, D, E] = [0.137613, 0.816484, 9.70372, 9.89738, 3.52797]

[A, B, C, D, E] = [0.690956, 2.20329, 2.76966, 3.25803, 7.94816]

[A, B, C, D, E] = [0.428266, 9.92092, 0.170912, 7.72766, 0.179455]

[A, B, C, D, E] = [1.48536, 1.40593, 9.72656, 1.91466, 3.91556]

**§8 Description of the Fourier transform and the spectral moment.**

The discrete Fourier transforms (DFT) for the power spectra in Figure 8 panels E - G, L - N, and S - U were performed using *N* = 50 frequency components for the BM arc length contours shown in Figure 8D, 8K, and 8R, respectively. The number of frequency components used by the transform was increased to *N* = 200 in Figure 8V and Figure 8W to accommodate a wider range of measurable frequencies. The DFT was performed using the Fourier function in Mathematica 10.

The spectral moment of the DFT is defined here as

where *k_i_* is the frequency and is the corresponding Fourier coefficient at the *i*th component in a total of *N/2* frequency components. N is the total number of equally spaced samples from a function of the BM contour’s height at relative positions along the BM contour’s arclength. The formula for the spectral moment is used to calculate where power is centered within the spectrum.

**References**

1. Wodarz, D. and N. Komarova, *Can loss of apoptosis protect against cancer?* Trends Genet, 2007. **23**(5): p. 232-7.

2. Jiang, G.-S., Shu, C.-W., *Efficient implementation of weighted ENO schemes.* J. Comput. Phys., 1996. **126**: p. 202-228.

3. Wise, S.M., J.S. Lowengrub, and V. Cristini, *An Adaptive Multigrid Algorithm for Simulating Solid Tumor Growth Using Mixture Models.* Math Comput Model, 2011. **53**(1-2): p. 1-20.

4. Li, X., et al., *Solving Pdes in Complex Geometries: A Diffuse Domain Approach.* Commun Math Sci, 2009. **7**(1): p. 81-107.

5. Teigen, K.E., et al., *A Diffuse-Interface Approach for Modeling Transport, Diffusion and Adsorption/Desorption of Material Quantities on a Deformable Interface.* Commun Math Sci, 2009. **4**(7): p. 1009-1037.
