# Supplementary figures and images for: Feedback, Lineages and Self-Organizing Morphogenesis
Source: PLoS Comput Biol. 2016 Mar 18;12(3):e1004814. doi: 10.1371/journal.pcbi.1004814 (PMC4798729; doi:10.1371/journal.pcbi.1004814)

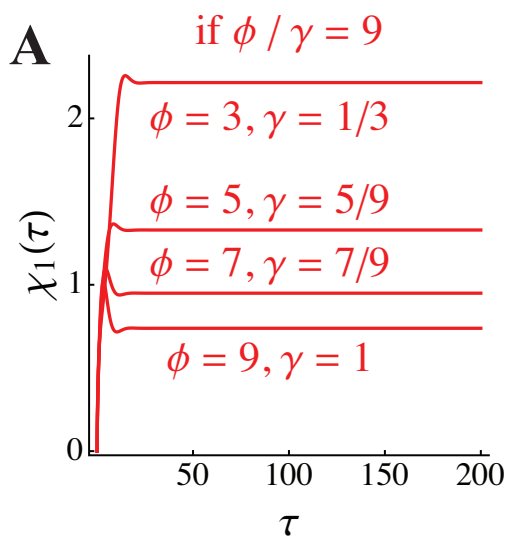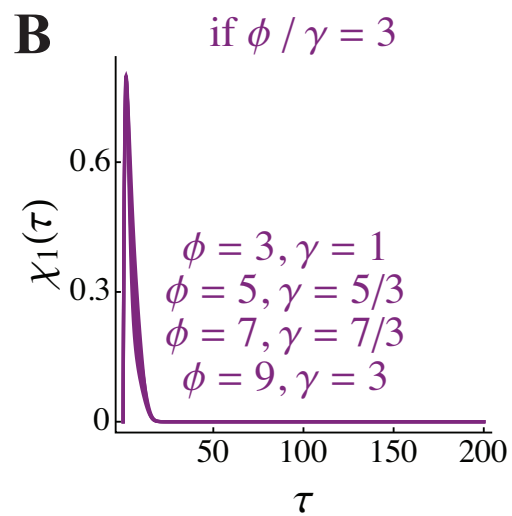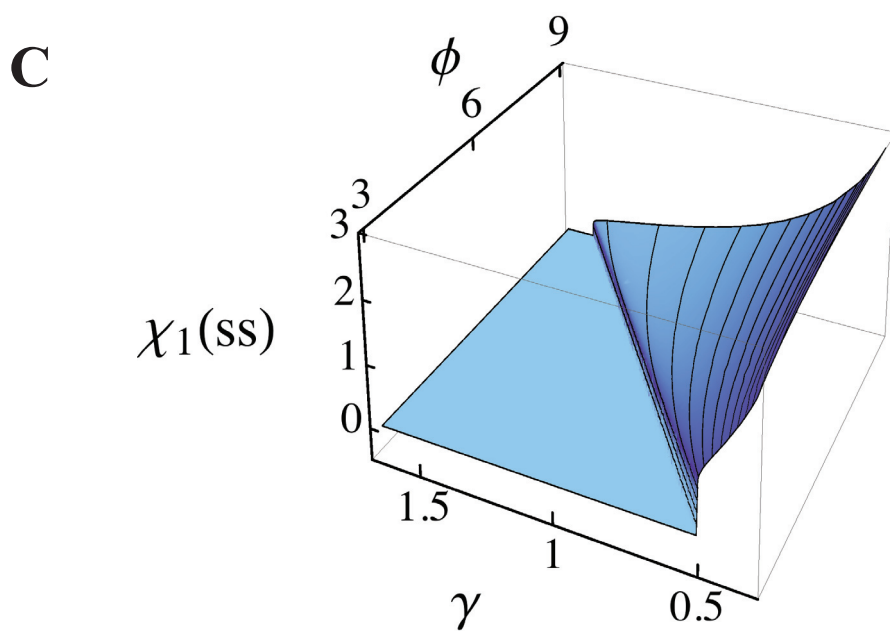

Supplement: S1 Fig — Within each of the bistable states, parameter choices for ϕ and γ can control the steady state (A) or the dynamics to the steady state (A, B), but the critical ratio between the two states remains constant as indicated by the linear threshold in (C). Parameters and initial conditions were set to p = 1, d = 1, χ0(0) = 1, and χ1(0) = 0. (PDF) [file pcbi.1004814.s003.pdf]

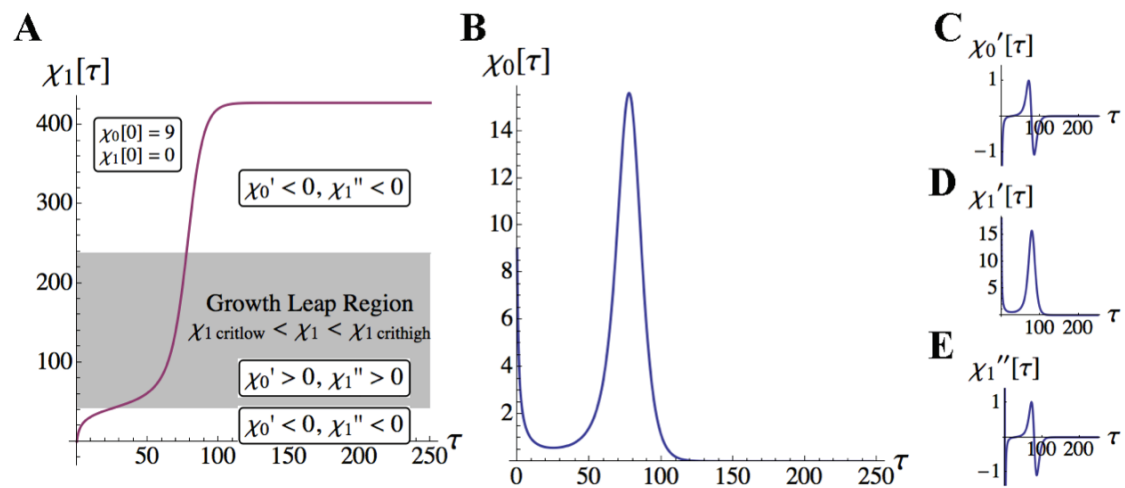

Supplement: S2 Fig — If enough χ0(0) differentiates into χ1, χ1 can undergo a growth leap even if χ1(0) was initially insufficient. This is illustrated in (A) where a growth leap occurs even though χ1(0) = 0. Initial χ0(0) is large enough to carry χ1 to χ1critlow, and consequently χ1 then leaps to χ1crithigh. The derivative of the stem cell population plotted in (B) switches signs, as seen in (C), as χ1’s curve reaches its inflection points. Here, its second derivative switches signs (E). Lastly, we can confirm that χ1 is integrating χ0’s growth because its derivative (D) matches χ0’s trajectory in (B). Parameters values in (A-E) were set to p = 0.8, ϕ = 0.05, and γ = 0.002. (PDF) [file pcbi.1004814.s004.pdf]

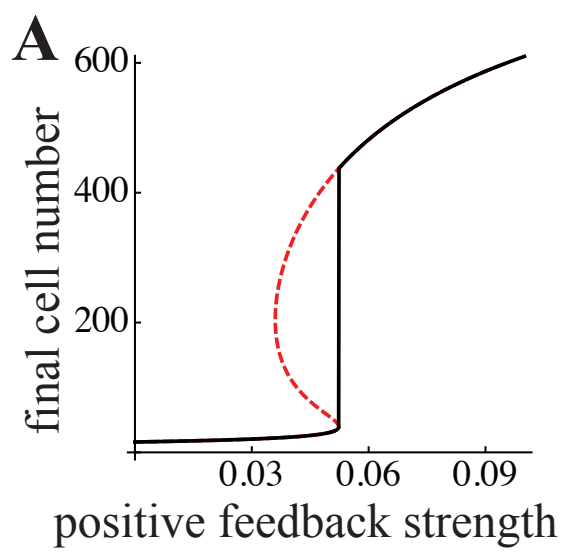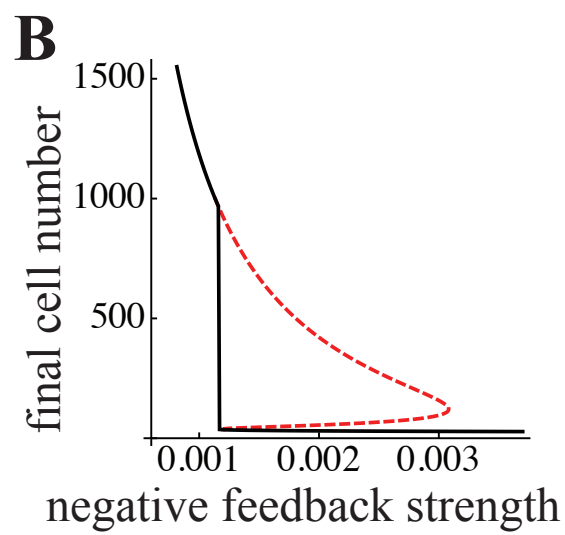

Supplement: S3 Fig — (A, B) Positive and negative feedback strengths on self-renewal can be adjusted to toggle a switch in the final state. The solid black line indicates a stable final state solution while the dashed red line indicates an unstable final state solution. Parameter values are p = 0.8, ϕ = 0.05, and γ = 0.002. (PDF) [file pcbi.1004814.s005.pdf]

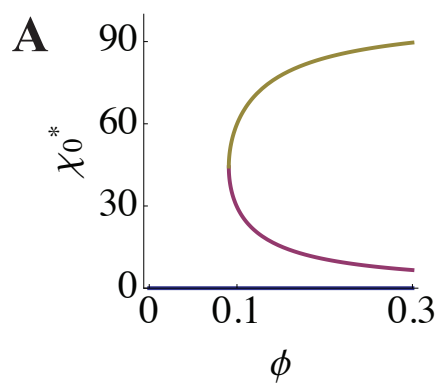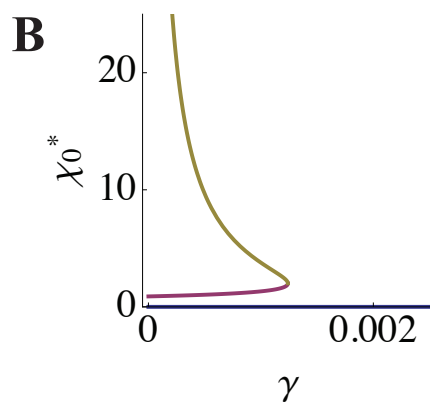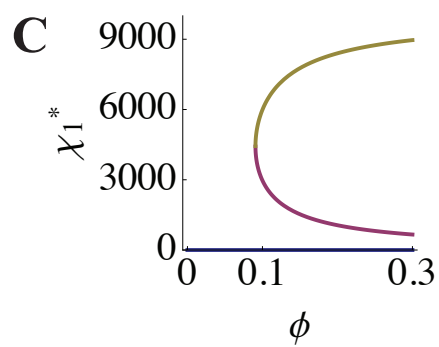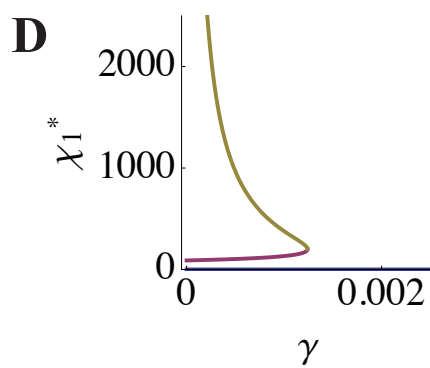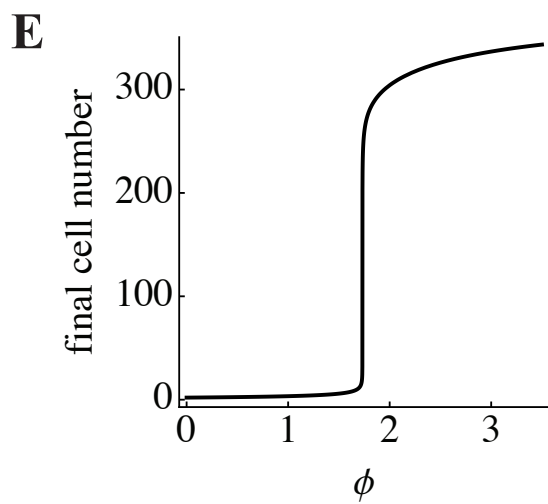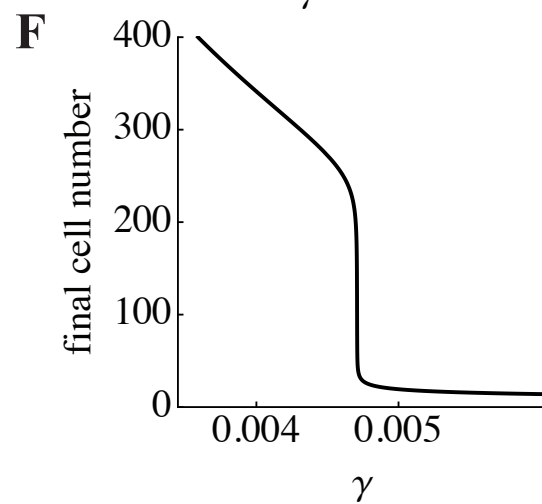

Supplement: S4 Fig — (A-D) When the death rate is non-zero, the system is bistable. Parameter values in panels (A-D) are δ = 0.01, p = 0.78, ϕ = 2, and γ = 0.0000562. (E, F) When the death rate is zero, the system is bimodal. Parameter values in panels (E, F) are δ = 0, p = 0.8, ϕ = 1.75, and γ = 0.005. The initial conditions in panels (E, F) are χ0(0) = 1 and χ1(0) = 0. (PDF) [file pcbi.1004814.s006.pdf]

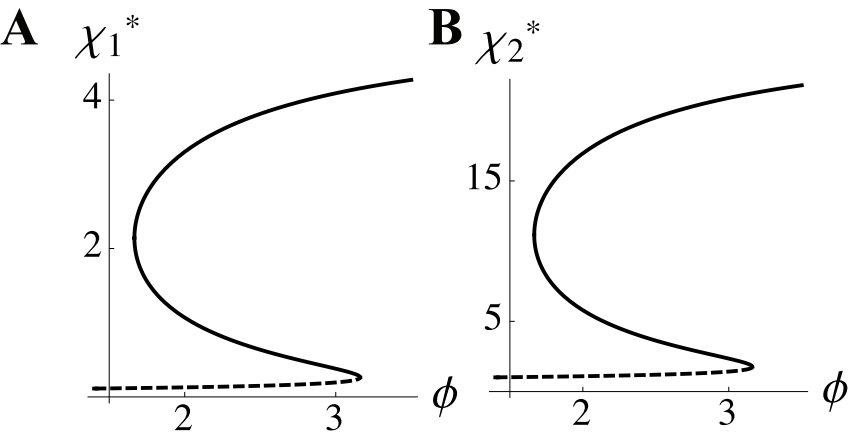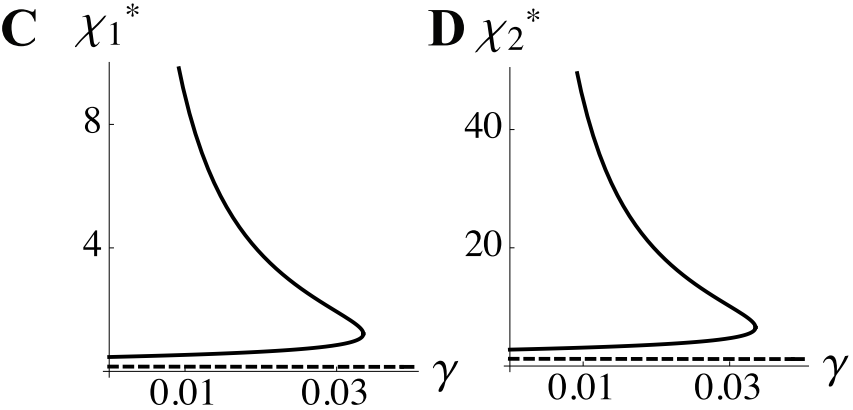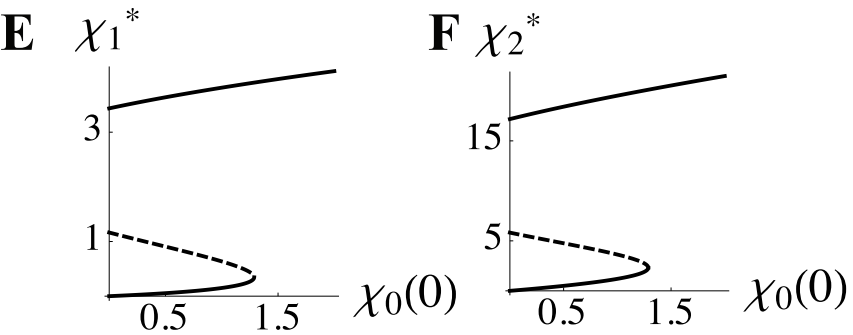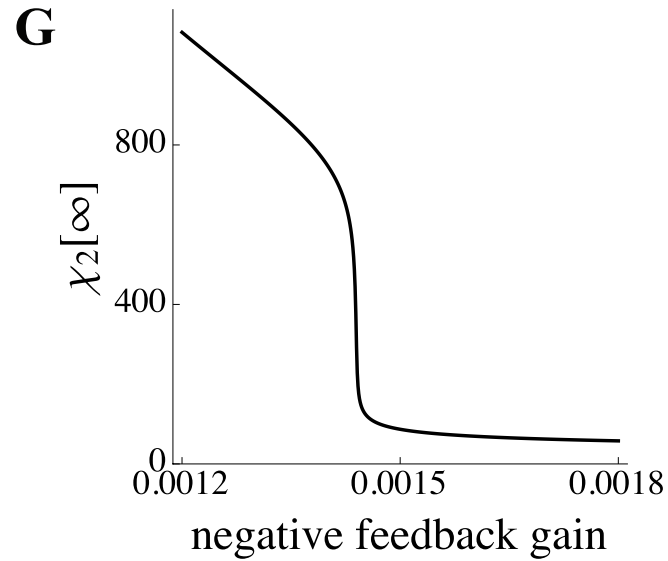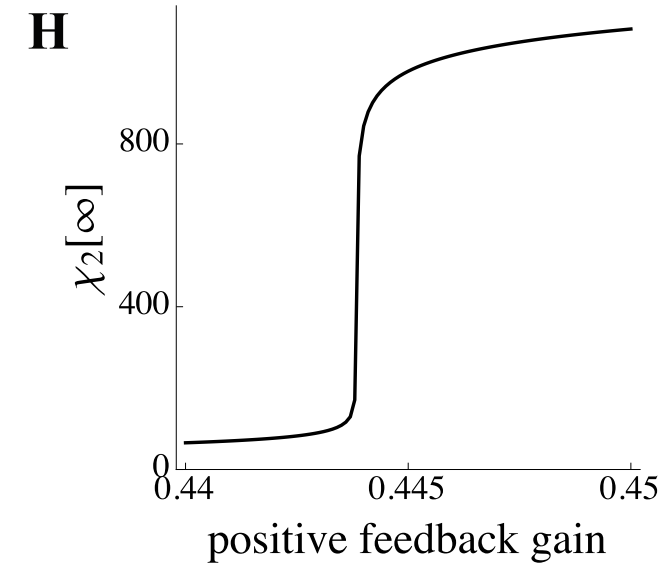

Supplement: S5 Fig — The CP and TD stages exhibit bistability with respect to positive feedback gain ϕ (A-B), negative feedback gain γ (C-D), and the stem cell population initial condition (E-F). Parameters are ζ = 0.09, δ = 0.2, p1 = 0.75, and ϕ = 2.5 or γ = 0.02, when not fixed. Growth bimodality is also observed if stem cell turnover is set to zero (p0 = 0) or made very slow. Panels (G) and (H) plot final state systems in which the stem cell mitosis rate and TD death are zero and p0 = 0, p1 = 0.8, and ϕ = 0.45 or γ = 0.0012, when not varied. Initial conditions are χ0(0) = 1, χ 1(0) = 4, and χ 2(0) = 8. (PDF) [file pcbi.1004814.s007.pdf]

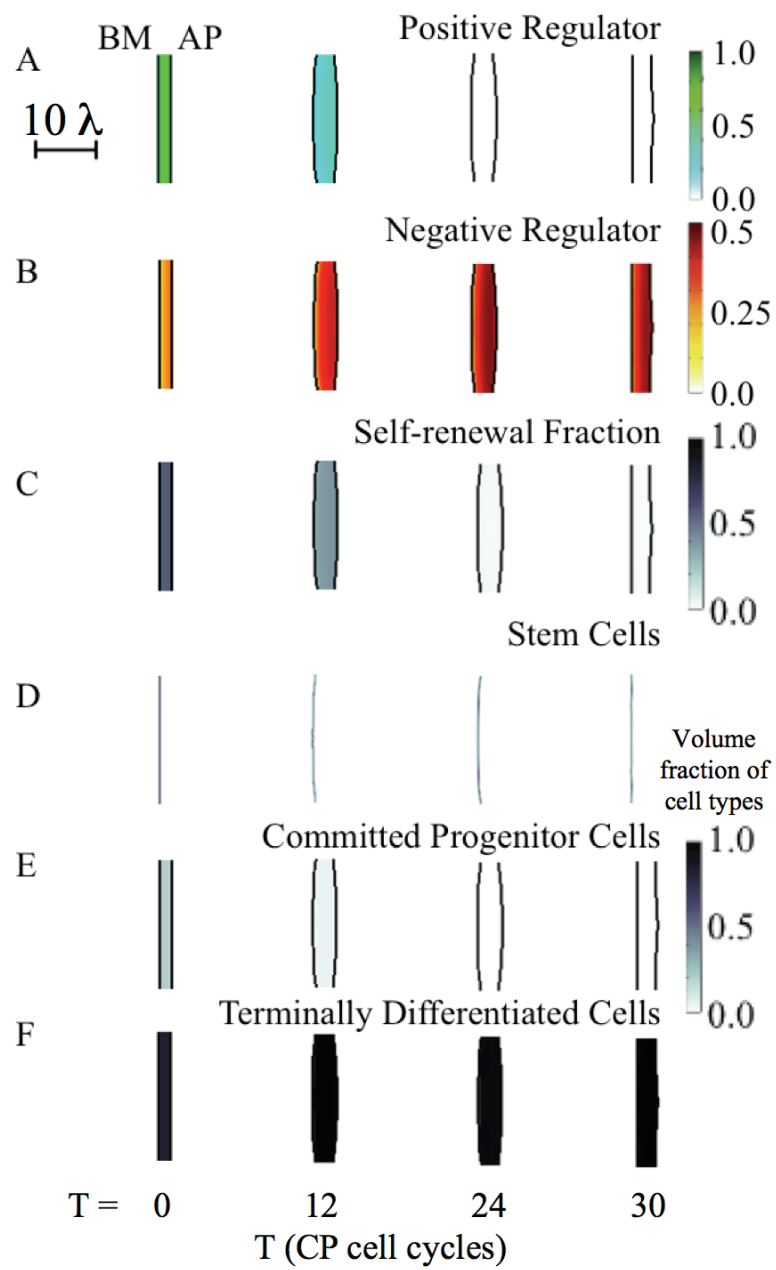

Supplement: S6 Fig — Evolution in time is shown for positive and negative feedback factors, the self-renewal fraction, and the spatial distribution of SCs, CPs and TDs. Panel (A) shows the distribution of positive feedback factors. Panel (B) shows the distribution of negative feedback factors. Panel (C) shows the distribution of the self-renewal fraction of CPs. The distributions of SCs are shown in (D), CPs are shown in (E) and TDs cells are shown in (F). λ is the diffusional length of feedback factor G. Although at early times the epithelium grows and cells stratify spatially within the epithelium, growth is not sustained, however, due the effects of negative feedback in the system. Endogenous sources of positive feedback factors are insufficient to sustain the self-renewal of CPs. CPs consequently differentiate into TDs and growth does not self-sustain itself. (PDF) [file pcbi.1004814.s008.pdf]

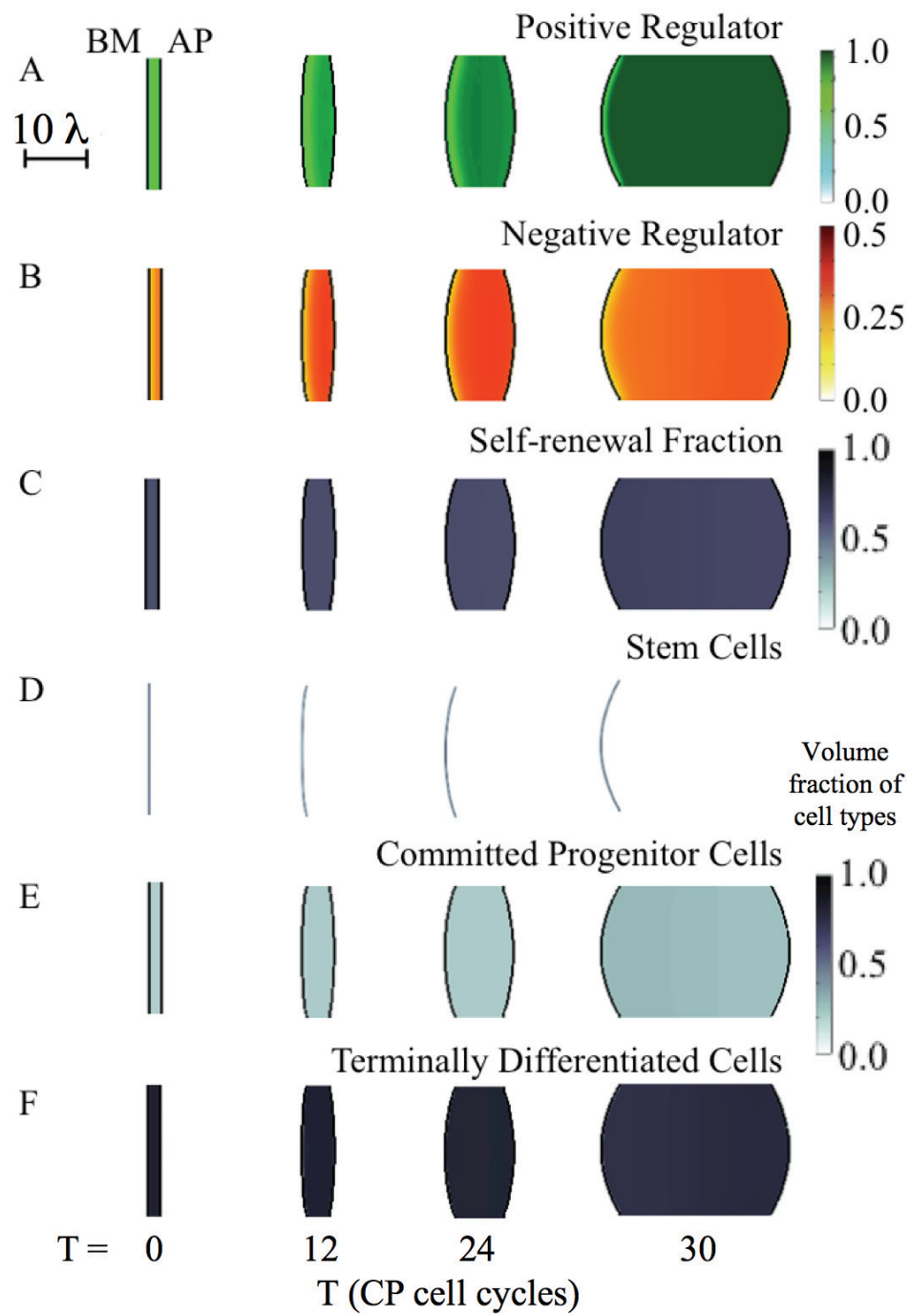

Supplement: S7 Fig — Evolution in time is shown for positive and negative feedback factors, the self-renewal fraction, and the spatial distribution of SCs, CPs and TDs. Panel (A) shows the distribution of positive feedback factors. Panel (B) shows the distribution of negative feedback factors. Panel (C) shows the distribution of the self-renewal fraction of CPs. The distributions of SCs are shown in (D), CPs are shown in (E) and TDs cells are shown in (F). λ is the diffusional length of feedback factor G. With this larger positive feedback gain of ϕ = 4.0, epithelial growth is self-sustained as CPs and TDs are distributed uniformly throughout the tissue. (PDF) [file pcbi.1004814.s009.pdf]

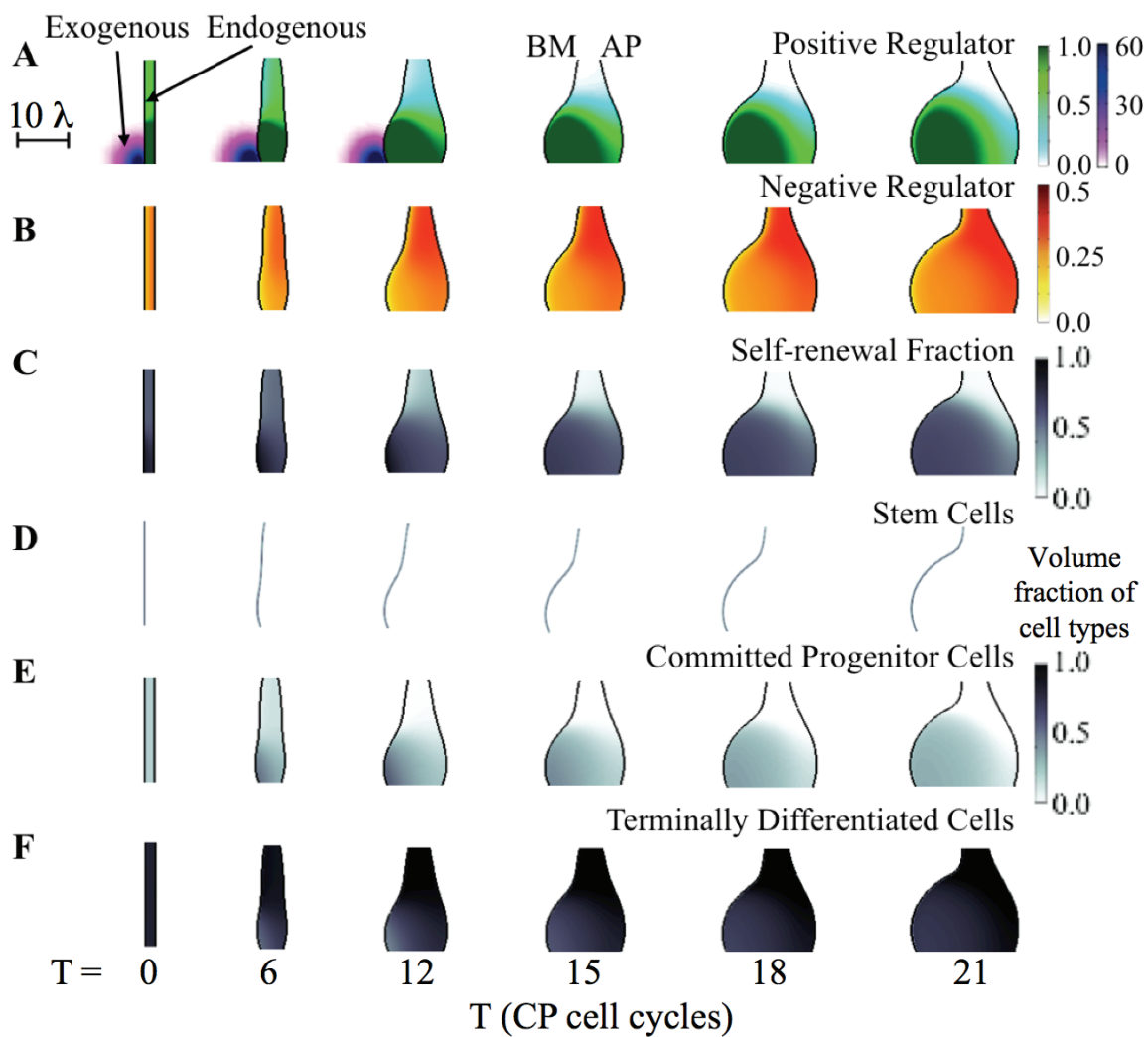

Supplement: S8 Fig — CPs produce negative feedback factors at a rate of 0.3, while the production rate of the negative feedback factors by TD cells is decreased from 0.5 (when TD cells were the only source of a negative regulator) to 0.35. TD cell production of negative feedback is set to a lower rate in order to keep the overall level of negative feedback at roughly the same level as before. The distributions of the positive and negative factors are shown in (A) and (B), respectively. The self-renewal fraction of CPs is shown in (C). In (D—F), the distributions of CPs and TDs are shown. λ is the diffusional length of feedback factor G. CPs are less concentrated at the BM, because CPs produce negative feedback factors that promote differentiation. As a result, the epithelium is less stratified than the epithelium simulated in Fig 4 in the main text. (PDF) [file pcbi.1004814.s010.pdf]

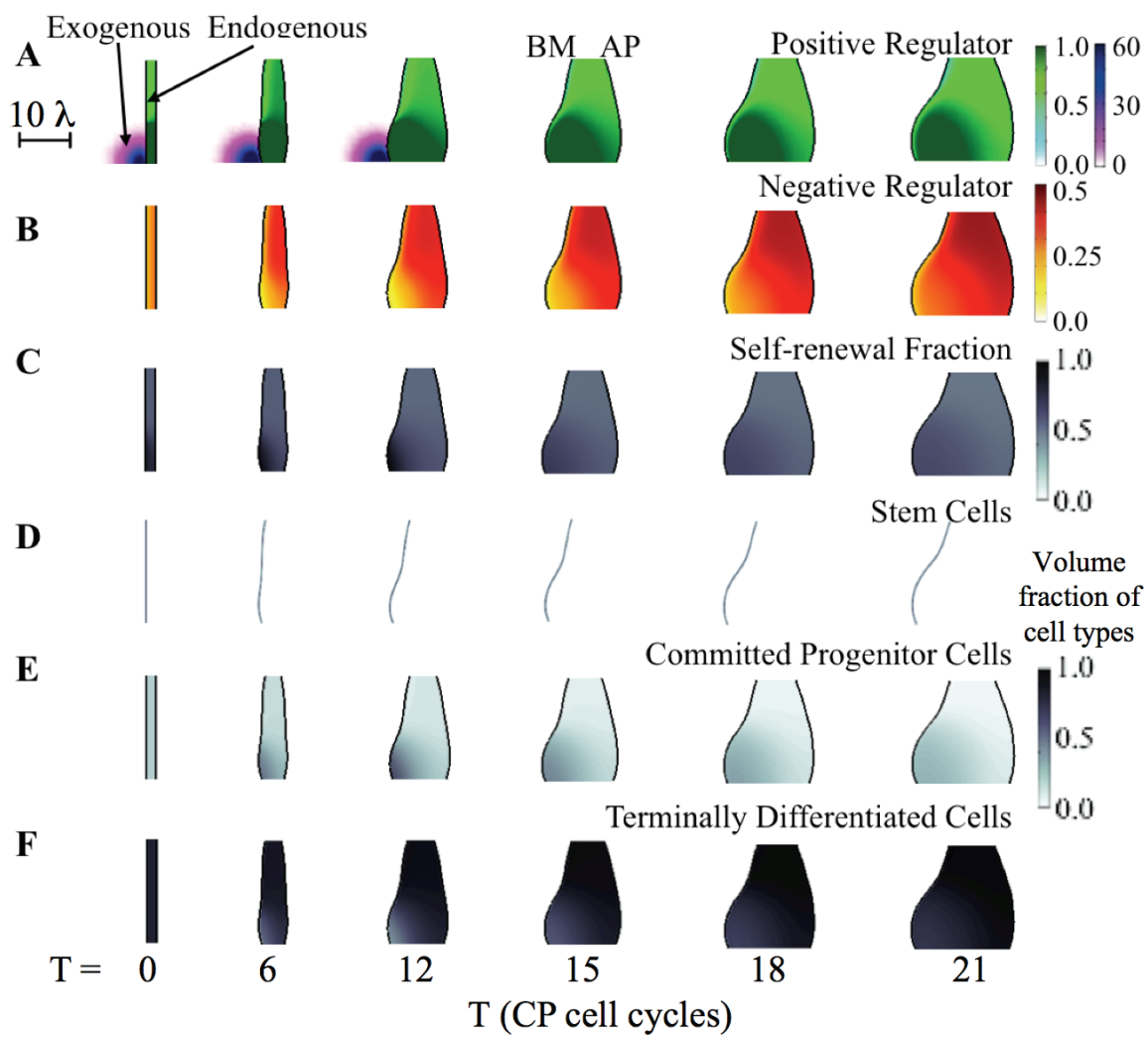

Supplement: S9 Fig — TD cells produce positive feedback factors at a rate of 0.5, while the production rate of positive feedback factor by the CPs is decreased from 5.0 (when CPs were the only source of a positive feedback) to 4.0, in order to compensate for an excess of positive feedback. CPs production of negative feedback is set to a lower rate in order to keep the overall level of negative feedback at roughly the same level as before. The distributions of the positive and negative factors are shown in (A) and (B), respectively. The self-renewal fraction of CPs is shown in (C). In (D—F), the distributions of CPs and TDs are shown. λ is the diffusional length of feedback factor G. CPs are concentrated at the BM but also appear near the AP because the positive feedback factors produced by TDs sustain high CP self-renewal. However, the amount of endogenous positive feedback is insufficient to elevate CP self-renewal above 0.5, so most CPs near the AP still differentiate into TDs. As a result, the epithelium remains spatially stratified. (PDF) [file pcbi.1004814.s011.pdf]

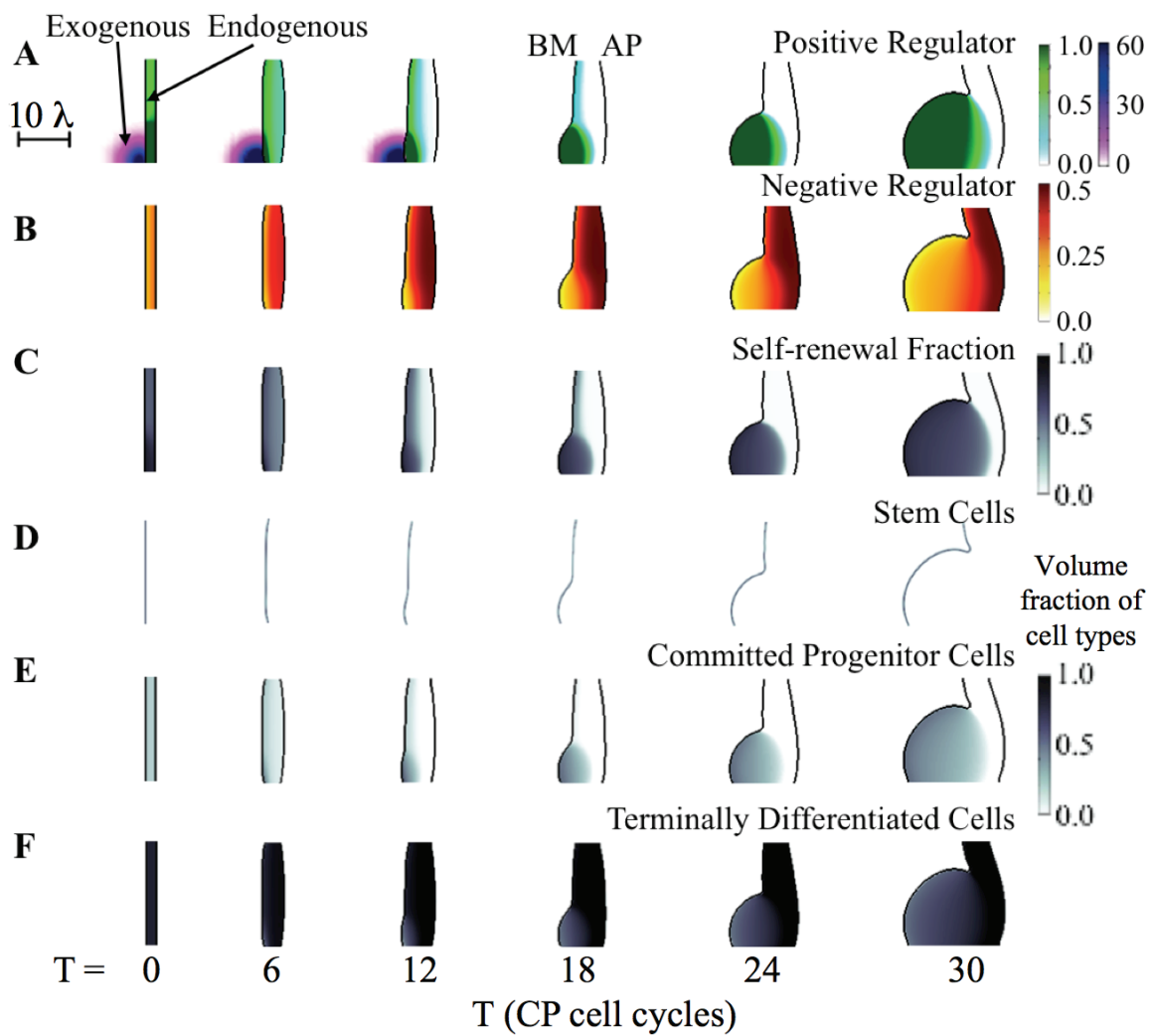

Supplement: S10 Fig — In the main text, all of the presented spatial simulations have diffusivities of positive feedback factors set as equal (DF = DG = 1.0). Here a similar epithelial simulation is achieved with DF = 0.01 and a slightly smaller production rate of the positive feedback factor F. A transient exogenous source of feedback factors is applied up to T = 12 as in Fig 5A in the main text. The distributions of the positive and negative factors are shown in (A) and (B), respectively. The self-renewal fraction of CPs is shown in (C). In (D—E), the distributions of CPs and TDs are shown. λ is the diffusional length of feedback factor G. The application of positive regulator by an exogenous source is sufficient to ignite self-sustained, spatially stratified growth, although positive feedback factor F is more sharply stratified across the epithelium. (PDF) [file pcbi.1004814.s012.pdf]

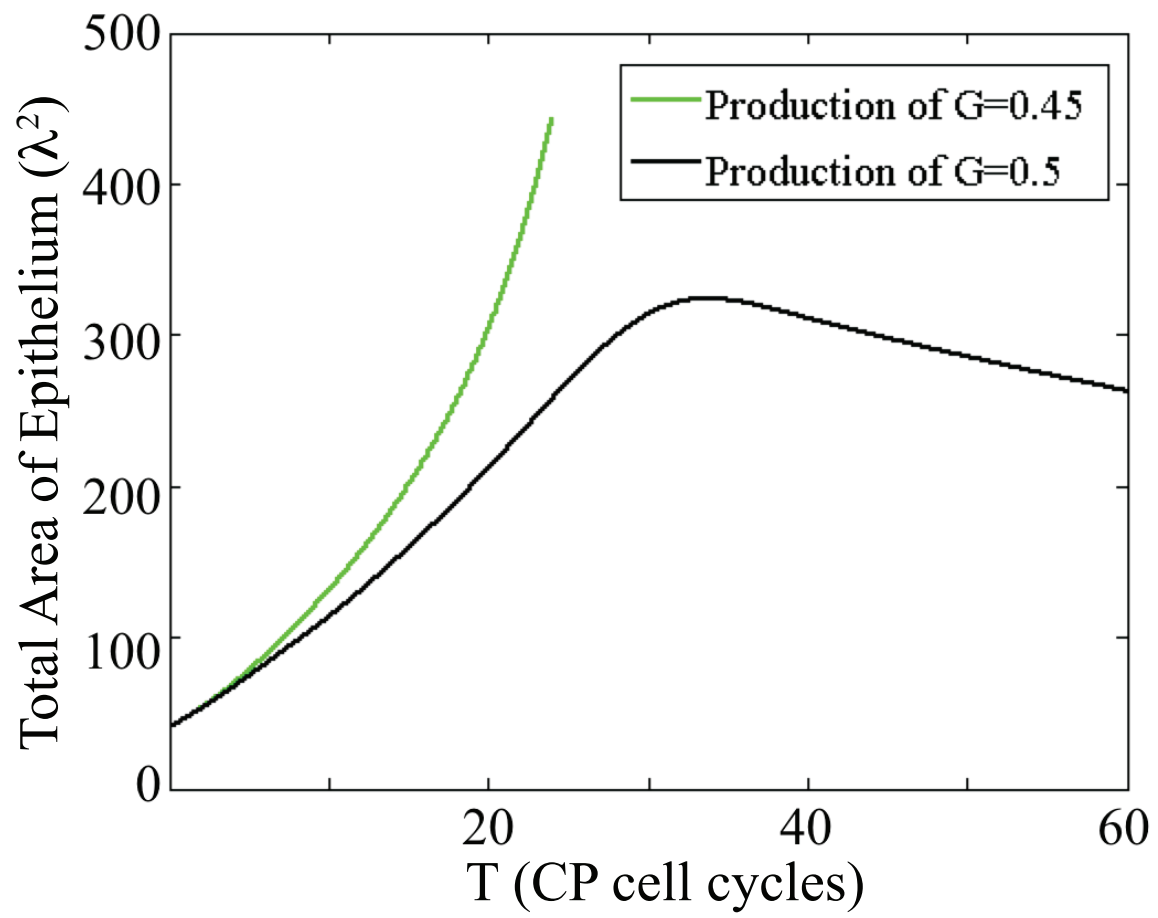

Supplement: S11 Fig — The area of total epithelium is plotted as a function of time. In the main text, negative feedback factors are assumed to be bound to the stroma by Follistatin (FST); this binding is modeled by a sink term in the equations. Here we show that similar results can be obtained if this sink is turned off. Specifically, if this sink is simply turned off, the growth of the epithelium is no longer sustained (as indicated by the black curve), but if the production rate of negative feedback factor from TDs is decreased from 0.5 to 0.45, growth does self-sustain (as indicated by the green curve). (PDF) [file pcbi.1004814.s013.pdf]

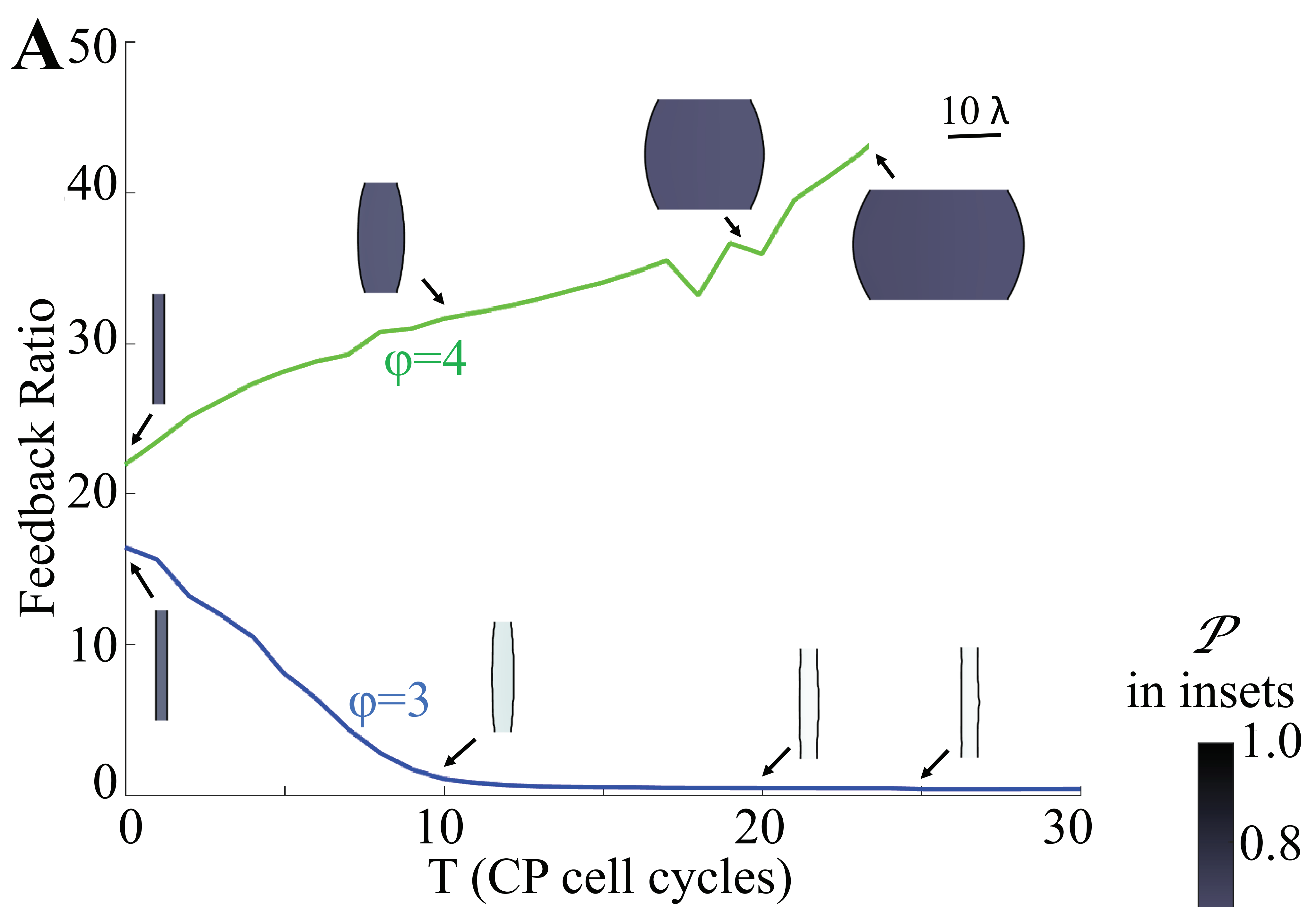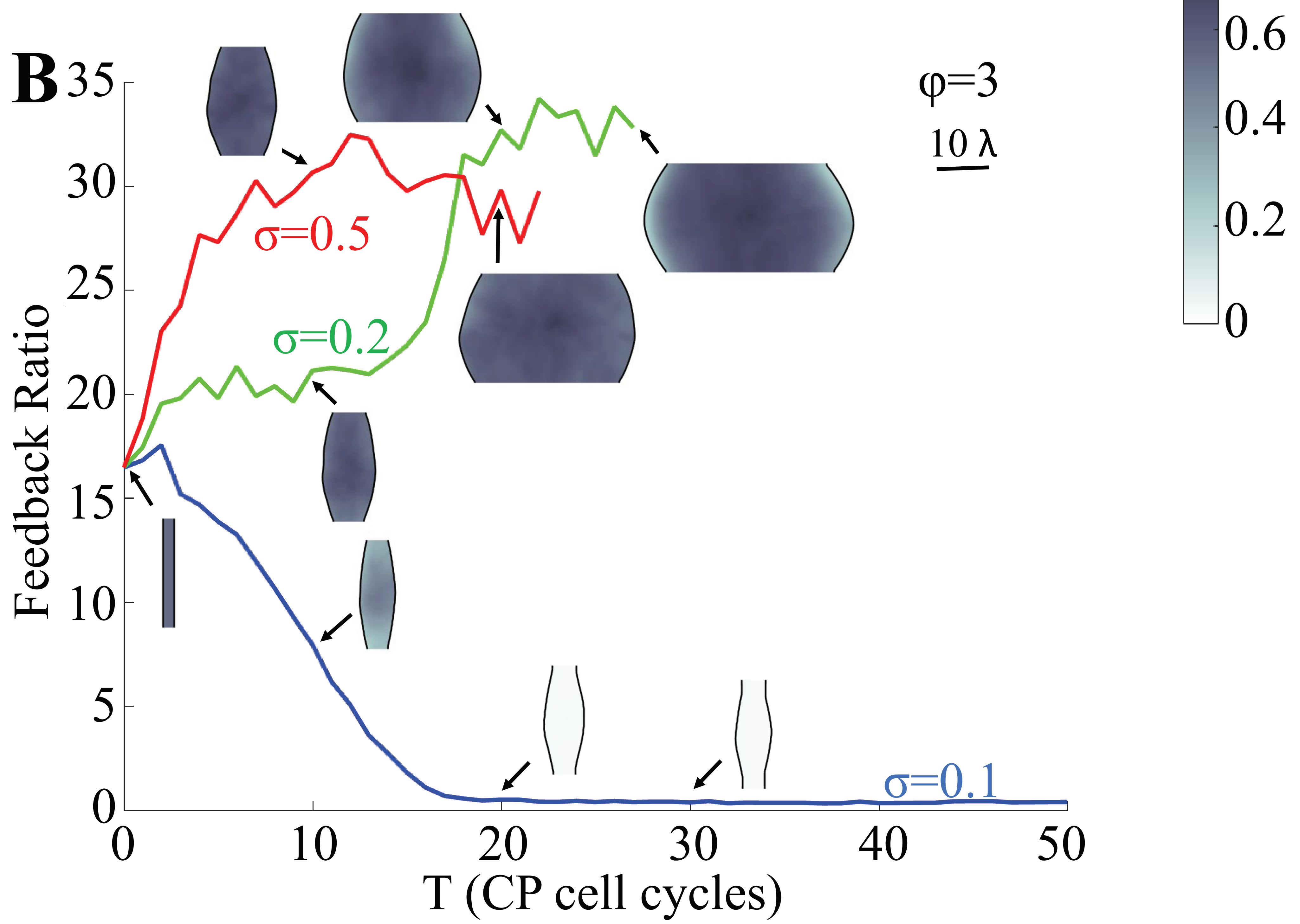

Supplement: S12 Fig — A comparison of the feedback ratios using the diffusing positive and negative feedback signals. The feedback ratios are plotted as functions of time (measured in CP cell cycles), as they develop spatially for different positive feedback strengths, ϕ/γ. The ratio is calculated as max ϕ [F] / (γ [G] + ε), where ε = 10−3 is a small number, [F] and [G] are the concentrations of the positive and negative feedback regulators, and the maximum is taken within the epithelium. (A) Deterministic growth with positive feedback gains of ϕ = 3.0 and ϕ = 4.0. The feedback ratio is shown as functions of time and the distributions of CP self-renewal at the 0th, 12th and 24th CP cell cycles are shown as insets. Growth is not self-sustaining when ϕ = 3.0 because the positive feedback produced by the CPs is insufficient to sustain CP self-renewal (green curve). The epithelium with ϕ = 4.0 grows similarly at early times and continues to grow exponentially at later times driven by positive feedback on CP self-renewal. CPs produce enough positive feedback factor to drive the ratio of positive-to-negative feedback over a critical threshold for self-sustaining growth (blue curve). (B) Growth with stochastic p, as in Fig 3 of the main text. The positive feedback gain is ϕ = 3.0. With small variance (0.1), growth is not self-sustaining as in panel A. With larger variance, the growth is self-sustaining as the randomness increases the feedback ratio above the critical threshold. Larger variance results in faster growth. (PDF) [file pcbi.1004814.s014.pdf]

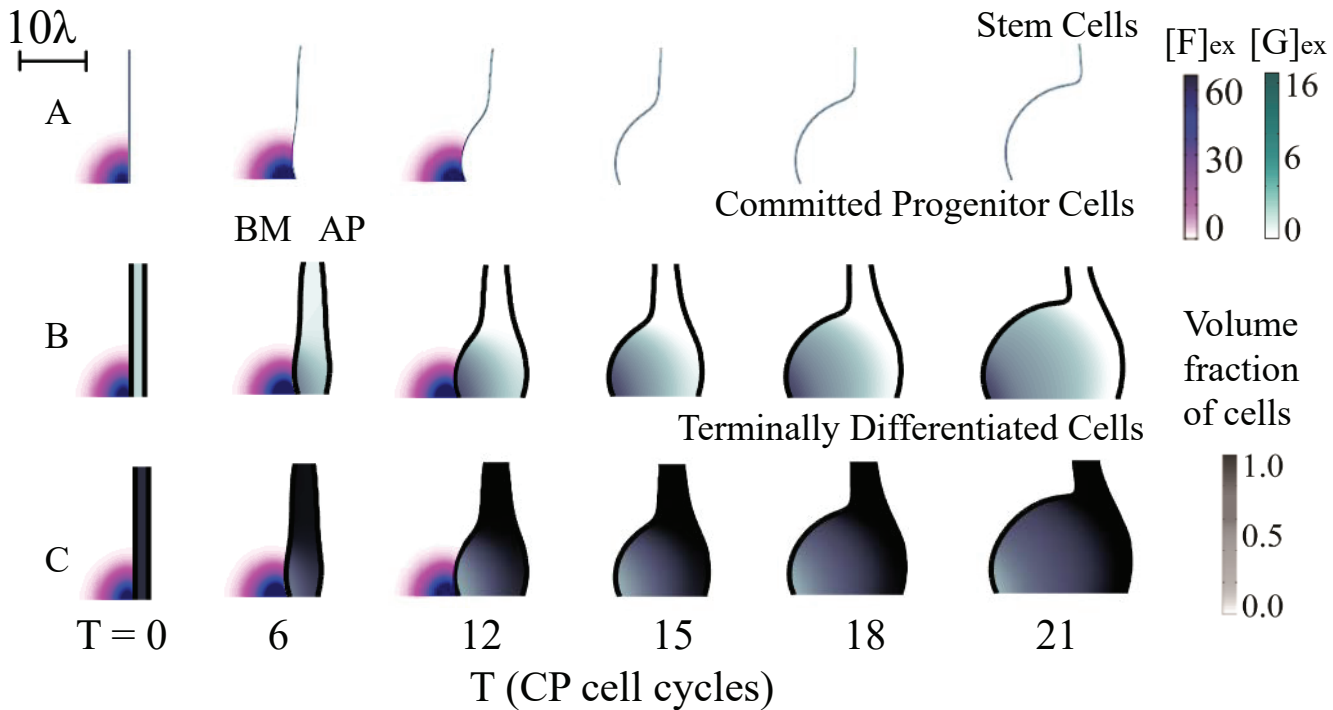

Supplement: S13 Fig — λ is the diffusional length of feedback factor G. The positive regulator is produced exogenously from a localized source until time T = 12. Growth self-sustains within a region of the epithelium near the positive regulator’s source, and cell distributions spatially stratify such that more CPs are located in the growing region while more TDs are located in the non-growing region. Both self-sustained growth and stratification occur both during application of the exogenous signal and after removal of the exogenous source. (PDF) [file pcbi.1004814.s015.pdf]

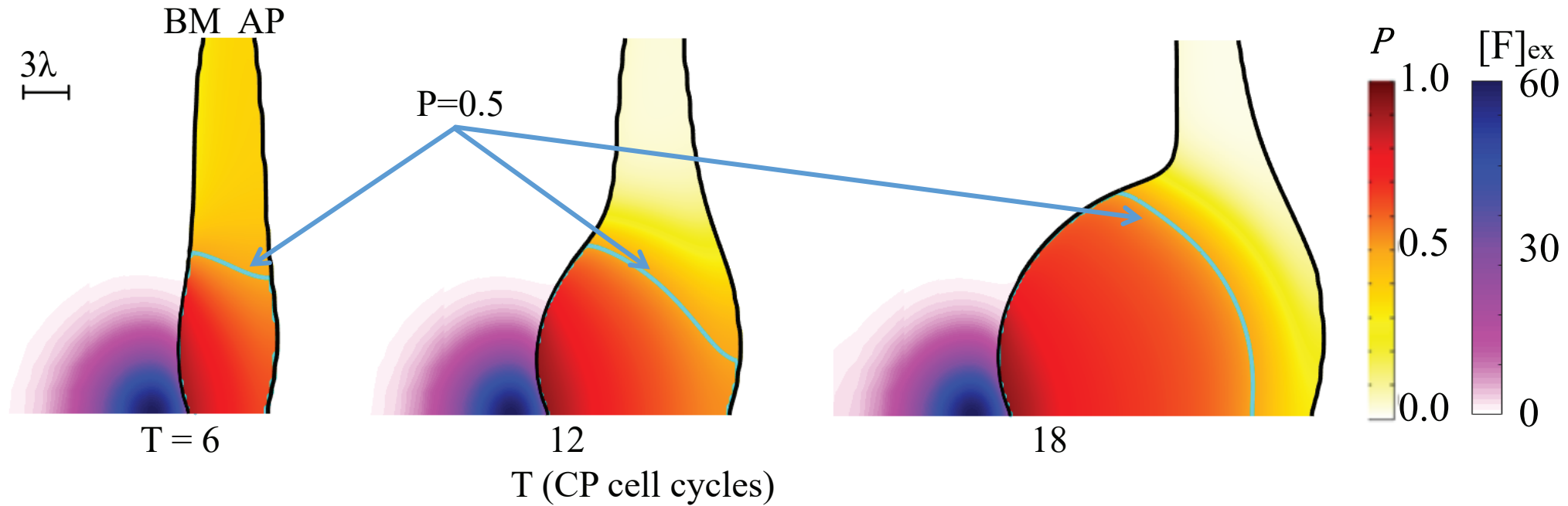

Supplement: S14 Fig — λ is the diffusional length of feedback factor G. The positive regulator is produced exogenously from a localized source until time T = 12. A green contour indicates the epithelial interface, and a cyan contour represents where P = 0.5. An exogenous regulator causes an area near the regulator’s source to take on high P values. The CP population therefore is primarily undergoing self-renewal in the region of self-sustaining growth. (PDF) [file pcbi.1004814.s016.pdf]

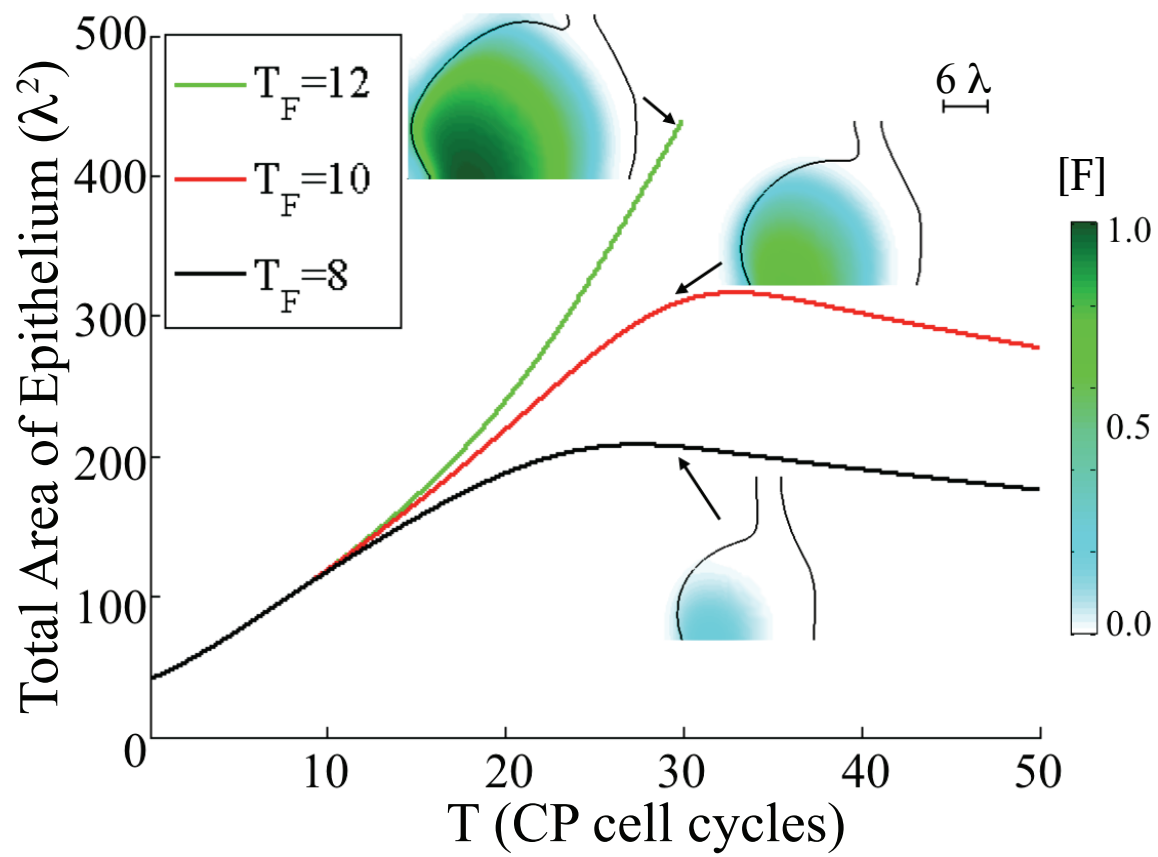

Supplement: S15 Fig — Total areas of the epithelium are shown as functions of time. λ is the diffusional length of feedback factor G. The exogenous source of positive regulator is removed at TF = 8, 10 and 12, as labeled. Epithelial growth does not self-sustain if the exogenous source of positive regulator is removed at T = 8 or T = 10. However, when the source is removed at T = 12, growth self-sustains and the area of the total epithelium continues to increase. Insets show the morphologies of the epithelia and the spatial distribution of positive feedback factors at T = 30. (PDF) [file pcbi.1004814.s017.pdf]

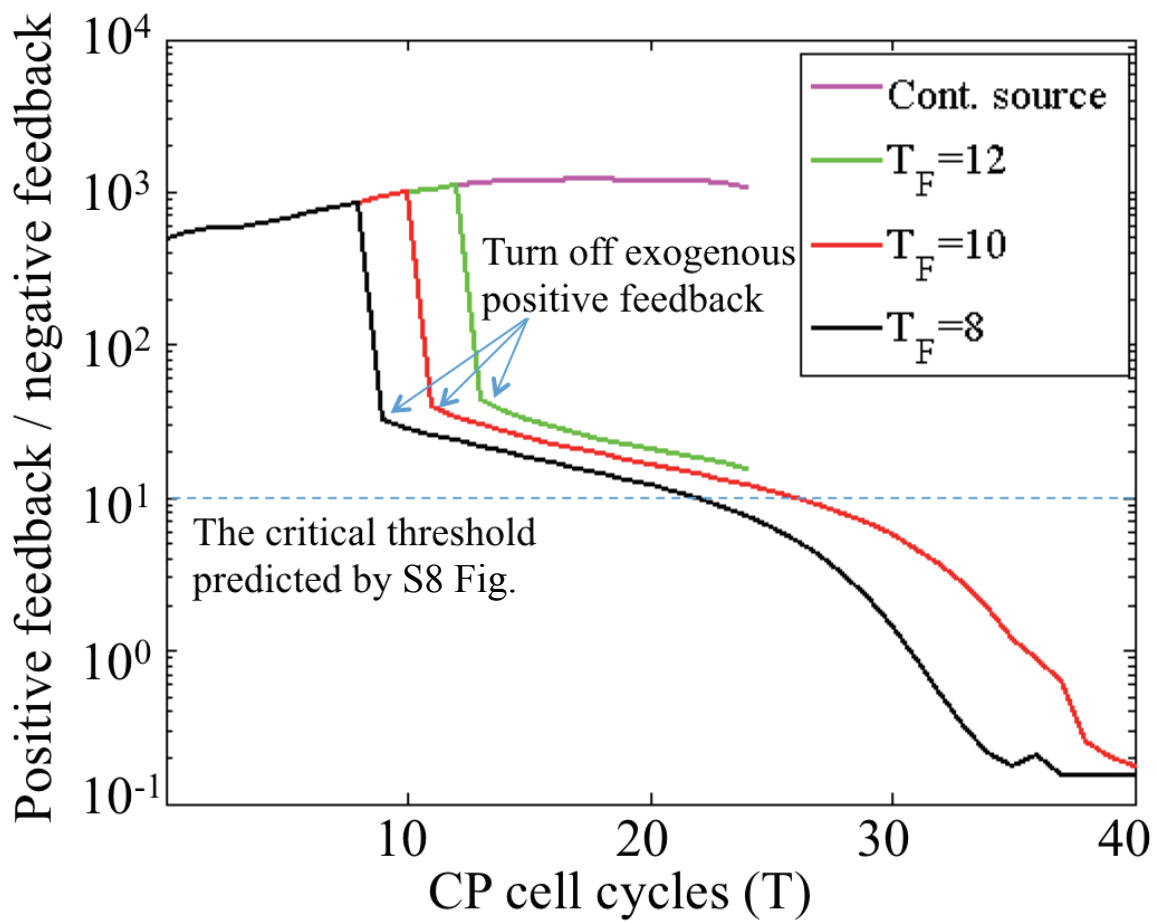

Supplement: S16 Fig — The ratio is calculated as maxBMϕ[F]/(γ[G]+ε), where ε = 10−3 and the maximum is taken along the BM. The critical threshold estimated in S12 Fig is marked by the dashed line. The exogenous source of positive feedback is removed at TF = 8, 10 and 12 respectively. When the exogenous source is removed, the ratio drops due to the removal of positive feedback. If the source is removed at TF = 8 (black curve) or TF = 10 (red curve), the ratio steadily decreases below the critical threshold, and growth is not sustained. However, if the source is removed at TF = 12 (green) or is applied continuously (magenta), the ratio stays above the critical threshold for the duration of the simulation. The epithelium corresponding to the green curve grows out of the computational domain before T = 30, and therefore its presented trajectory in the figure is not as long as the others. (PDF) [file pcbi.1004814.s018.pdf]

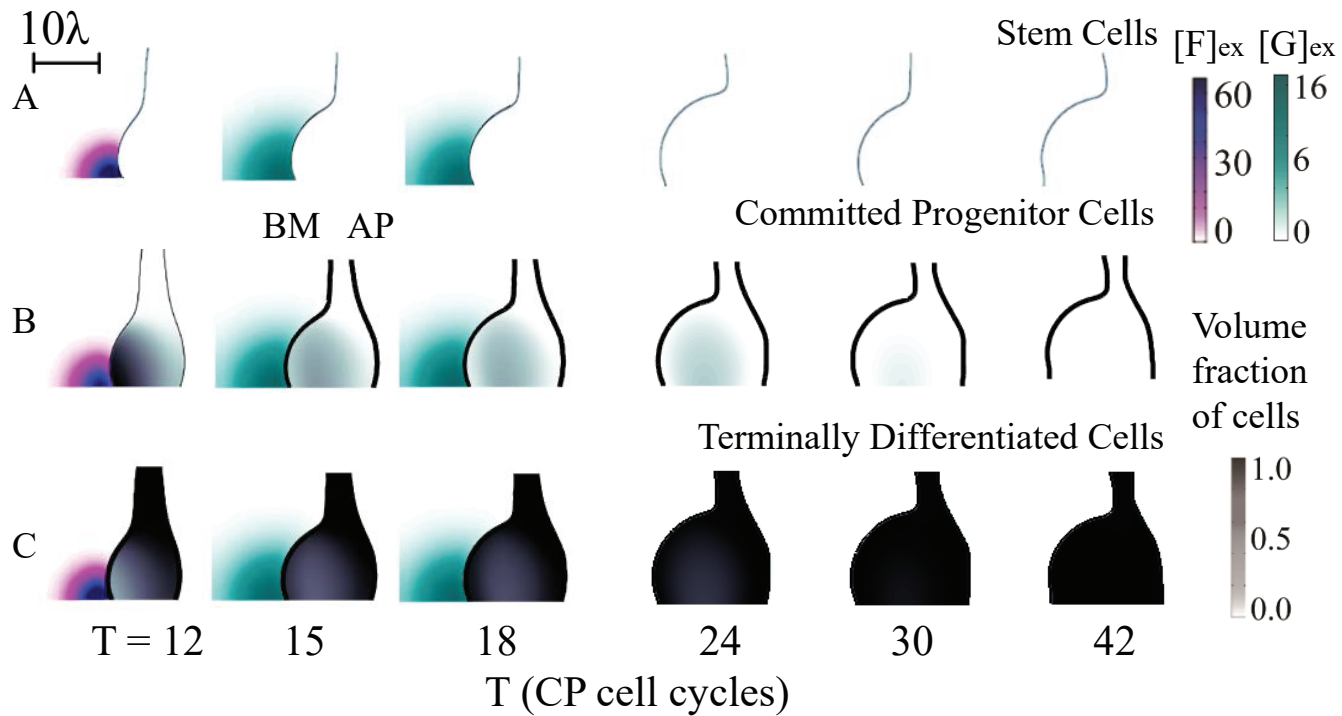

Supplement: S17 Fig — The development in time of the spatial distributions of SCs, CPs and TDs are presented here. λ is the diffusional length of feedback factor G. Until time T = 15, the simulation is the same as in S13 Fig, but from time T = 15 to T = 18, a negative regulator is applied exogenously. Growth is subsequently terminated even after the negative regulator is removed, and the epithelium is comprised primarily of TDs at late times. The evolution of feedback factors and the self-renewal fraction of CPs maybe found in Fig 5B in the main text. The spatial stratification seen up until T = 15 is lost afterwards as CPs differentiate into TDs. (PDF) [file pcbi.1004814.s019.pdf]

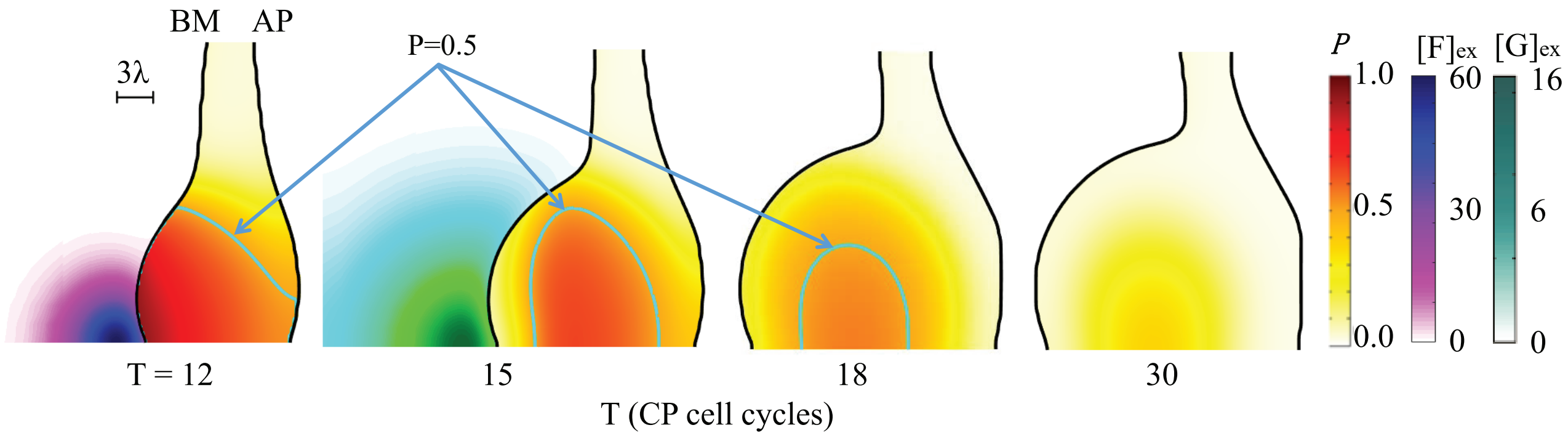

Supplement: S18 Fig — λ is the diffusional length of feedback factor G. Until time T = 15, the simulation is the same as in S13 Fig, but from time T = 15 to T = 18, a negative regulator is applied exogenously. A cyan contour represents where P = 0.5. The exogenous application of negative regulator drives P below 0.5, which causes CPs to differentiate. A reduction in CPs reduces endogenous positive feedback, and growth ceases after self-renewal in CPs sufficiently declines. (PDF) [file pcbi.1004814.s020.pdf]

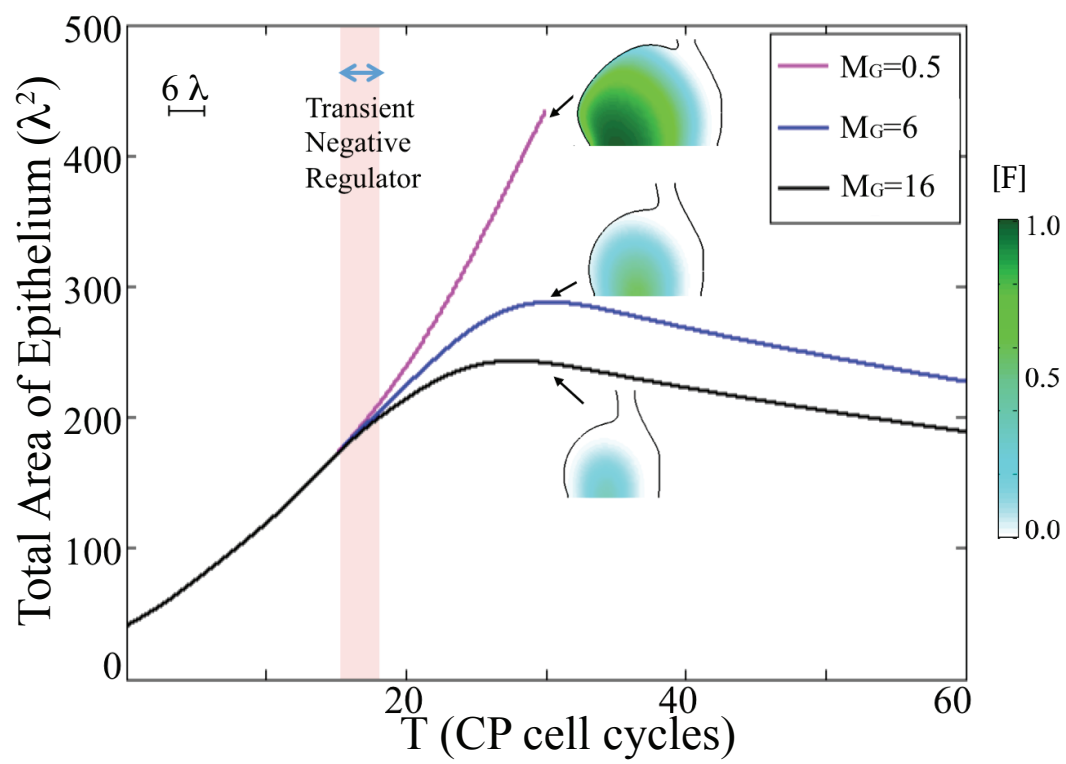

Supplement: S19 Fig — The signal strengths are indicated by peak concentrations of the negative regulator, which are MG = 0.5, 6, and 16. The area of the total epithelium is plotted as a function of time. λ is the diffusional length of the feedback factor G. From T = 15 to T = 18, a exogenous negative regulator is applied. When the peak concentration of the exogenous negative feedback factors is 16.0 (black) or 6.0 (blue), the area of the total epithelium increases transiently and then steadily drops, indicating that the growth is not self-sustaining. When the peak is 0.5 (magenta), the area steadily increases, indicating that growth is still self-sustained. Insets show the spatial distribution of positive feedback factors at T = 24. (PDF) [file pcbi.1004814.s021.pdf]

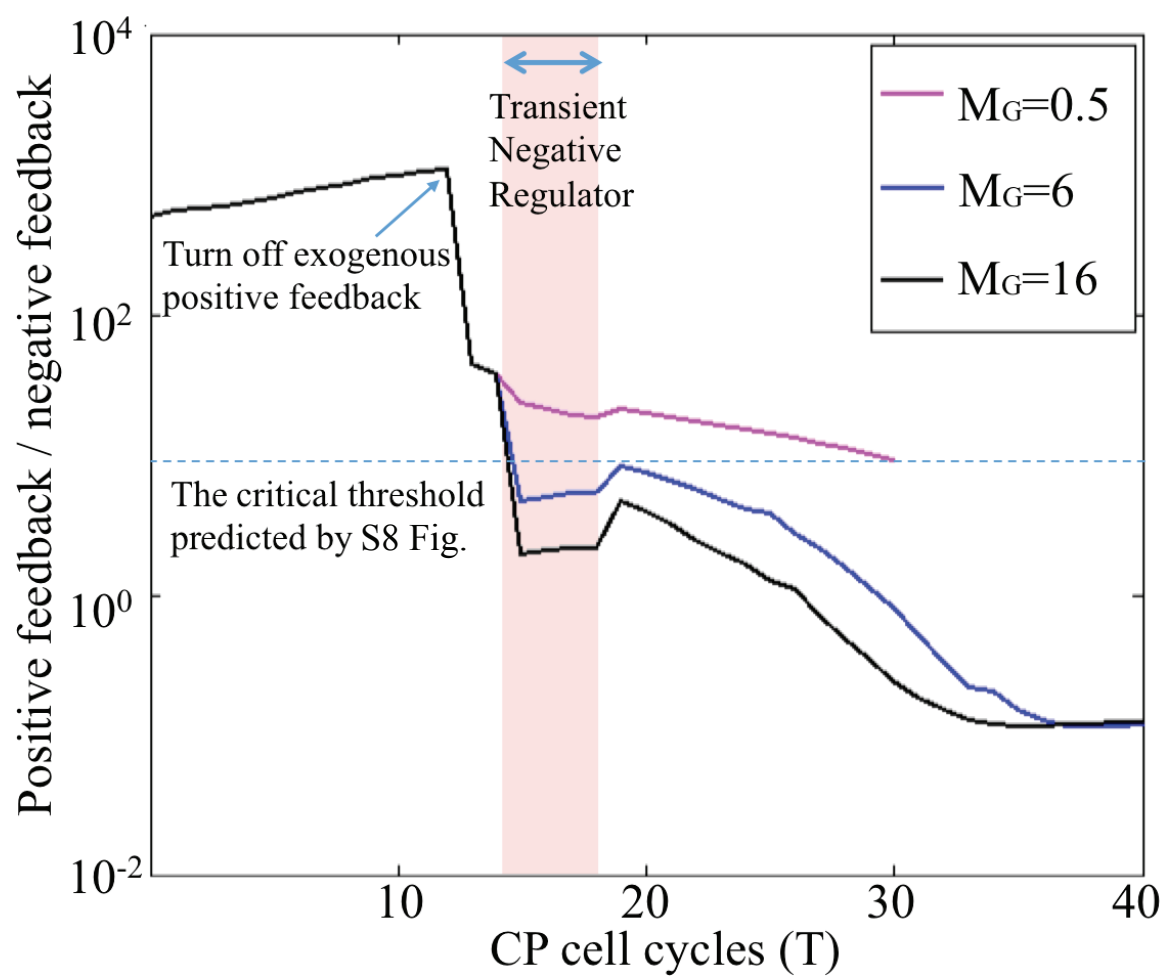

Supplement: S20 Fig — The ratio is calculated as maxBMϕ[F]/(γ[G]+ε), where ε = 10−3 and the maximum is taken along the BM. The critical threshold predicted by S12 Fig is marked by the dashed line. When the exogenous source of positive feedback factors is removed at T = 12, the ratio immediately decreases. At T = 15, the ratio drops again because exogenous negative feedback is applied. When the peak concentration of the exogenous negative feedback factors is 16.0 (black) or 6.0 (blue), the ratio drops because most CPs differentiate, and growth is subsequently extinguished. When the peak concentration of the exogenous negative feedback factors is 0.5 (magenta), the ratio stays above the critical threshold after the source is removed, indicating that the growth is sustained (see also S19 Fig). (PDF) [file pcbi.1004814.s022.pdf]

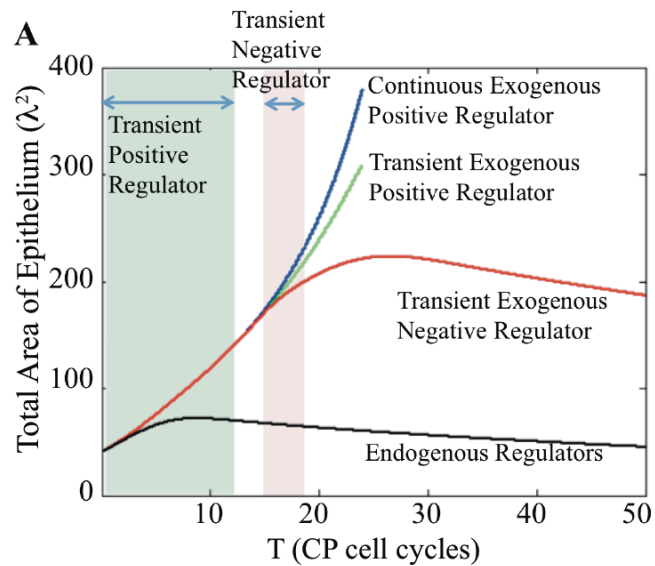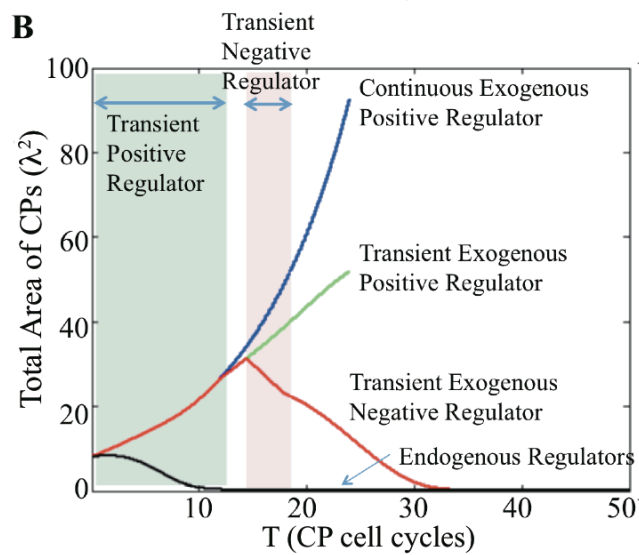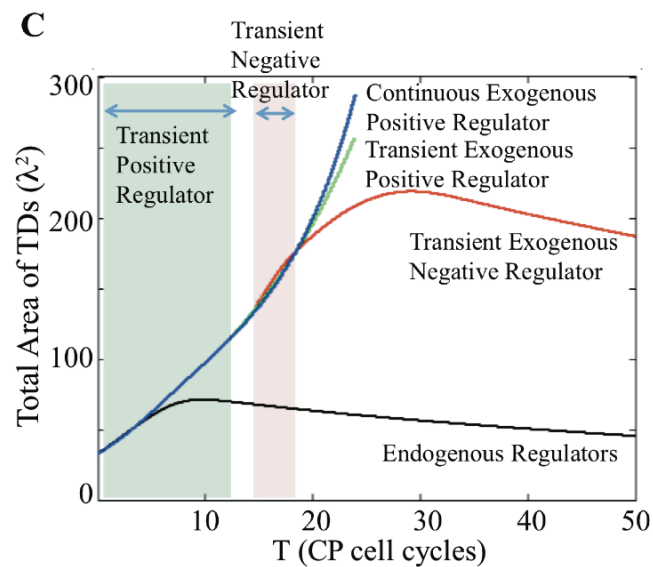

Supplement: S21 Fig — In (A), the total areas of the epithelium from Fig 3E (black; no exogenous signaling factors), from Fig 4 (blue; exogenous source of positive feedback factors), from Fig 5A (green; transient exogenous source of positive feedback factors), and Fig 5B (red; transient exogenous source of negative feedback factors) are shown. In (B) and (C) the corresponding areas of the CPs and TDs, respectively, are shown. Note that the area of stem cells is conserved in all cases. (PDF) [file pcbi.1004814.s023.pdf]

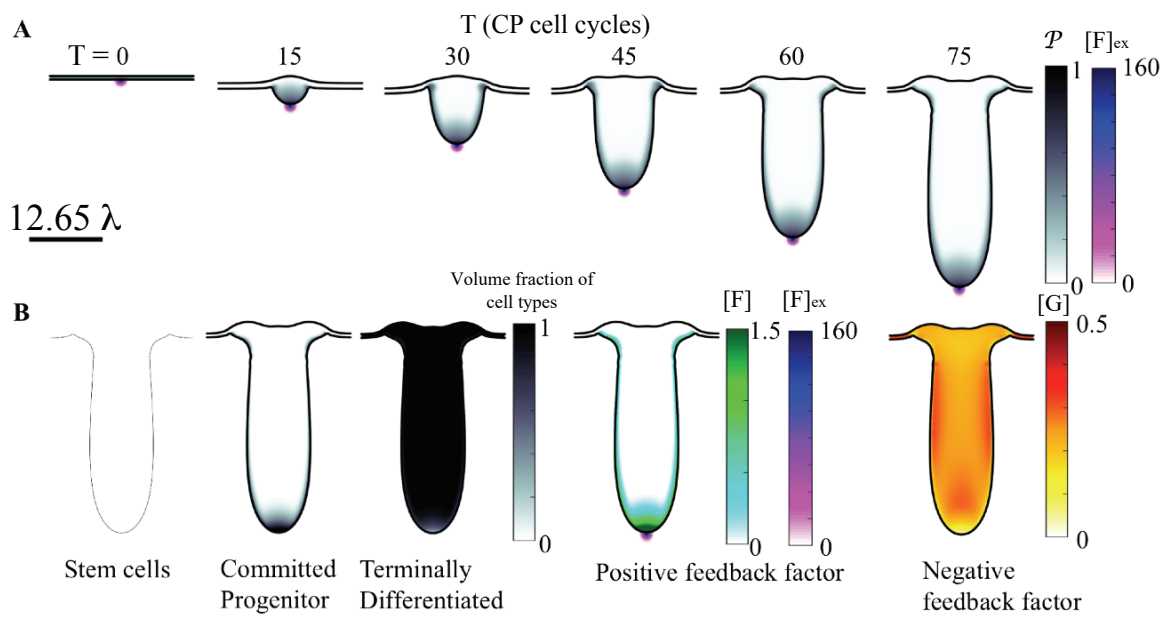

Supplement: S22 Fig — λ is the diffusional length of negative feedback factor G. The black contour indicates the epithelial interface. Panel (A) shows the time evolution of the CP self-renewal ratio, P, (indicated by the gray scale gradient) upon application of an exogenous positive regulator (indicated by a blue-purple gradient). Panel (B) shows the spatial distributions of cell components, positive regulation factors (where the green-cyan gradient indicates the concentration of endogenous feedback and the blue-purple gradient indicates the exogenous regulator’s concentration) and negative regulators (indicated by the red-yellow gradient) at T = 75. (PDF) [file pcbi.1004814.s024.pdf]

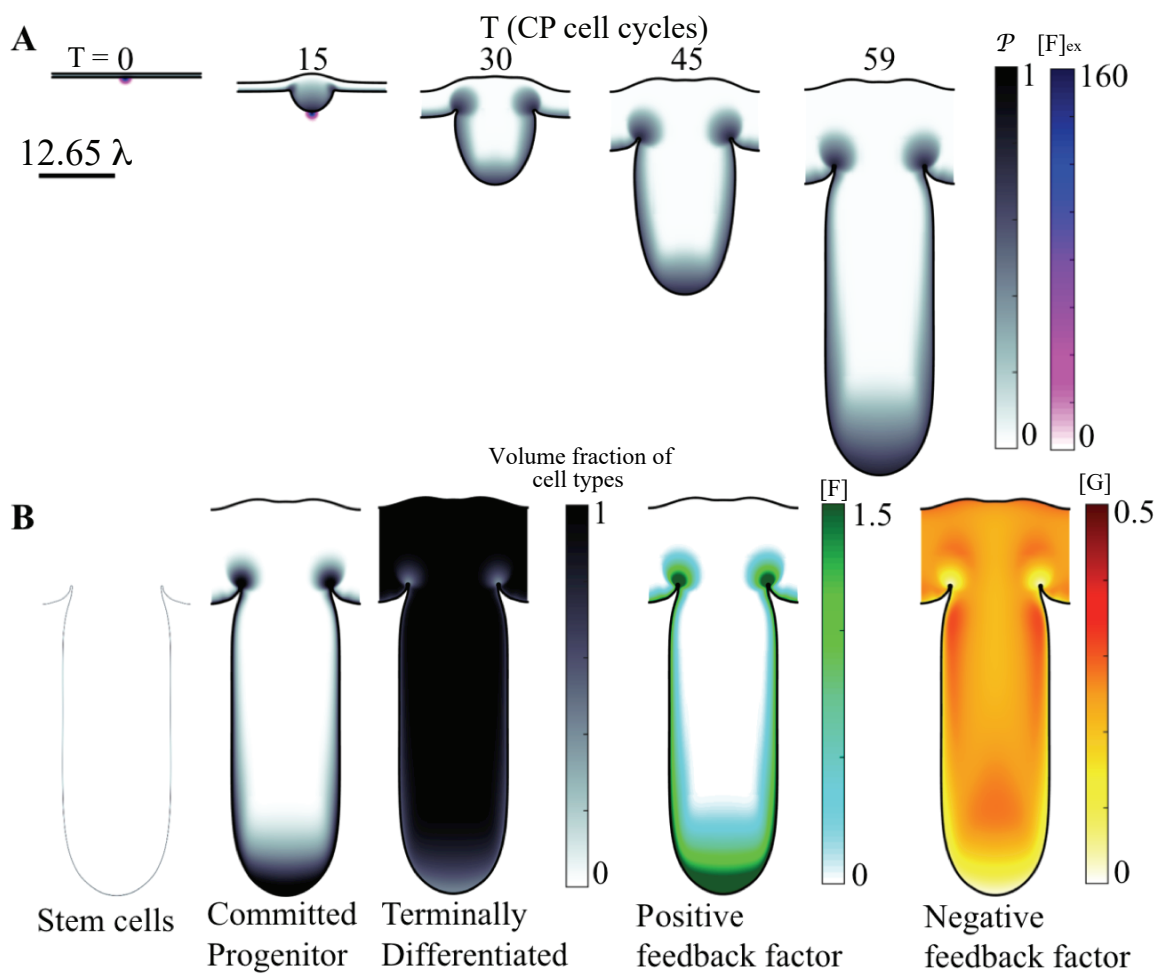

Supplement: S23 Fig — λ is the diffusional length of negative feedback factor G. The black contour indicates the epithelial interface. Panel (A) shows the time evolution of the CP self-renewal ratio, P, (indicated by the gray scale gradient) upon application of an exogenous positive regulator (indicated by a blue-purple gradient). Panel (B) shows the spatial distributions of cell components, positive regulation factors (where the green-cyan gradient indicates the concentration of endogenous feedback and the blue-purple gradient indicates the exogenous regulator’s concentration) and negative regulators (indicated by the red-yellow gradient) at T = 59. (PDF) [file pcbi.1004814.s025.pdf]

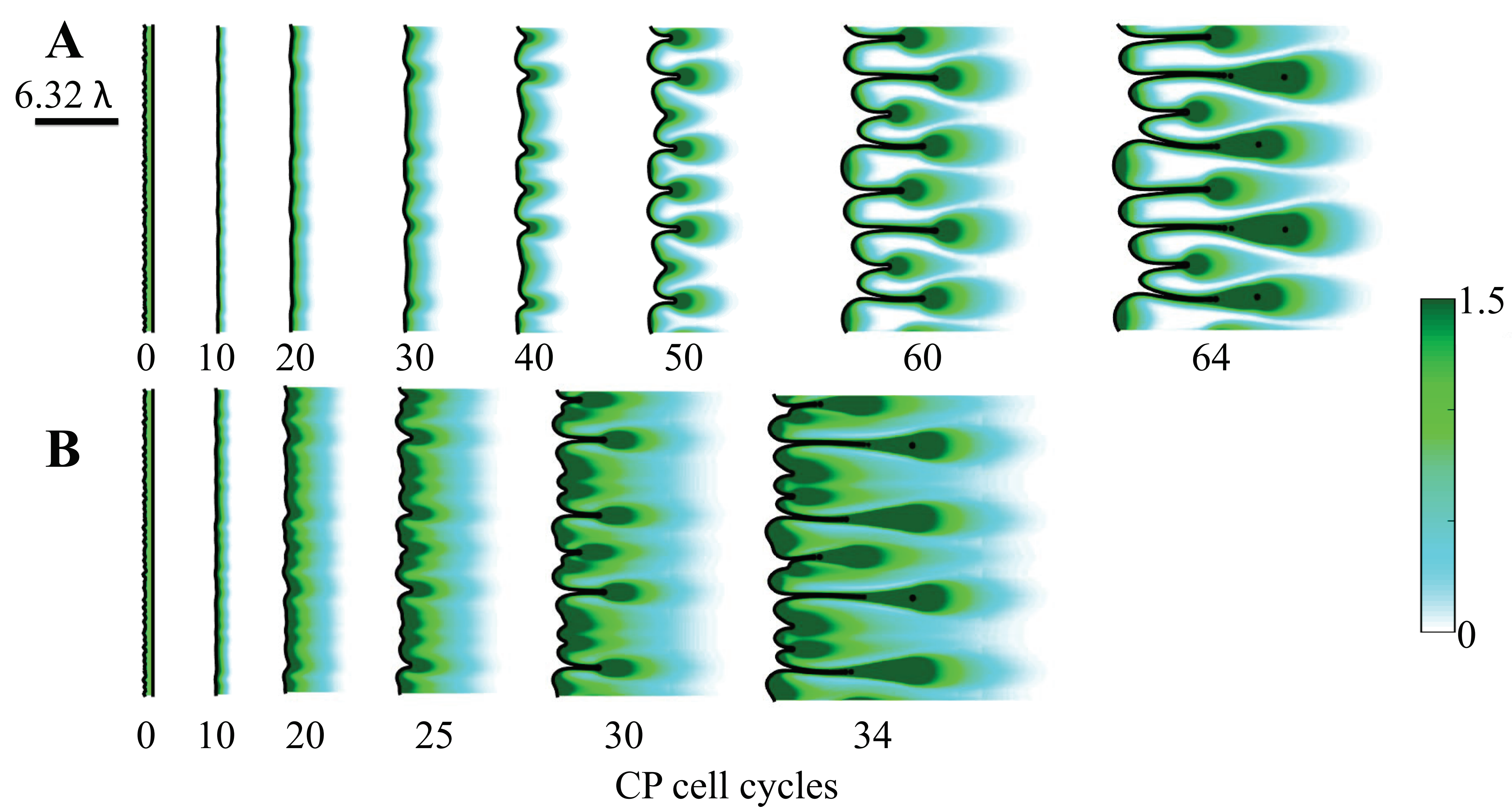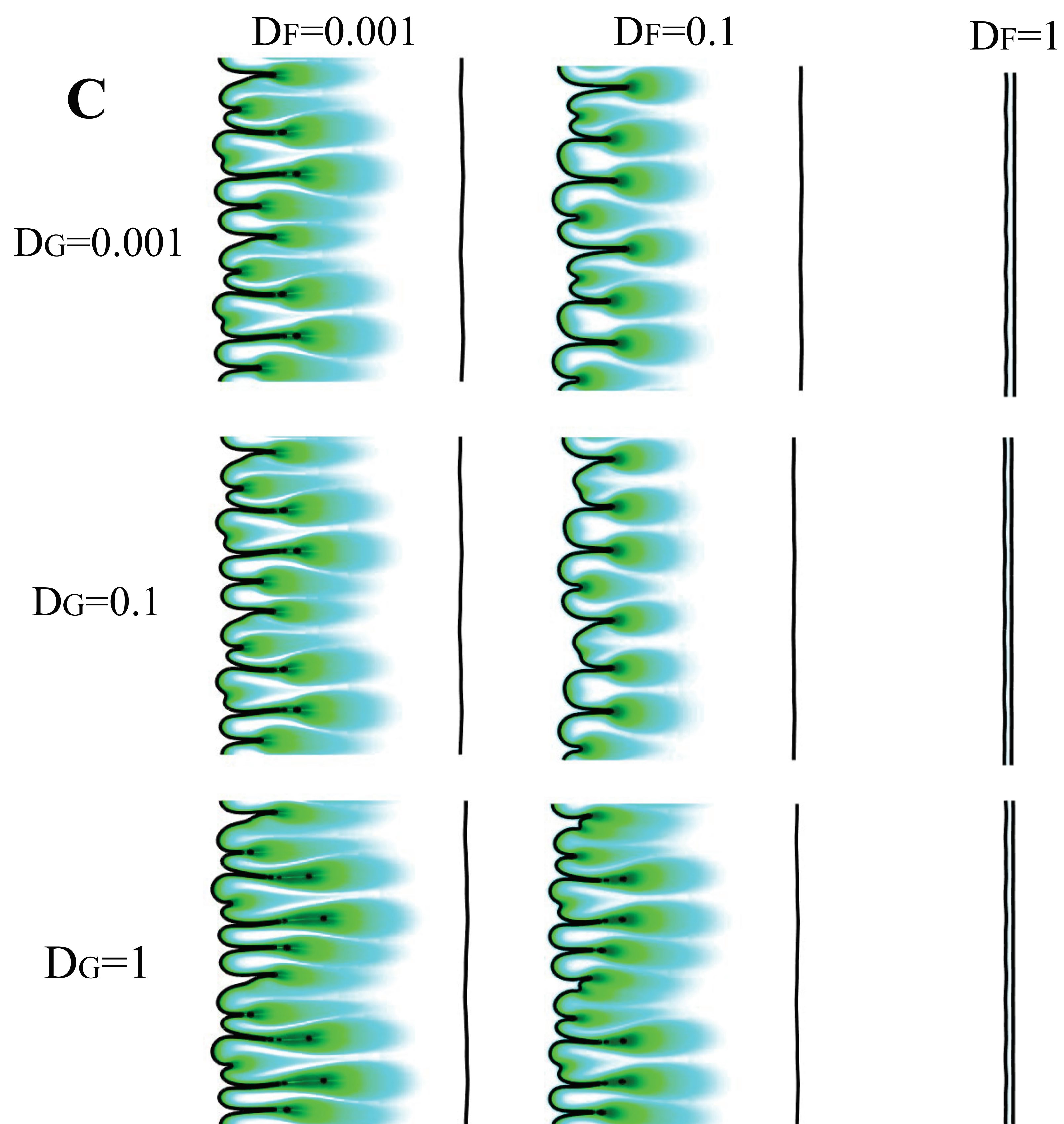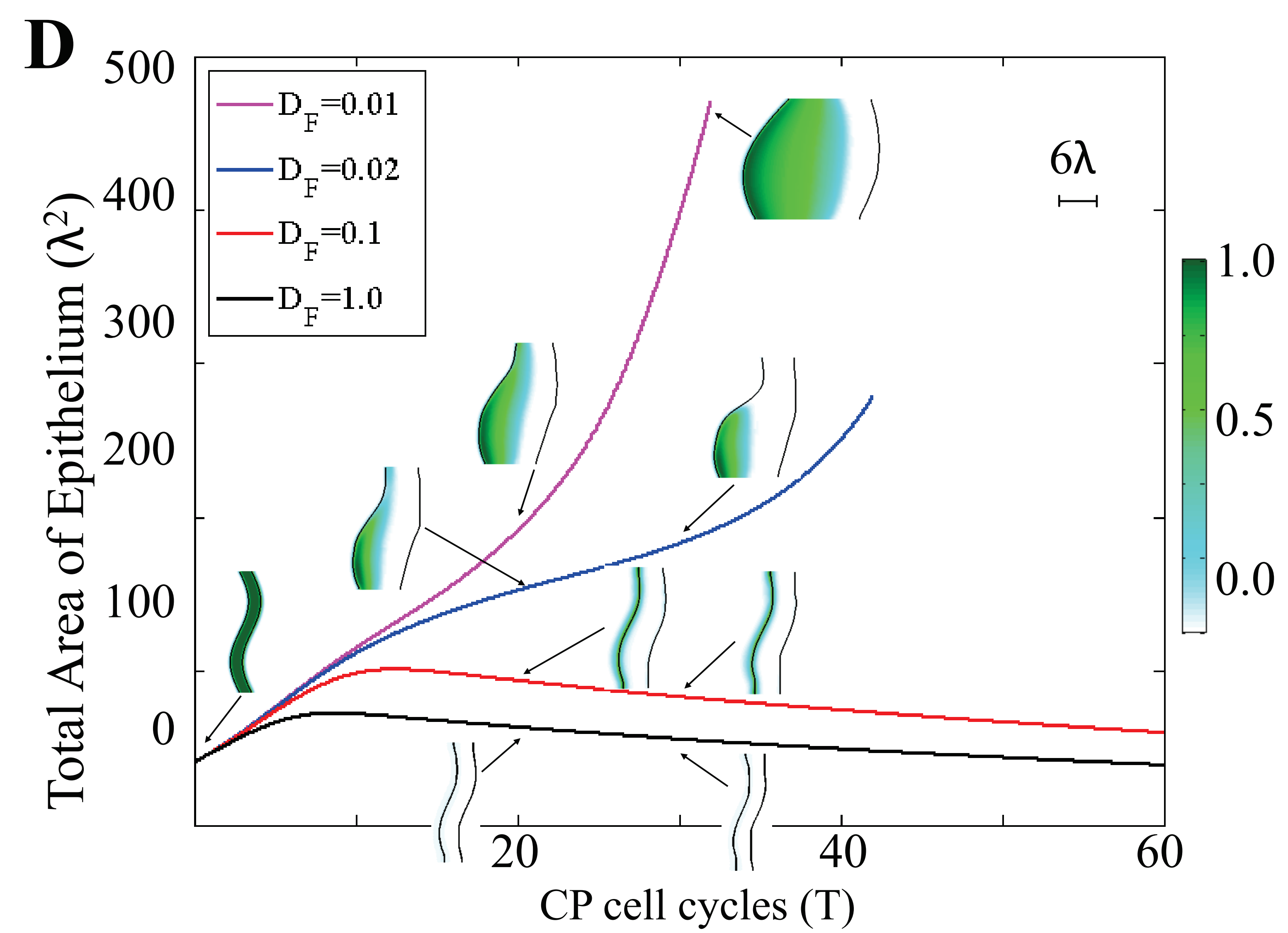

Supplement: S24 Fig — The evolution of epithelia and the distributions of endogenous positive feedback factors G (green). The black contour indicates the epithelial interface. The scale bar is proportional to the diffusion length (λ) of G, as labeled. In (A)-(B) panels, the equation defining the initial location of the BM membrane is given by 2.0 + 0.1[Sin2(3ξ) + Cos2(5 ξ) + Sin(13 ξ) + Cos(19 ξ) + Sin(23 ξ)] (see section 7 of S1 Text). In (A), the feedback gains are ϕ = 3.0 and γ = 5.0, i.e. ϕ/γ = 0.6. Snapshots of the simulation are shown that provide a more detailed view of the evolution in time for the simulation presented in the main text in (Fig 8H–8N). In (B) the feedback ratio is increased using ϕ = 3.5 and γ = 5.0, i.e. ϕ/γ = 0.7. This panel also shows a more detailed view of the evolution in time for the simulation presented in the main text in (Fig 8O–8U). In (C), the diffusivities of F and G are varied as labeled. Decreasing DF or increasing DG results in thinner fingers. Increasing DF makes F more diffusive, and the epithelium does not grow because the magnitude of F is too low to sustain growth. (D) The total areas of a sinusoidally perturbed epithelium as a function of time with different diffusivities of positive feedback factors, DF, as labeled. The perturbation amplitude is 1.5. When DF = 0.01 or 0.02, the area steadily increases and growth is sustained. In contrast, when DF = 0.1 or 1.0, the epithelium grows transiently, but growth is not sustained. Insets show the spatial distribution of positive feedback factors at T = 0, 20 and 30. When DF = 0.01 or 0.02, the epithelium forms a bud where positive feedback factors are concentrated (PDF) [file pcbi.1004814.s026.pdf]

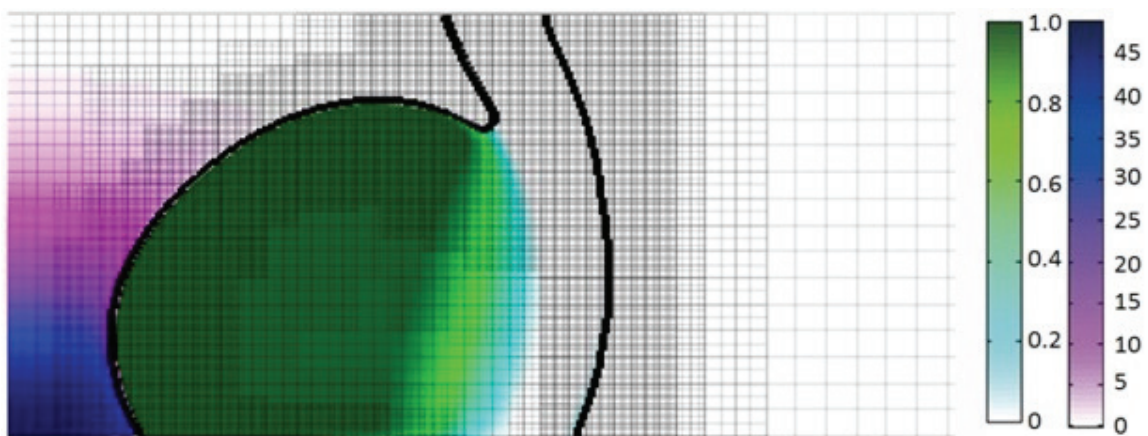

Supplement: S25 Fig — Exogenous positive regulator is shown with a blue-purple gradient, and endogenous positive regulator is shown with a green-cyan gradient. The black line indicates the χT = 0.5 contour. The coarsest level has 32×16 grids. There are three levels of refinement around the χT = 0.5 contour; each level has twice as many grid points as the parent level. (PDF) [file pcbi.1004814.s027.pdf]
